# Anaerobic metabolic evolution for homotypic L-valine fermentation

**DOI:** 10.1038/s41467-026-73619-7

**Published:** 2026-05-29

**Authors:** Siqi Yang, Fenghui Qian, Tao Wu, Bingbing Sun, Huiqi He, Meng Qiao, Feng Dong, Peng Gao, Zhao Chen, Ying Zhang, Junjie Yang, Yu Jiang, Sheng Yang

**Affiliations:** 1 Key Laboratory of Synthetic Biology, CAS Center for Excellence in Molecular Plant Sciences, Chinese Academy of Sciences, Shanghai, China; 2 Shanghai Research and Development Center for Industrial Biotechnology, Shanghai, China; 3 Meihua Holdings Group Co., Ltd., Langfang, China; 4 BBSRC/EPSRC Synthetic Biology Research Centre (SBRC), Biodiscovery Institute, School of Life Sciences, The University of Nottingham, Nottingham, United Kingdom; 5 School of Pharmaceutical Sciences, Fudan University, Shanghai, China; 6 College of Biotechnology, Fudan University, Shanghai, 201203 China; 7 Shanghai Taoyusheng Biotechnology Co., Ltd., Shanghai, China; 8 Present Address: Innovative Genomics Institute, University of California, Berkeley, Berkeley, CA USA

**Keywords:** Metabolic engineering, Metabolic engineering, Transposition, Synthetic biology

## Abstract

L-valine is an essential amino acid for animal nutrition. Ideally, it can be produced from D-glucose through homotypic L-valine fermentation in a growth-coupled manner. To date, no known microorganism, native or engineered, can grow on D-glucose and ammonia anaerobically with L-valine as the sole product. Here, we direct the metabolic flux through a reinforced L-valine synthetic pathway by blocking mixed-acid fermentation and L-alanine synthesis reactions to create an NADH driving force in *Escherichia coli*. We further evolve the engineered strain to debottleneck growth constraints by anaerobic growth rescue. The resulting evolved hyper-valine producer converts D-glucose in a 320 m^3^ reactor to 83.6 g/L L-valine within 60 h, reaching a yield of 0.55 g/g glucose (85% of the theoretical maximum). Through reverse engineering, we identify that more than a 10-fold improvement in anaerobic growth and L-valine production rate arises from the amplified L-valine synthetic pathway, the additional electron sinks and reprogramming of global regulation. Together, we changed the way of L-valine production into homotypic L-valine fermentation and demonstrate how *E. coli* variants adapted their metabolic activities and transcriptional regulation to boost fitness in an anoxic condition, with L-valine synthesis serving as the primary NADH-consuming pathway.

## Abstract (web summary)

L-valine is an essential amino acid for animal nutrition that can be produced through aerobic fermentation. Here the authors engineer *E. coli* for growth-coupled L-valine production under anaerobic conditions achieving a yield of 0.55 g/g glucose, 85% of the theoretical maximum.

## Introduction

L-valine ranks as the fifth limiting amino acid in swine and the fourth in poultry, playing a critical role in optimal protein deposition. Dietary L-valine supplementation in animal feed formulations allows a reduction in crude protein content without compromising growth performance. This approach not only lowers feed costs but also conserves natural resources required in agricultural feed production, contributing to reduced land use, greenhouse gas emissions, and risks of eutrophication and acidification. Consequently, targeted L-valine supplementation is essential in promoting the sustainable supply of animal protein to meet the demands of a growing global population^1,2^.

While L-valine is primarily produced through microbial fermentation from sugars by engineered *Corynebacterium glutamicum* or *Escherichia coli*^3–6^ under aerobic conditions, anaerobic microbial fermentations achieve high product yields and serve as a foundation for industrial bio-based processes^7^. An ideal L-valine synthetic pathway consists of four steps originating from pyruvate, two of which consume two moles of NADH generated during glycolysis while simultaneously generating ATP. This intrinsic redox-balancing feature theoretically enables homotypic L-valine fermentation (conceptually analogous to homolactic fermentation, in which a single product predominates as the predominant terminal product), thereby achieving the theoretical maximum yield (i.e., 1 mol of L-valine is stoichiometrically produced from 1 mol of glucose^8^)^9,10^ (Supplementary Fig. 1a). Improved yields have been achieved with harvested cells of extensive modified *C. glutamicum*^9,10^ or *E. coli*^11^ strains under oxygen deprivation or oxygen-limiting conditions. However, the aerobic growth phase required for biomass accumulation lowers the overall yield by consuming additional sugars and complicates the process. In contrast, during the anaerobic process, only a minimal portion of glucose is channeled toward biomass production. While *C. glutamicum* is incapable of anaerobic growth^12^, *E. coli* can grow under such conditions. This suggests that our current understanding remains insufficient for fully rationally engineering *E. coli* for homotypic L-valine fermentation and that directed evolution may serve as an effective strategy to bridge this gap.

Anaerobic growth rescue is a strategy that leverages microorganisms to restore or enhance growth under anaerobic conditions by maintaining intracellular redox balance^13^. This is generally achieved by coupling the desired reaction(s) to NAD^+^ regeneration, thereby alleviating the redox imbalance caused by the inactivation of NADH-consuming pathways^13^. This strategy has enabled the transformation of *E. coli* from mixed-acid fermentation^14^ to the production of n-butanol^13^, D-lactate^15^, succinate^16^, ethanol^17,18^, and L-alanine^19^. However, anaerobic fermentation production at a commercial scale has been only successfully implemented for L-alanine among the 20 amino acids^20^. Nevertheless, the inherent redox-balancing capacity of L-valine biosynthesis (Supplementary Fig. 1a) supports the theoretically feasibility of developing an anaerobic fermentation approach for its production. However, this remains unrealized, possibly due to the substantially greater complexity of the L-valine biosynthetic pathway compared to that of L-alanine.

In this work, we delete the NADH-consuming reactions such as mixed-acid fermentation reactions in *E. coli* to impair its anaerobic growth, then we enhance the L-valine biosynthesis pathway^4–6^ to create a driving force for reactions that consume NADH. By subjecting the strain to anaerobic adaptive laboratory evolution, we develope a high-yield strain, increasing the production rate from 0.10 g/L/h to over 1.00 g/L/h. The evolved strain achieves 83.6 g/L of L-valine in a 320 m³ industrial fermenter within 60 h, reaching a yield of 0.55 g/g (85% of the theoretical maximum). Through multiple rounds of resequencing and iterative re-evolution using the Design–Build–Test–Learn framework (Supplementary Fig. 2), we identify several copy number variations and single-nucleotide variants, gaining insights into the genetic mechanisms underlying its high yield. Collectively, we demonstrate a growth-coupled homotypic L-valine fermentation strategy and uncover the metabolic and regulatory reprogramming that supports efficient production under anoxic conditions, positioning L-valine synthesis as the principal NADH-consuming reaction.

## Results

### Rational design of S1.0

**Fig. 1:**
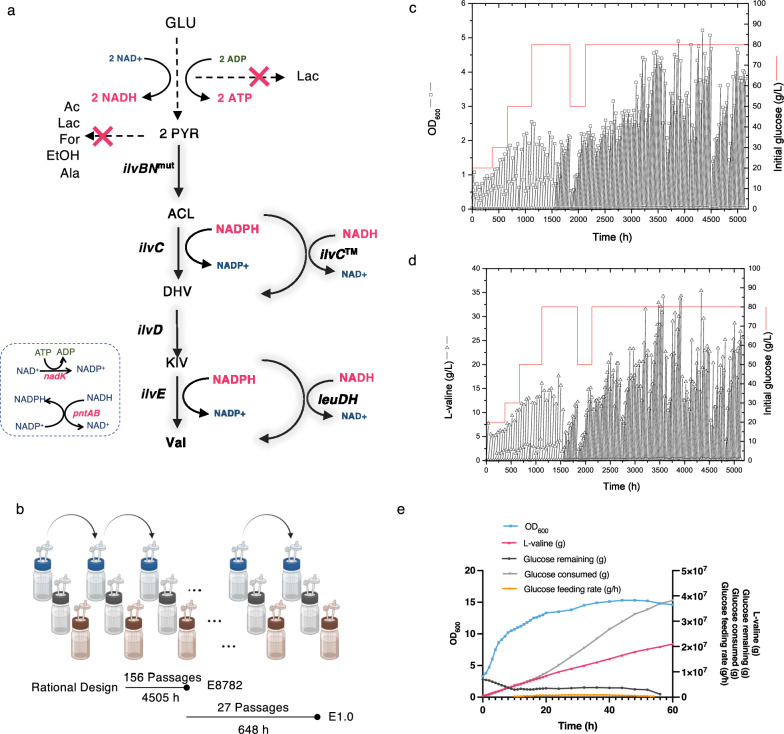
A highly productive L-valine strain derived from anaerobic laboratory evolution of the rationally designed strain S1.0. **a** Schematic overview of the systems metabolic engineering strategies for anaerobic L-valine production in *E. coli*. A total of two NADH and two ATP molecules are generated, and two NADH molecules are consumed during the biosynthesis of one L-valine molecule from glucose under anaerobic conditions. Shadowed arrows indicate the enhanced fluxes, while pink Xs denote removed inhibitory or repressive elements (*avtA, ldhA, mgsA, frd, pflB, adhE*, and *ackA*). The detailed map is shown in Supplementary Fig. 1b. Metabolites include GLU (glucose), PYR (pyruvate), ACL (α-acetolactate), DHV (α,β-dihydroxyisovalerate), KIV (α-ketoisovalerate), Val (L-valine), Ac (acetate), Lac (lactate), EtOH (ethanol), For (formate), and L-Ala (L-alanine). **b** Flowchart illustrating the construction and adaptive evolutionary process of the anaerobic L-valine hyperproducer strain. More details are in Supplementary Fig. 4a. Created with BioRender. Yang, S. (2026) https://BioRender.com/366ra6p. **c** Growth curve of the S1.0 throughout evolutionary passages with increasing glucose concentrations. A magnified view is provided in Supplementary Fig. 4g. **d** Evolutionary trajectory of S1.0 during serial passages. A magnified view is provided in Supplementary Fig. 4h. **e** Anaerobic fermentation performance of E1.0 in a 320 m³ industrial fermenter. When the residual glucose concentration reached ~15 g/L (9–10 h), a 60%–62% (w/v) glucose solution was fed at 0.5–1.5 m³/h to maintain a 10–15 g/L residual glucose level. Feeding was terminated at 52–54 h, and residual glucose was fully consumed at the end of fermentation (60 h). Source data for this figure are available in the Source Data file.

The biosynthesis of L-valine from pyruvate in *E. coli* begins with acetohygroxy acid synthase (AHAS), a heterodimer encoded by *ilvB* and *ilvN*, which condenses two pyruvate molecules into acetolactate, a step subject to feedback inhibition by L-valine. The substitution of three amino acids (G20D, V21D, and M22F) in IlvN can resist the feedback inhibition of L-valine, the mutant *ilvBN* genes called *ilvBN*^mut^^4^. Subsequently, acetolactate is reduced by acetohydroxyacid isomeroreductase (AHAIR, encoded by *ilvC*), using NADPH to form 2,3-dihydroxyisovalerate, which is then dehydrated by dihydroxyacid dehydratase (DHAD, encoded by *ilvD*), to produce 2-ketoisovalerate. In the final transamination step, branched-chain amino acid transaminase (BCAATA, encoded by *ilvE*) converts 2-ketoisovalerate into L-valine using L-glutamate as the amino group donor and pyridoxal phosphate as a cofactor, simultaneously producing α-ketoglutarate. Both *ilvC* and *ilvE* prefer NADPH as the cofactor (Fig. 1a and Supplementary Fig. 1b). This pathway is tightly regulated, with each enzymatic step carefully controlled by genetic and metabolic mechanisms to ensure efficient and precise L-valine synthesis.

To leverage anaerobic growth rescue for homotypic L-valine fermentation, we focused on the two key reductive steps that can consume NADH generated from glycolysis: the reduction of α-acetolactate and reductive amination/transamination of α-ketoisovalerate. To utilize the NADH generated by glycolysis and abolish the transformation from L-valine to L-alanine by *avtA*, we inserted a copy of AHAIR triple mutant IlvC^TM^ (S34G, L48E, R49F) from *Corynebacterium glutamicum*^9^ at *ldhA* locus and a copy o*f leuDH* from *Bacillus licheniformis*^10,21^ functioned as reductive amination, both naturally prefer NADH. To enforce coupling between L-valine production and NADH oxidation, we deleted the competitive NADH-consuming pathways involved in mixed-acid fermentation (ethanol, acetate, lactate, succinate, and formate) by knocking out *adhE*, *ackA*, *ldhA*, *mgsA*, *frd*, and *pflB*^11,19^ in *E. coli*, thereby making L-valine biosynthesis the primary route for intracellular redox balancing. In this design, we aimed to couple ATP generation from glycolysis to NADH oxidation via L-valine synthesis, thereby enabling growth-coupled selection for strain improvement through anaerobic adaptive laboratory evolution (Fig. 1a and Supplementary Fig. 1b). Simultaneously, we integrated a copy of *ilvBN* mutant, *ilvBN*^mut^, that is resistant to L-valine inhibition at the *adhE* locus and a copy of *ilvED* at the *ackA* locus to enhance the terminal pathway. Furthermore, we enhanced the expression of *ygaZH* (branched-chain amino acid exporter) via integration at *the frd* locus controlled by a strong promoter Ptac, as well as the global regulator *lrp* at *mgsA* locus, which has been reported to upregulate both *ilvBN* and *ygaZH*^4^. The resulting strain, named S8975 (Δ*avtA*::Ptac-*leuDH*, Δ*ldhA*::Ptac-*ilvC*^TM^, Δ*mgsA*::Ptac-*lrp*, Δ*frd*::Ptac-*ygaZH*, Δ*pflB*, Δ*adhE*::Ptac-*ilvBN*^mut^, Δ*ackA*::Ptac-*ilvED*) (S refers to synthesized) (Supplementary Data 1), was subjected to anaerobic adaptive laboratory evolution through serial passaging (Fig. 1b). Unexpectedly, after 12 passages, the L-valine titer was only 0.6 g/L, whereas L-alanine accumulated to 1 g/L. This limited coupling between growth and L-valine production may be attributed to two factors. First, the anaerobic adaptive evolution regime may not be efficient. Second, residual L-alanine synthesis via *alaA* and *alaC* likely diverted pyruvate flux (Supplementary Fig. 1b), resulting in L-alanine as the dominant fermentation product.

Guided by the accumulation of L-alanine, we next sought to validate the effectiveness of anaerobic adaptive laboratory evolution method using the successful case of L-alanine^19^. In this system, the native D-lactate dehydrogenase of *E. coli* was replaced with the NADH-dependent alanine dehydrogenase *alaD* from *Geobacillus stearothermophilus* to link cell growth with L-alanine production (Supplementary Fig. 1c). To implement this design, we replaced *ilvBN*^mut^ with *alaD* at the *adhE* locus in strain S8975, placing *alaD* is under the control of the promoter of *ldhA*^19^, to generate strain S9015 (S8975Δ*ilvBN*^mut^::PldhA-*alaD*) (Supplementary Data 1). This strain was then subjected to 86 passages of anaerobic adaptive evolution (Supplementary Fig. 3a–c). After single-colony isolation, strain E8482 (E refers to evolved) was obtained, which consumed 104 g/L of glucose over 43 h and produced 77 g/L of L-alanine (Supplementary Fig. 3d). The glucose consumption rate was 2.41 g/L/h, and the yield was 0.86 g/g. This result indicates that our anaerobic adaptive laboratory evolution method is effective in achieving high L-alanine titer and production rate anaerobically. To gain insight into the mechanisms of this strain, whole-genome sequencing was carried out and revealed that the mutations *alaE* (p.A48S) and *alaE* (p.A149D) are likely key contributors to the increased L-alanine production, as discussed in subsequent sections.

With the effectiveness of the anaerobic adaptive laboratory evolution strategy validated, the remaining limitation was attributed to the diversion of pyruvate flux toward L-alanine synthesis. To eliminate the by-production of L-alanine and make L-valine the sole product in the evolved S8975, we further deleted two L-alanine aminotransferases, *alaA* and *alaC*^22^, which are involved in L-alanine synthesis (Supplementary Fig. 1b). As expected, the resulting strain S8975Δ*alaA*Δ*alaC* (Supplementary Data 1) no longer produced L-alanine, indicating the accumulation of L-alanine was eliminated. To support cell growth in the absence of endogenous L-alanine synthesis, a small amount of L-alanine (0.1 g/L) was supplemented under laboratory-scale conditions, as strains lacking *avtA*, *alaA*, and *alaC* exhibit significantly reduced growth compared to the wild type^23^. Additionally, we strengthened L-valine synthesis by reinforcing the terminal pathway via the addition of a copy of *ilvC*^TM^, *leuDH*, *ilvBN*^mut^, *ilvC*, and *ilvD* on the chromosome.

Because S8975 failed to improve under anaerobic adaptive laboratory evolution, we speculate that growth was not optimally coupled to NADH reoxidation in the current design. Since we did not delete the endogenous IlvC (for α-acetolactate reduction) and IlvE (for α-ketoisovalerate transamination) which are NADPH-dependent, whereas IlvC^TM^ and LeuDH are NADH-dependent, we introduced two cofactor-balancing enzymes encoded by *pntAB*^9,24^ and *nadK*^25,26^ to better balance NAD(H) and NADP(H) availability. *pntAB* encodes pyridine nucleotide transhydrogenase, which can catalyze the reversible transfer between NADH and NADP^+^ (Fig. 1a). *nadK* encodes NAD^+^ kinase, which catalyzes the phosphorylation of NAD^+^ to NADP^+^ using ATP (Fig. 1a). This design allows the strain to choose the optimal ratio between NAD(H) and NADP(H). With these optimizations, the resulting strain S1.0 (Supplementary Data 1) improved its L-valine performance, achieving an L-valine titer of 6.4 g/L and an OD_600_ of approximately 0.68 under anaerobic fermentation conditions within 42 h (production rate: 0.15 g/L/h; yield: 0.55 g/g glucose), indicating that S1.0 is an ideal candidate of anaerobic adaptive laboratory evolution.

### Anaerobic adaptive laboratory evolution of S1.0

S1.0 was subjected to anaerobic adaptive laboratory evolution through serial passaging. During the initial phase, cells were cultured in NBSAG20 medium (20 g/L glucose) with a 1% (v/v) inoculum and passaged every 48 h. After eight passages, residual glucose dropped below 10 g/L, biomass (OD_600_) increased by approximately 50%, and L-valine production increased by about 20% (Fig. 1c, d), indicating that anaerobic growth was rescued via L-valine production.

To simulate industrial conditions and shortened subculture intervals, as part of the anaerobic metabolic evolution strategy, we gradually increased the glucose concentration to promote mutation by maintaining high cell division activity. In general, when over half of the glucose was consumed, we raised the initial concentration. Once it reached 80 g/L, we further reduced the passage duration. Gradually, the initial glucose concentration was raised to 80 g/L, and the passage interval was reduced to 24 h after 33 passages in total.

After another 24 passages, the strain produced over 15 g/L of valine in 24 h, with an OD_600_ exceeding 2. After an additional 27 passages, the medium was switched to a low-salt AM1 medium to minimize the utilization of salts, with continued passaging for an additional 72 passages. After this final round, L-valine production in 24 h exceeded 22 g/L, with a glucose consumption rate of approximately 2 g/L/h and a yield around 0.50 g/g. A representative clone, E8782, was isolated by single-colony selection (Supplementary Data 1 and 2).

E8782 was further passaged 27 times, achieving a L-valine titer exceeding 25 g/L in 24 h, with a production rate surpassing 1 g/L/h, a glucose consumption rate over 2 g/L/h, and a yield above 0.50 g/g. This led to the single-colony isolation of E1.0 (Fig. 1b–d and Supplementary Data 1 and 2).

The evolution journey to obtain E1.0 took 5153 h with 183 passages, with the production rate increasing from 0.10 to 1.21 g/L/h (Fig. 1b–d and Supplementary Fig. 4a, g, h). In a 3 L fermenter, E1.0 reached an L-valine titer of 72 g/L in 60 h, with a yield of 0.55 g/g (Supplementary Fig. 4b, c). In a 320 m³ industrial fermenter using fed-batch fermentation, E1.0 achieved an L-valine titer of 83.6 g/L in 60 h, with a production rate of 1.39 g/L/h and visible crystals forming in the fermentation broth, maintaining the high yield which reached 85% of the theoretical maximum (1 mol/mol glucose; 0.65 g/g glucose) (Fig. 1e; see also Supplementary Fig. 4d, e for the other two batches; Supplementary Fig. 4f; yields calculations for three batches in Supplementary Data 3). During the exponential phase (1–6 h), the specific growth rate was 0.183 h⁻¹, the specific L-valine production rate was 9.48 mmol/gDCW/h and the specific glucose uptake rate was 6.41 mmol/gDCW/h (conversion factor, 1 OD_600_ = 0.275 gDCW/L, was used)^6^.

Furthermore, by improving cell growth through low-rate aeration (0.05 vvm) during the first 4 h, we successfully shortened the fermentation time from 60 to 45 h while maintaining a similar titer (82.6 g/L) and yield (0.56 g/g). This optimization increased production rate to 1.84 g/L/h, representing a 34% improvement compared to the fully anaerobic process.

### Stoichiometric validation of biomass formation and redox balance of E1.0 during fed-batch fermentation

To quantitatively validate biomass formation and product yield under fed-batch conditions, we performed 10 L scale fermentations under process-relevant conditions (Supplementary Fig. 5a–c). Concentrations of L-valine and major byproducts, together with cumulative glucose feeding profiles, were monitored throughout fermentation (Supplementary Data 4). In addition to L-valine, the main detectable byproducts were L-threonine, L-isoleucine, L-alanine, L-leucine, and acetate. Based on the measured metabolite profiles, we performed a complete carbon recovery analysis and a stoichiometric redox balance by accounting for NAD(P)H generation, and consumption inferred from product fluxes (Supplementary Data 4 and Supplementary Fig. 6). The net reducing equivalent balance was slightly negative, which can be reconciled by considering reducing equivalents generated during biomass formation, consistent with previous reports on anaerobic *E. coli* growth^27^. Carbon reconciliation further showed that residual glucose available for biomass synthesis yielded *Y*_*x*_/_*s*_ values of 0.13–0.17 gDCW/g glucose, in line with reported anaerobic growth yields and substantially lower than aerobic values^27^. Together, these results demonstrate that the observed product yields and biomass formation are consistent with stoichiometric mass and redox constraints.

To examine whether the observed redox balance was reflected at the transcriptional level, we analyzed RNA-seq data focusing on genes involved in NADH and NADPH utilization. Transcriptomic analysis revealed gene dosage–dependent upregulation of *ilvC* (*P* = 4.91E−41) and leuDH* (*P* = 8.94E−19), whereas *pntAB* (*pntA*: *P *= 9.63E−01; *pntB*: *P *= 2.91E−02) and *nadK* (*P* = 2.23E−01) remained unchanged after evolution. These transcriptional changes indicate that both NADPH-utilizing (*ilvC*) and NADH-utilizing (leuDH*) pathways were reinforced in the evolved strain, thereby enhancing metabolic fitness under selective conditions (Supplementary Data 5 and 6). These observations support our NADH/NADPH utilization model, in which both cofactors are concurrently exploited.

### Genomic analysis of E1.0

To identify the genomic changes responsible for the enhanced fermentation performance of E1.0, we performed whole-genome sequencing on both E1.0 and its predecessor, E8782. Sequencing analysis of E8782 revealed a duplication in the region from *EcolC_4160* to *EcolC_4238 in E. coli* ATCC 8739 (Accession Number: CP000946), which includes *ilvC* and *ilvXGMED*. This duplication resulted in an increase in the copy number of *ilvC* from two to three, while the copy numbers of *ilvE* increased from three to four and *ilvD* from two to three, respectively. In S1.0, a copy of *ilvC* driven by Ptac promoter with amplification arms was integrated at *yjcS*, which was further amplified to four copies through its amplification arms in E8782, and in combination with the previous duplication, this resulted in a total of six copies. During subsequent evolution from E8782 to E1.0, an additional large genomic fragment (*EcolC_0117*–*EcolC_0153*) was duplicated. Within this region, at the *avtA* locus, the 22nd amino acid of LeuDH was mutated from valine to isoleucine (LeuDH^V22I^), which was subsequently duplicated into two copies. Together with a wild type *leuDH*, the final copy of *leuDH* and *leuDH*^V22I^ reaches three (Supplementary Data 1, 7, and 8).

These sequencing results suggest that AHAIR encoded by *ilvC* and L-leucine dehydrogenase encoded by *leuDH* are the rate-limiting enzymes for anaerobic L-valine production in *E. coli*, with *ilvXGMED* potentially contributing as well.

### Characterization of LeuDH^V22I^

**Fig. 2:**
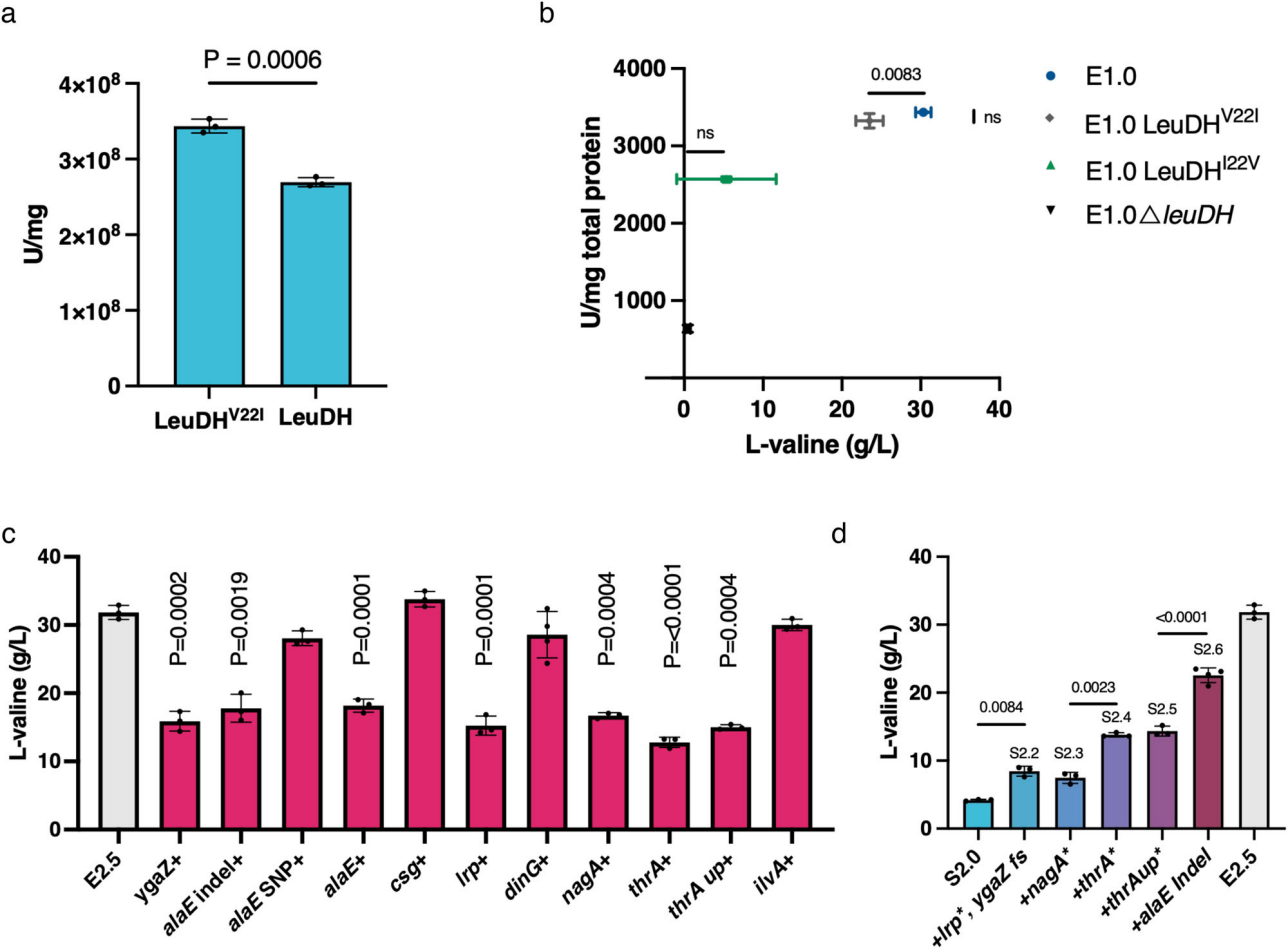
Enzymatic activity and genetic validation of mutants identified during anaerobic adaptive laboratory evolution. **a** Specific enzymatic activities of purified LeuDH and LeuDH^V22I^. **b** Correlation of L-valine production and specific activity of crude cell extracts from E1.0, E1.0 LeuDH^V22I^, E1.0 LeuDH^I22V^, and E1.0∆*leuDH*. The *P* value for the specific activity of crude cell extracts between E1.0 and E1.0 LeuDH^V22I^ was 0.1860, and the *P* value for L-valine titer between E1.0 LeuDH^I22V^ and E1.0ΔLeuDH was 0.3098. **c** Reverse mutation analysis of ten mutations identified in E2.5. For *dinG*+, *n* = 4 biological replicates. **d** Impact of combining six mutations (*lrp* (p.P139L), *ygaZ* (p.W152fs), *thrA* (upstream G-A), *thrA* (p.S354Y), *nagA* (p.G141fs), *alaE* (p.S142_Q145delinsK)) on L-valine production. For S2.6, *n* = 4 biological replicates. In all panels, data are shown as mean ± SD (*n* = 3 biological replicates) unless otherwise stated, where each replicate represents an independently grown culture. Statistical significance was assessed using a two-sided Welch’s *t*-test. Exact *P* values are indicated in each panel or reported in the figure legends. Source data, more results of pairwise comparisons, including *P* values, *t* statistics, degrees of freedom (df), and 95% confidence intervals, are provided in the Source Data file.

To assess the functional impact of the LeuDH^V22I^ variant, we purified both LeuDH and LeuDH^V22I^ (Supplementary Fig. 7) and found that the specific activity of LeuDH^V22I^ was 30% higher than that of wild-type LeuDH, indicating that the valine-22-isoleucine mutation enhanced specific activity (Fig. 2a).

To confirm the beneficial effect of the LeuDH^V22I^ in L-valine synthesis, we reversely mutated the two copies of LeuDH^V22I^ back to wild-type LeuDH, resulting in E1.0 LeuDH^I22V^. The L-valine titer dropped to below 10 g/L, and the specific L-leucine dehydrogenase activity of crude cells also decreased, further validated our in vitro evidence that LeuDH^V22I^ is crucial for the enhanced activity of LeuDH in L-valine production (Fig. 2b).

To determine if mutating the remained one copy of wild-type LeuDH located at the *lacI* locus in E1.0 to LeuDH^V22I^ could further improve L-valine production, we mutated it to construct E1.0 LeuDH^V22I^. The specific activity of LeuDH in this strain was comparable to that of E1.0 and the titer of L-valine was even lower than E1.0, suggesting that two copies of LeuDH^V22I^ were sufficient and that additional mutated copies were redundant (Fig. 2b).

To verify the essentiality of LeuDH in L-valine synthesis, we also knocked out one wild-type LeuDH and two copies of LeuDH^V22I^ in E1.0, constructing E1.0∆*leuDH*. In this strain, the specific L-leucine dehydrogenase activity of crude cells decreased by more than 90% compared to E1.0, and the L-valine titer was almost zero (Fig. 2b). These results indicate that LeuDH^V22I^ enhances the specific activity of LeuDH further increasing L-valine production.

### Rapid construction and anaerobic growth rescue of S2.0

Based on the resequencing results of E1.0, we aimed to construct a genotype-minimized strain with a similar phenotype by integrating a single copy of the *ilvXGMED* expression cassette, multicopy *ilvC*, and *leuDH*^V22I^ into S1.0 to construct S2.0 (Supplementary Data 1). To accelerate the reconstruction, we also leveraged CRISPR-associated transposase (CAST)^28–30^ for multicopy insertions. However, anaerobic fermentation of three S2.0 colonies resulted in lower rates (0.24, 0.35, 0.27 g/L/h) than E1.0, suggesting that rational design alone was insufficient and that additional, unidentified bottlenecks remained.

To address this, we subjected S2.0 to anaerobic adaptive laboratory evolution. Gradual reduction in passage duration and increase in glucose concentration led to continuous improvement in L-valine production. After 163 passages within 4778 h, the strain achieved a titer exceeding 30 g/L, a production rate of 1.40 g/L/h, and a biomass (OD₆₀₀) greater than 7 (Supplementary Fig. 8a, b). A high-producing colony showing no changes in gene copy number was isolated and designated E2.5 (Supplementary Data 1). This strain produced 33 g/L L-valine within 24 h. E2.5 evolved faster than strain E1.0, suggesting that enhanced expression of *ilvC* and *leuDH*^V22I^ in S2.0 facilitated evolutionary progress, though alone they were insufficient to reach the E1.0-level phenotype.

### Genome analysis of E2.5 and secondary reconstruction

Whole-genome resequencing of E2.5 revealed ten single-nucleotide variants or indels relative to S2.0, including *csgB* (upstream G-A), a copy of *ygaZ* (p.W152fs), *dinG* (p.P509S), *thrA* (upstream G-A), *thrA* (p.S354Y), *ilvA* (p.S438I), *alaE* (p.A48T), *alaE* (p.S142_Q145delinsK), a copy of *lrp* (p.P139L), and *nagA* (p.G141fs) (Supplementary Data 1, 7, 8, and 9).

To assess the contribution of the ten variants, we individually reverted each mutation, including the combination of two in *alaE*. Among these, reversion of *ygaZ* (p.W152fs), *alaE* (p.S142_Q145delinsK), *lrp* (p.P139L), *nagA* (p.G141fs), *thrA* (upstream G-A), and *thrA* (p.S354Y) resulted in an obvious decrease in L-valine production. Reversions in *dinG* (p.P509S), *ilvA* (p.S438I), and *alaE* (p.A48T) led to slight decreases, while reverting *csgB* (upstream G-A) resulted in a slight increase in production, though this change was not statistically significant (Fig. 2c).

We next combined the six major beneficial mutations into S2.0 to evaluate whether a genetically defined strain could recapitulate the E2.5 phenotype. Among them, *lrp* (p.P139L) draws particular interest, as this global regulator has been reported to upregulate AHAS I (*ilvBN*) and III (*ilvIH*) expression, enhance YgaZH-mediated L-valine export, and downregulate the import protein LivJ^4,5^. Overexpression of Lrp has previously reported to improve aerobic L-valine production by 22%^5^. Based on these findings, we introduced the *lrp* (p.P139L) into S2.0, unexpectedly co-introduced *ygaZ* (p.W152fs), generating S2.2 (Supplementary Data 1). S2.2 exhibited a twofold increase in L-valine titer (from 4 to 9 g/L), indicating that *lrp* (p.P139L) modulates global transcription and improves L-valine production independently of YgaZH overexpression (Fig. 2d).

We subsequently introduced in situ mutations on *nagA* (p.G141fs), *thrA* (upstream G-A), *thrA* (p.S354Y), and *alaE* (p.S142_Q145delinsK) into S2.2 to generate S2.6 (Supplementary Data 1), which achieved an average titer of 23 g/L, approximately 70% of E2.5 (Fig. 2d). To fully reconstruct E2.5, we constructed S2.10, which incorporated all ten mutations (Supplementary Fig. 8c and Supplementary Data 1). Unexpectedly, its titer (~19 g/L) was lower than that of S2.6, suggesting possible epistatic effects linked to the mutational order or context. Given reverting *csgB* (upstream G-A) resulted in a slight increase in production, we further reversed *csgB* (upstream G-A) in S2.10, but the titer (~20 g/L) was still lower than that of S2.6.

As for the four mutations that were selected during evolution but did not enhance production upon reconstruction, we speculate that the protein encoded by *dinG* (p.P509S) belonging to the member of helicase superfamily 2, which plays a key role in the DNA repair process, was selected for its potential role in enhancing strain evolvability rather than directly contributing to the L-valine phenotype^31^. The mutation in *csgB*, encoding the curli subunit protein that helps bacteria adhere to and aggregate on solid surfaces^32^, may contribute to cheaters cells adhering to the evolution fleakers. This adherence could stimulate growth-coupled L-valine production by allowing non-producers to proliferate alongside producers. The contributions of *ilvA* (p.S438I) and *alaE* (p.A48T) will be explored further in subsequent sections.

### Lrp^P139L^ globally regulates cellular metabolism, enhancing L-valine production

**Fig. 3:**
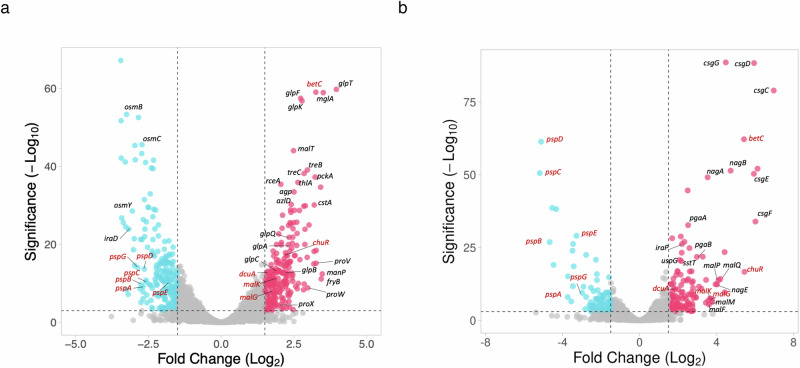
Transcriptional profile of the synthesized and evolved strains. **a** Volcano plots showing genome-wide transcriptional differences between S2.1 and S2.0 (mean of *n* = 3 biological replicates). **b** Volcano plots showing genome-wide transcriptional differences between E2.5 and S2.0 (mean of *n* = 3 biological replicates). Genes that are commonly upregulated or downregulated are highlighted in red. Source data for this figure are available in the Source Data file.

To evaluate the role of Lrp^P139L^, we constructed S2.1 by reverting *ygaZ* (p.W152fs) in S2.2, resulting a strain carrying only the *lrp* (p.P139L) compared to S2.0. Transcriptomic analysis of S2.1 and S2.0 revealed that Lrp^P139L^ upregulated a set of carbon utilization genes while downregulating osmotically inducible (osm) proteins and phage shock proteins (Fig. 3a and Supplementary Data 10), likely enhanced carbon source utilization and increased sensitivity to hyperosmotic stress^33^, thereby boosting L-valine production.

We also analyzed the transcriptome of E2.5 (Supplementary Data 11). Phage shock proteins, maltose metabolism genes (*malK* and *malG*), as well as *betC*, *chuR*, and *dcuA*, represent the intersecting genes (Fig. 3b). *betC* and *chuR* belong to an operon, which appears to be both utilized for osmoprotection or S replenishment^34^. *dcuA* is an anaerobic C4-dicarboxylate transporter, which is required for the uptake of L-aspartate^35^. We speculate that upregulating *dcuA* can enhance the uptake of L-aspartate, thereby increasing precursor availability for L-threonine synthesis (which is discussed later in subsequent sections). These results suggest that Lrp^P139L^ downregulates phage shock proteins to maintain a high growth rate and metabolic activity at the expense of reduced stress tolerance, while upregulating carbon source utilization genes to enhance carbon uptake.

**Fig. 4:**
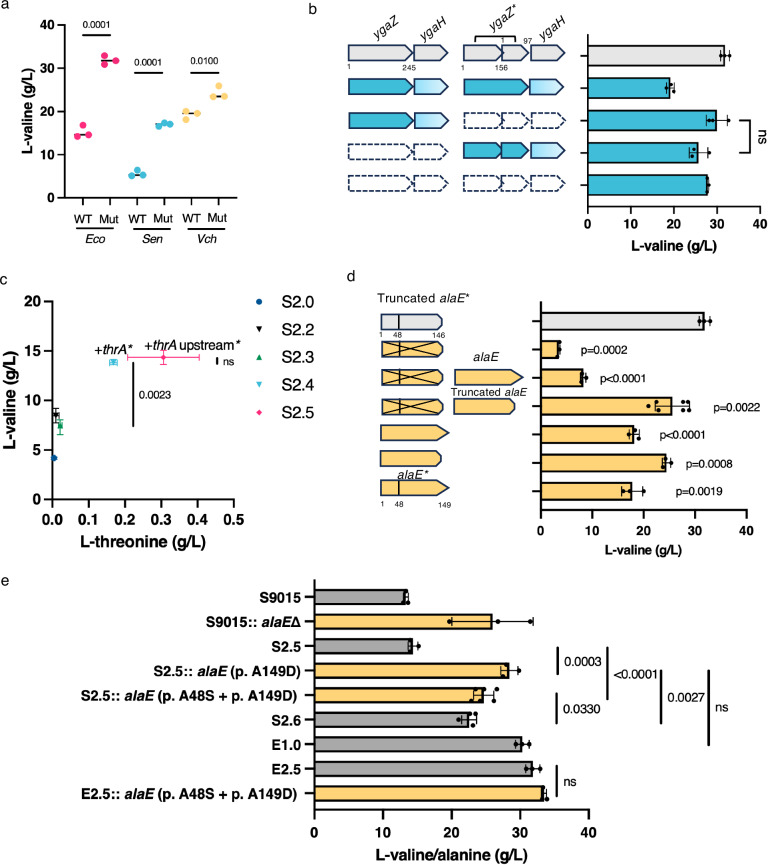
Mechanistic characterization of *lrp* (p.P139L), *ygaZ* (p.W152fs), *thrA* (upstream G-A), *thrA* (p.S354Y), and *alaE* (p.S142_Q145delinsK). **a** Functional comparison of *lrp* and *lrp* (p.P139L) from *E. coli*, *V. cholerae*, and *S. enterica* in L-valine production. **b** L-valine production associated with reverse mutation of *ygaZ* (p.W152fs), knockout of Ptac-*ygaZ* (p.W152fs)–*ygaH*, and individual or combined knockout of the native *ygaZH*. Hollow dashed lines indicate gene knockouts. Gray pentagons and bars denote the genotype and phenotype of E2.5. The *P* value between E2.5ΔPtac-ygaZ* and E2.5ΔPyga-*ygaZ* was 0.0870. **c** Impact of *thrA* (p.S354Y) and *thrA* (upstream G-A) mutations on L-threonine and L-valine production. The *P* value for L-valine titer between S2.4 and S2.5 was 0.3238. **d** L-valine production strains carrying different *alaE* variants compared with E2.5 (gray pentagons and bars). The evolved strain harbors two mutations in the *alaE* gene: *alaE* (p.A48T) (depicted as a short vertical line) and *alaE* (p.S142_Q145delinsK) (depicted as a hexagon). A crossed hexagon indicates a loss-of-function mutation. Significance levels indicate statistical differences relative to E2.5. For the E2.5 strain harboring a cis loss-of-function mutation in *alaE* (p.A48T and p.S142_Q145delinsK) and a trans-complemented *alaE* (p.S142_Q145delinsK), *n* = 7 biological replicates. **e** Cross-validation of the *alaE* (p.S142_Q145delinsK) and *alaE* (p.A48S, p.A149D) in L-alanine- and L-valine-producing strains, respectively. S2.5::*alaE* (p.A149D) reached to a level of L-valine production comparable to E1.0 (*P* = 0.1095). For S2.5::*alaE* (p.A48S + p.A149D), *n* = 6 biological replicates. For S2.6, *n* = 4 biological replicates.In all panels, data are shown as mean ± SD (*n* = 3 biological replicates) unless otherwise stated, where each replicate represents an independently grown culture. Statistical significance was assessed using a two-sided Welch’s *t*-test. Exact *P* values are indicated in each panel or reported in the figure legends. Source data, more results of pairwise comparisons, including *P* values, *t* statistics, degrees of freedom (df), and 95% confidence intervals, are provided in the Source Data file.

To test whether the P139L mutation has conserved effects in Lrp homologs, we replaced Lrp^P139L^ in E2.5 with orthologs from *Vibrio cholerae* (WP_032476343.1) and *Salmonella enterica* (EBJ0658416.1), along with their respective P139L mutants. The P139L variants consistently improved L-valine production (Fig. 4a), suggesting that this substitution can reshape transcriptional regulation across diverse Lrp homologs.

### Additional L-valine export proteins beyond YgaZH

Previous studies indicate that overexpressing *ygaZH* enhances L-valine export^4^. Following this rationale, we introduced an additional copy of *ygaZH* under a Ptac promoter into the rationally designed S1.0. After anaerobic adaptive laboratory evolution, a frameshift mutation in *ygaZ* occurred in E2.5, and reverting this mutation led to nearly a 50% decrease in L-valine titer.

To confirm that this frameshift mutation was ineffective, we knocked out Ptac-*ygaZ*H and Pyga*-ygaZH* individually and simultaneously. These individual knockout mutations did not result in a significant decrease in L-valine production compared to E2.5. Even when both *ygaZH* genes were knocked out simultaneously, L-valine production remained unaffected (Fig. 4b). This suggests that although the frameshift in *ygaZ* leads to the loss of function, other L-valine export proteins contribute effectively toward high production, consistent with previous studies on L-valine production optimization in *E. coli*^5^.

### Excess NADH in synthesized L-valine strains is alleviated via the L-threonine pathway

**Fig. 5:**
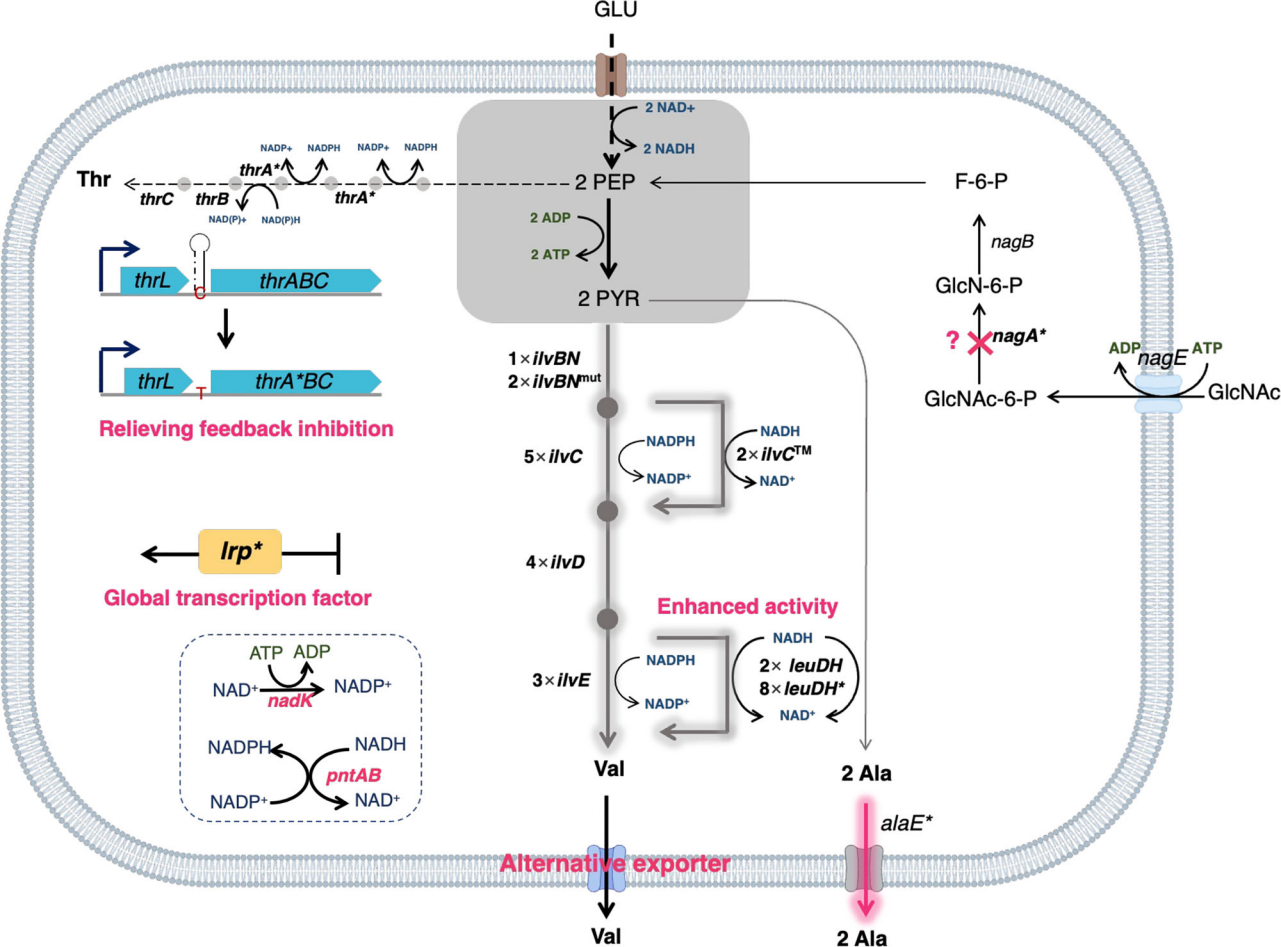
Mechanistic model underlying *lrp* (p.P139L), *ygaZ* (p.W152fs), *thrA* (upstream G-A), *thrA* (p.S354Y), and *alaE* (p.S142_Q145delinsK). A total of two NADH and two ATP molecules are generated, and two NADH molecules are consumed during the biosynthesis of one L-valine molecule from glucose under anaerobic conditions. GLU, glucose; PEP: phosphoenolpyruvate; PYR, pyruvate; Val, L-valine; Ala, L-alanine; Thr, L-threonine; GlcNAc, N-acetylglucosamine; GlcNAc-6-P, N-acetyl-D-glucosamine-6-phosphate; GlcN-6-P, glucosamine-6-phosphate; F-6-P, fructose-6-phosphate. Created with BioRender. Yang, S. (2026) https://BioRender.com/tqwgeep.

The *thrL-ABC* genes are involved in L-threonine biosynthesis, a pathway that consumes NAD(P)H (Fig. 5). The ThrL leader peptide regulates the *thrABC* operon, which encodes four of the five enzymes in the threonine biosynthesis pathway, through attenuation in response to L-threonine and L-isoleucine levels^36^ (Fig. 5).

We hypothesize that the mutation, *thrA* (upstream G-A), between *thrL* and *thrA* disrupts the attenuator structure, causing it unresponsive to threonine levels, thereby increasing *thrABC* expression. We suspect that *thrA* (p.S354Y) is a mutation that relieves threonine-mediated feedback inhibition, thereby enhancing L-threonine synthesis pathway activity. This modification, in conjunction with the mutation between *thrL and thrA*, could potentially help mitigate the excess NADH accumulation in the synthesized L-valine strains.

The introduction of *thrA* (p.S354Y) into S2.3 (S2.2::*nagA* (p.G141fs)), resulting in S2.4, increased the titers of L-threonine and L-valine. Adding *thrA* (upstream G-A) on top of S2.4 further boosted these titers (Fig. 4c), indicating that the threonine synthetic pathway, which creates an additional electronic sink, compensates for the initial excess reducing power in the reconstructed L-valine strains. We speculate that the above-mentioned *ilvA* (p.S438I) found in E2.5 contributed to increase L-threonine flux during the adaptation process. However, its contribution was rendered negligible by mutations in *thrA* (upstream G-A) and *thrA* (p.S354Y) when the production of L-valine increases along with superfluous NADH.

This raises the question of why *thrA* mutations evolved to consume excess NADH, despite the engineered L-valine pathway being redox-balanced. The likely explanation is that biomass synthesis in *E. coli* inherently produces a slight NADH surplus, even under redox-balanced conditions. In our case, although excess NADH was minimal, mutations in *thrA*, including a G → A change in the promoter region and an S354Y substitution, relieved L-threonine feedback inhibition, enabling low-level L-threonine synthesis to act as a redox sink. This represents a unique and elegant evolutionary solution to a redox challenge in a system engineered for homotypic L-valine production.

### Truncated AlaE as a gain-of-function mutation

We had thought that the *alaE* (p.S142_Q145delinsK) was a loss-of-function mutation of the L-alanine exporter. In contrast, interrupting *alaE* in E2.5 led to the L-valine production dropped sharply to below 5 g/L (Fig. 4d). Complementation with wild-type *alaE* and *alaE* (p.S142_Q145delinsK) showed that only *alaE* (p.S142_Q145delinsK) restored L-valine production, suggesting *alaE* (p.S142_Q145delinsK) is a gain-of-function mutation (Fig. 4d).

Upon reviewing the fermentation results, we observed that L-alanine accumulated around 0.2 g/L at the end of fermentation, exceeding the initial 0.1 g/L supplementation for better cell growth. Therefore, there must be other L-alanine synthesis reactions in our synthesized strains. After conducting a literature search, we found that LeuDH has 3.4% activity with L-alanine compared to L-Leucine^37^. Given that the LeuDH^V22I^ has higher activity and is present in multiple copies in S2.X strains, it is likely that LeuDH^V22I^ catalyzes pyruvate to L-alanine (Fig. 5).

To verify whether *alaE* (p.S142_Q145delinsK) enhanced the L-alanine export, we introduced this mutation into S9015 to construct S9015::*alaE*Δ, resulting in an increase in L-alanine titer from 13 to 20 g/L. This observation aligns with *alaE* (p.A48S and p.A149D) found in the evolved high-alanine-producing strains, which also likely enhanced L-alanine export. To further validate the function of *alaE* (p.A48S and p.A149D), we replaced the *alaE* (p.A48T, p.S142_Q145delinsK) with *alaE* (p.A48S and p.A149D) in E2.5 and introduced it in S2.5. The L-valine titers of E2.5::*alaE* (p.A48S and p.A149D) and S2.5::*alaE* (p.A48S and p.A149D) were even better than E2.5 and S2.6 respectively, indicating that double mutant *alaE* (p.A48S and p.A149D) exhibits functional performance similar to that of *alaE* (p.S142_Q145delinsK). Additionally, by introducing *alaE* (p.A149D) based on S2.5, we achieved L-valine production comparable to E1.0 (Fig. 4e), which showed a higher yield than E1.0 (Supplementary Data 12). These phenotypes hold the potential for further industrialization. These findings suggest that excess intracellular L-alanine compromised cellular fitness, which could be mitigated by enhancing L-alanine exportation. Importantly, *alaE* (p.A149D) was proved more effective than *alaE* (p.S142_Q145delinsK) in restoring the reduced fitness.

AlaE contains four transmembrane domains, with the last one extending to position 132 (UniProt accession number: P64550). We confirmed that C-terminal mutations beyond the last transmembrane domain (A149D and S142_Q145delinsK) enhance the L-alanine exportation. Additionally, A48 appears to be a mutation hotspot. While A48S and A48T may contribute less than C-terminal mutations—or, in some cases, have no impact on the L-valine phenotype, as seen with A48T—their frequent co-occurrence suggests they may have played a role during adaptation. However, this contribution was ultimately replaced by C-terminal mutations when the production of L-valine increased along with more L-alanine.

It may seem counterintuitive that L-alanine requires supplementation despite its accumulation during fermentation. Notably, the strain is not alanine auxotrophic; instead, it shows retarded growth in defined media lacking this additive. Thus, L-alanine was supplemented to optimize the growth kinetics of E1.0. Taken together, we propose that the C-terminal mutations of *alaE* strengthened L-alanine efflux, reducing intracellular L-alanine concentrations and improving cell fitness.

## Discussion

We achieved homotypic L-valine fermentation in *E. coli* with common metabolic engineering strategies and anaerobic adaptive laboratory evolution, which has been validated at a full industrial scale. Whole-genome resequencing of the evolved strain revealed an amino acid substitution in *leuDH* and the amplification of its variant, as well as the need to further enhance the terminal pathway genes *ilvC* and *ilvED*. Introducing these genes into the first-generation rationally designed strain, followed by anaerobic metabolic adapted evolution, resulted in improved evolution rates (Supplementary Fig. 2).

Anaerobic growth rescue has been used to develop high-yield *E. coli* strains to produce ethanol^17^, lactate^15^, succinate^38^, L-alanine^19^, and 1-butanol^13^, with commercial-scale production achieved for D-lactic acid, L-alanine, and succinate. However, none of these strains have been reconstructed to achieve genetically defined backgrounds with anaerobic growth rescue performance that is equal to or even better than the original strains. Based on the reported high-yield targets obtained through anaerobic growth rescue, most variants identified are pathway-specific to the target products. For example, a mutation in LdhL increased its activity to enhance L-lactic acid production^39^; a *glyDH* mutation enhanced LDH activity to increase D-lactic acid production^40^; a *pck* mutation which elevated its protein expression and increased succinic acid production^16^. Other targets include those enhancing cofactor supply, such as a PDH mutation that relieved NADH inhibition and increased ethanol^41^ and succinate production^42^.

Likewise, the identified targets in this study, including *ilvC*, *ilvED*, and amplified *leuDH*^V22I^, are pathway-specific, suggesting that the rate-limiting step in L-valine fermentation is related to NAD(P)H consumption. Only by converting intermediates to L-valine and thereby regenerating oxidized NAD(P)^+^ can glucose continue to be oxidized to pyruvate. The faster the target product is produced; the faster glucose consumption and cell growth occur. Consequently, strains with amplified product-synthesis genes and increased enzyme activity gain a competitive advantage under anaerobic conditions, leading to their enrichment.

Notably, during the evolution process, a variant in *lrp* emerged, underscoring the impact of global transcriptional regulators in redirecting metabolic flux (Fig. 3a). The Lrp^P139L^ variant appears only in a single pathogenic *E. coli* strain (GenBank: EKQ3327221.1), and the same mutation on its homologs could also enhance L-valine production. This effect underscores the evolutionary conservation of key regulatory residues and highlights the profound impact of minor structural changes on functional dynamics.

Unexpectedly, and in contrast to the published work^4,5,43^, we found that overexpression of *ygaZH* was unnecessary for L-valine production and could even be completely deleted (Fig. 4b). This suggests that the true exporter for L-valine remains unidentified. We also identified a gain-of-function mutation that occurred in the L-alanine exporter *alaE*, leading to the extrusion of excess L-alanine byproduct into the extracellular space, thereby enhancing intracellular cell fitness (Fig. 4d, e). From the genetic evidence, AlaE appears to exhibit the characteristics of a gain-of-function mutation, which would be more convincingly demonstrated by future biochemical validation.

The mutations in *thrA* and *thrL-thrA* revealed that cells may need an adaptation period under anaerobic conditions to fully achieve redox balance for adapting to the anaerobic environment (Fig. 4c). A key insight for accelerating similar work is whether to provide an outlet for the excess redox power. L-threonine is an ideal outlet, as its net NADH consumption prevents excessive accumulation, which could otherwise interfere with the production of the main product.

These five variants, along with the *nagA* variant and amplification of the main L-valine biosynthetic pathway genes and optimization of cofactor balance, constitute the minimal set of variants required for L-valine anaerobic production (Fig. 5).

We highlight that the improved yield from 37% (industrial-scale aerobic process at Meihua Holdings Group) to 55% in our commercial-scale anaerobic process, representing the highest yield reported to date (Table 1). This strain is currently being evaluated at 320 m^3^ scale by Meihua Group, where it has significantly reduced L-valine manufacturing costs, as glucose accounts for the largest share of total expenses. The titer of L-valine did not show further improvement, primarily due to its solubility limit in water (88.5 g/L at 25°C). Both aerobic and anaerobic fermentation processes have already reached this solubility ceiling. The production rate, however, can be improved considerably through process modifications. Our anaerobic process can achieve higher production rate from 1.37 to 1.84 g/L/h in 320 m^3^ scale by introducing oxygen in the early stage to accelerate biomass accumulation, and the fermentation cycle can also be reduced from 60 to 45 h in this condition without appreciably changing the yield (Table 1). We are exploring further production rate enhancements through cell recycling or continuous fermentation.

**Table 1:** Comparison of L-valine-producing strains

Host	Culture condition in fermenter*	Performance			Reference
		Titer	Yield	Rate	
		g/L	g/g glucose	g/L/h	
*E.coli* W	Fed-batch fermentation was carried out in a 6.6 L fermenter supplemented with 2 g/L yeast extract and 2 mM L-isoleucine.	60.7	0.22	2.06	Park et al.^6^
*E.coli* W3110	Two-stage aerobic-oxygen-limiting fed-batch fermentation was performed in a 5 L fermenter, with 5 g/L yeast extract added to the medium.	84	0.41	2.33	Hao et al.^11^
*E.coli* ATCC 8739	Anaerobic fermentation was conducted in a 320 m³ fermenter, with 0.7 g/L yeast extract, 0.6 g/L betaine hydrochloride, and 0.36 g/L L-alanine.	83.6	0.55	1.39	This study
*E.coli* ATCC 8739	Anaerobic fed-batch fermentation was performed in a 320 m³ fermenter with limited oxygen bubbling during the initial 0-4 h. The medium was supplemented with 0.7 g/L yeast extract, 0.6 g/L betaine hydrochloride, and 0.36 g/L L-alanine.	82.6	0.56	1.84	This study
*C. glutamicum*	Aerobic fed-batch fermentation was conducted in a 320m³ fermenter, supplemented with 50 g/L corn steep liquor, 2 g/L yeast extract, and 0.4 g/L soybean hydrolysate.	85	0.37	1.25	A commercial aerobic process (data provided by Meihua Holdings Group)

*We considered only these strains with available data from fermentation experiments in fermenter.

By systematically optimizing the biosynthetic pathway and introducing targeted mutations, this work sheds light on how specific genetic modifications can influence and stabilize metabolic flux under anaerobic conditions. Our study demonstrates a successful transition from aerobic to anaerobic fermentation for the production of a key amino acid, while uncovering fundamental insights into the metabolic and regulatory flexibility of *E. coli*.

## Methods

### *Escherichia coli*

We used *E. coli* ATCC 8739 as the experimental model in this study.

### Media and growth conditions

All strains were grown at 37°C, 250 rpm unless specified otherwise.

Luria–Bertani (LB) broth was used for cloning purposes. The LB medium composition is 5 g/L yeast extract, 10 g/L peptone, and 10 g/L sodium chloride. For solid media, 1.5%–2% agar powder was added. Antibiotics were added when needed at the following final concentrations: kanamycin, 50 µg/mL; spectinomycin, 100 µg/mL; chloramphenicol, 25 µg/mL; and zeocin, 50 µg/mL. L-rhamnose and L-arabinose were supplemented at a final concentration of 10 mM.

In NBS(A) and AM1(A) media, 2%–12% (w/v) glucose was added as a growth or production medium^44^. For example, with 2% glucose supplementation, the medium was referred to as NBS(A)G20 or AM1(A)G20, where G denotes glucose, 20 indicates the glucose concentration in g/L, and A denotes L-alanine. During the initial domestication, the strain was cultured in NBS(A) inorganic salt medium without antibiotics at 37°C, with 100 mM ammonium sulfate, 1 mM betaine, and 2% (w/v) glucose added. The compositions of NBS(A) and AM1(A) media are also mentioned in Supplementary Data 13.

### Strain construction

The strains used in this study are summarized in Supplementary Data 1. The initial strain S1.0 was constructed from *E. coli* ATCC 8739.

Except for the multicopy insertions of *ilvC* and *leuDH*^V22I^, all genomic modifications were carried out using a modified CRISPR-Cas9 system^45,46^ or RAGATH RNA-associated DNA endonuclease (RAD)^47^. In brief, strains transformed with pEcCas (Addgene ID #73227), pEcCas-2.0 (Addgene ID #190697), or pISFba1 (Addgene #226820) were grown overnight and then reinoculated at 1%–2% (v/v), followed by incubation to reach the OD₆₀₀ of 0.6 at 37°C in a shaker in LB medium supplemented with 10 mM L-arabinose and suitable antibiotics. After this incubation, the strains were electroporated with the modified pTargetF (Addgene ID #62226), pEcgRNA (Addgene ID #166581), or pISFba1reRNA (Addgene #226822) together with donor fragments, or the single pTargetT or pISFba1reRNAT plasmids which include donor DNA. Cells were recovered at 37°C for 1 h and plated on LB agar with suitable antibiotics for overnight growth at 37°C. Colony PCR and sequencing were used to confirm successful DNA editing. To remove the pTargetF, pTargetT, pISFba1reRNA, or pISFba1reRNAT plasmids while using pEcCas or pISFba1, cells were cultured in the LB medium with 10 mM L-rhamnose and kanamycin. The pEcCas or pISFba1 was subsequently cured by culturing the strain in LB with 10 g/L sucrose at 37°C. While using pEcCas-2.0, colonies were inoculated in the LB liquid medium and passaged to cure the plasmids.

For certain single-nucleotide mutations, a two-step CRISPR-based strategy was employed. An intermediate protospacer or antibiotic cassette (expressing apramycin) was first introduced to disrupt the original sequence, followed by a second round of editing to introduce the desired nucleotide substitution with pTargetF-Apr and certain repair templates provided in Supplementary Data 14. CRISPR-associated transposases were used for the multicopy insertion of *ilvC* and *leuDH*^V22I^ according to Yang et al.^28^. In brief, the transformation of S1.0::ilvXGMED with pQCasTns(Ptr)-frd-adhE-mgsA-ackA and pPtrDonor-ilvC was conducted, followed by *E. coli* transposition experiments. In brief, after being grown overnight at 37°C for 16 h, hundreds of colonies were scraped off from the plate. Some of them were resuspended in fresh LB medium and then re-spread on an LB-agar plate with 100 ng/mL anhydrotetracycline (aTC) and suitable antibiotics to induce protein expression. Cells were cultured at 37°C for another 16 h, and the films formed were scraped off and resuspended in LB medium. The cells were re-diluted and then spread on a plate containing 1000 ng/mL aTC and suitable antibiotics. The colonies were grown overnight at 37°C, and then colony PCR was performed for copy-number identification. Strains obtained were then treated with pFree_Zeo^48^ (Addgene ID #92053) to cure all plasmids and further transformed with pQCasTns(Ptr)-ldhA-pflB and pPtrDonor-leuDH^V22I^, followed by another round of transposition experiments. The plasmids were cured pFree_Zeo in the end.

All primers that were used in this study are listed in the Supplementary Data 15.

### Plasmid construction

All plasmids that were implemented in this study are summarized in Supplementary Data 14, with their applications in this study. Based on pQCasTns(Ptr)-entry(BsaI), CRISPR arrays for pQCasTns(Ptr)-ldhA-pflB and pQCasTns(Ptr)-frd-adhE-mgsA-ackA were synthesized by GenScript. The other plasmids were constructed by Gibson Assembly with the Hieff Clone® Universal One Step Cloning Kit (Yeason) while DNA fragments were amplified by KOD FX (Toyobo). *E. coli* DH5α and Top10 were used as the cloning hosts.

### Anaerobic growth rescues

Anaerobic growth rescues^13^ were conducted in tightly sealed 125 or 500 mL fleakers or tanks at 37°C, with pH maintained at 7.0 using 30%–50% (v/v) ammonia solution. Cultures were transferred every 48 or 24 h, with an inoculation volume of 1% (v/v) unless otherwise stated (related details are provided in Supplementary Data 2 and the Source Data file). As growth accelerated, the glucose concentration in the growth medium was gradually increased as needed.

### Anaerobic fermentations in fleakers

For L-valine, isolated single colonies of the evolved strain were streaked on plates and then inoculated into 4 mL of LB liquid medium, cultured at 37°C and 240 rpm aerobically for 24 h. A 1% (v/v) inoculum was then transferred to a sealed fleaker containing 100 or 300 mL AM1AG80 medium (unless otherwise specified) and incubated at 37°C and 120 rpm for 18–22 h. Subsequently, a 10% (v/v) inoculum was transferred to a fresh fleaker with 100 or 300 mL AM1AG80 or AM1AG100 medium unless otherwise indicated (or, alternatively, fresh medium was supplemented in the same fleaker) and cultured at 37°C and 500 rpm for 24 h. The pH was maintained at 7.0 by the addition of 30%–50% (v/v) ammonia solution.

For strain S2.0, AM1AG30 was used in the seed and production medium.

For L-alanine, AM1G120 was used as the production medium and cultured at 37°C and 500 rpm for 43 h.

### Anaerobic fermentations of L-valine-producing strains in the 3 L fermenter

Glycerol-preserved strains or single colonies were inoculated into 5 mL LB liquid medium and cultured at 37°C with shaking at 230 rpm for 24 h. A 1% (v/v) inoculum was then transferred to a sealed Erlenmeyer flask containing 100 mL of AM1AG100 medium and incubated at 37°C for 20 h, with pH maintained at 7.0 using 30%–50% (v/v) ammonia solution. Subsequently, a 15% (v/v) inoculum was transferred to a 3 L fermenter (Sartorius Stedim BIOSTAT® A Plus, 3 L total volume, 2 L working volume) containing AM1AG80 medium for fermentation at 37°C and 200 rpm under sealed, anaerobic conditions. During fermentation, pH was maintained at 7.0 using concentrated 30%–50% (v/v) solution. When the glucose concentration dropped below 20 g/L, 100 mL of 900 g/L glucose was added.

### Evaluation of L-valine-producing strains in the 10 L fermenter

Cells were scraped from the plate and inoculated into a 500 mL Erlenmeyer flask containing 60 mL of primary seed medium (Supplementary Data 13). The culture was incubated at 37°C with shaking at 100 rpm in a reciprocating shaker until OD₆₀₀ reached 4.0–5.5. Under sterile conditions, the cell suspension was then transferred to a specialized transfer cylinder.

The secondary seed fermenter had a working volume of 10 L, filled with 6 L of secondary seed medium (Supplementary Data 13). 60 mL of the primary seed culture was transferred to the secondary seed fermenter by vacuum suction from the transfer cylinder. The conditions were as follows: pressure 0.01–0.05 MPa, agitation rate 300 rpm, no aeration, and pH controlled at 6.8–7.0 with liquid ammonia. The culture was incubated anaerobically at 37°C until OD₆₀₀ reached 4.0–5.5.

The production fermenter had a working volume of 10 L, filled with 6 L of the production medium (Supplementary Data 13). 1.2 L of the primary seed culture was transferred to the 10 L production fermenter by vacuum suction from the transfer cylinder. The fermentation conditions were as follows: pressure 0.01–0.05 MPa, agitation rate 200–250 rpm, no aeration, and anaerobic incubation at 37°C for 60 h. The pH was controlled at 6.8–7.0 with liquid ammonia. When the residual glucose concentration reached ~10 g/L, a 60%–62% (w/v) glucose solution was fed at 0–50 g/h to maintain a 10 ± 2 g/L residual glucose level.

The feeding details are shown in Supplementary Data 4. Cumulative glucose feeding volume, glucose feeding rate, concentrations of L-valine and major byproducts, including the other 19 amino acids as well as acetate, citrate, malate, succinate, formate, and ethanol, were monitored throughout fermentation.

### Large-scale anaerobic fermentation (320 m³) of L-valine-producing strain

Cells were scraped from the plate and inoculated into a 5000 mL Erlenmeyer flask containing 1200 mL of primary seed medium (Supplementary Data 13). The culture was incubated at 37°C with shaking at 100 rpm in a reciprocating shaker until OD₆₀₀ reached 4.0–5.5. Under sterile conditions, the cell suspension was then transferred to a specialized transfer cylinder.

The secondary seed fermenter had a working volume of 5 m³, filled with 3 m³ of secondary seed medium (Supplementary Data 13). The primary seed culture was transferred to the secondary seed fermenter by vacuum suction from the transfer cylinder. The conditions were as follows: pressure 0.01–0.05 MPa, agitation rate 100–150 rpm, no aeration, and pH controlled at 6.8–7.0 with liquid ammonia. The culture was incubated anaerobically at 37°C until OD₆₀₀ reached 4.0–5.5.

The tertiary seed fermenter had a working volume of 60 m³, filled with 42 m³ of medium (the same as the secondary seed medium). The secondary seed culture was transferred to the tertiary fermenter by differential pressure. The conditions were as follows: pressure 0.01–0.05 MPa, agitation rate 100–150 rpm, no aeration, and pH controlled at 6.8–7.0 with liquid ammonia. The culture was incubated anaerobically at 37°C until OD₆₀₀ reached 8.0–8.5.

The main industrial fermenter had a total capacity of 320 m³ with a working volume of 200 m³ of production medium (Supplementary Data 13). The tertiary seed culture was transferred to the main industrial fermenter by differential pressure. The fermentation conditions were as follows: pressure 0.01–0.05 MPa, agitation rate 100–150 rpm, no aeration, and anaerobic incubation at 37°C for 60 h. The pH was controlled at 6.8–7.0 with liquid ammonia. When the residual glucose concentration reached ~15 g/L (9–10 h), a 60%–62% (w/v) glucose solution was fed at 0.5–1.5 m³/h to maintain a 10–15 g/L residual glucose level. Feeding was terminated at 52–54 h, and residual glucose was fully consumed at the end of fermentation (60 h).

The feeding details for Supplementary Fig. 4d are as follows: the feeding glucose concentration was 600 g/L, with a total feeding volume of 49.5 m³. The total glucose consumption was 37.05 t, and the total L-valine production was 20.48 t, corresponding to an overall yield of 0.5528 g/g.

The feeding details for Fig. 1e and Supplementary Fig. 4e are provided in Tables 2 and 3. The feeding glucose concentration was 605 g/L.

**Table 2:** Glucose feeding profile in the 320 m^3^ bioreactor (related to Fig. 1e)

Time period (h)	Duration (h)	Feeding rate (m³/h)	Feeding rate (g/h)
9–12	3	0.5	302,500
12–16	4	0.8	484,000
16–24	8	1.2	726,000
24–40	16	1.5	907,500
40–44	4	1.2	726,000
44–48	4	1.0	605,000
48–52	4	0.7	423,500
52–54	2	0.525	317,625

**Table 3:** Glucose feeding profile in the 320 m^3^ bioreactor (related to Supplementary Fig. 4e)

Time period (h)	Duration (h)	Feeding rate (m³/h)	Feeding rate (g/h)
9–11	2	0.5	302,500
11–15	4	0.8	484,000
15-16	1	1.0	605,000
16-24	8	1.2	726,000
24-40	16	1.5	907,500
40–44	4	1.2	726,000
44–48	4	1.0	605,000
48–51	3	0.7	423,500
51–53	2	0.5	317,625

### Fermentation products analysis

The biomass of *E. coli* was measured by optical density (OD) at 600 nm (OD_600_) using a UV–Vis spectrophotometer.

Glucose concentration was determined using an SBA-40C biosensor (Shandong Academy of Sciences, China) or high-performance liquid chromatography (HPLC).

The concentration of 20 amino acids was detected after derivatization with o-phthalaldehyde using HPLC with an Agilent Technologies 1200 system. The chromatographic column used was a Poroshell 120 HPH-C18 4.6 × 100 mm, 4 µm. The column temperature was set at 40°C. The mobile phase A was a solution of 10 mM Na₂HPO₄ and 10 mM NaB₄O₇ (pH 8.2). The mobile phase B consisted of a mixture of methanol, acetonitrile, and water in 45:45:10. The flow rate was 1 mL/min, and UV detection was performed at a wavelength of 338 nm.

Organic acids and ethanol were quantified by HPLC using an Agilent 1200 system equipped with a refractive index detector (RID). Separation was performed on a BIO-RAD Aminex HPX-87H column (300 × 7.8 mm). The column temperature was maintained at 30°C. The mobile phase consisted of 5 mM sulfuric acid in water, delivered at a flow rate of 0.5 mL/min. Concentrations of acetate, citrate, malate, succinate, formate, and ethanol were determined based on external standards.

### Next-generation genome sequencing

2 mL of the overnight cultures were taken to extract genomic DNA. Paired-end libraries for whole-genome sequencing were prepared using Nextera DNA Flex Library Prep Kit or TruSeq DNA PCR-Free Library Prep Kits from genomic DNA. At least 3.3 million read pairs were obtained from each sample, for a total length of 1 GB (300-fold average coverage of the genome) at least, using Novaseq PE150 systems (performed by Novogene China).

### Comparative genomics analysis

Using the genome sequence of *E. coli* ATCC 8739 (CP000946) as the reference, comparative analysis was performed using Snippy v3.1 (https://github.com/tseemann/snippy). Variants were compared with the synthesized strains, such as S1.0 and S2.0. Variants introduced during strain construction were excluded from downstream functional analysis.

The mean sequencing depth of each coding sequence (CDS) was calculated using samtools coverage. Gene copy number was inferred by dividing the mean depth of each gene by the median of the mean depths across all genes in the genome.

### RNA sequencing

Strains S2.0, S2.1, and E2.5 were collected at the exponential phase (OD_600_ ≈ 0.8) in the LB liquid medium, and ten OD units of culture were collected for further analysis. The samples were stored at −80°C until processing. Total RNA was extracted by Genewiz (China) using commercial kits according to the manufacturer’s instructions. The libraries were constructed using the TruSeq™ Stranded Total RNA Library Prep Kit, and sequencing was performed on the Illumina Novaseq 6000 platform, with a data output of 3 GB. Biomass, consumed glucose, and L-valine production were measured for the same batch of samples.

### RNA-seq analysis

Transcriptomic analysis was performed using *E. coli* ATCC 8739 genome sequence (CP000946) as the reference genome. Reads were aligned to the reference genome using HISAT2 (v2.1.0)^49^, gene-level counts were obtained using FeatureCounts (v1.6.0)^50^, and differential expression analysis was performed using DESeq2 (v1.42.0)^51^, all with default parameters.

### Protein expression and purification

The *leuDH* and its mutant were cloned into pET-28a, with a His-tag at the C-terminus of *leuDH*, and transformed into *E. coli* BL21(DE3) for expression. A single colony was inoculated into a test tube and cultured overnight at 37°C, then transferred to a 1 L flask. When the OD_600_ reached 1.0, protein expression was induced by adding 1 mM IPTG and incubating overnight at 16°C for 16 h. Cells were harvested by centrifugation (3 g, wet weight). The cell pellet was resuspended in 30 mL of lysis buffer (50 mM NaH_2_PO_4_, 300 mM NaCl, 10 mM imidazole, pH 8.0) and disrupted using a French press. The lysed cells were centrifuged at 30,000 *g* for 1 h, and the supernatant was loaded onto a Ni-NTA agarose gel column (2 mL gel slurry, 1.7 × 14 cm). The column was washed three times with 4 mL of wash buffer (50 mM NaH_2_PO_4_, 1 M NaCl, 20 mM imidazole, pH 6.5), then eluted with a buffer containing 50 mM NaH_2_PO_4_, 300 mM NaCl, 250 mM imidazole (pH 8.0) in 10 mL fractions. The fractions containing purified protein were concentrated and desalted to 0.7 mL using Amicon® Ultra Centrifugal Filters, 10 kDa MWCO^52^.

### Enzyme activity assay for L-leucine dehydrogenase

2 mL of overnight cultured cells were centrifuged at 1500 *g* for 10 min at 4°C and resuspended in 1 mL of lysis buffer (100 mM Tris–HCl, pH 7.5). The cell suspension was sonicated on ice for 2 min or disrupted using a tissue grinder with 1 mm glass beads. The NADH consumption during the reduction of ketoisoleucine by LeuDH was measured using a Tecan Spark microplate reader, monitoring absorbance changes at 340 nm. The reaction mixture (200 μL) contained ketoisoleucine (4.5 mM), NH_4_OH–NH_4_Cl (900 mM, pH 9.5), NADH (0.2 mM), and crude or purified enzyme solution (2.5 mg/L). The reaction was conducted at 30°C for 2 min, and absorbance changes at 340 nm were recorded every 10 s. One unit of LeuDH enzyme activity is defined as the amount of enzyme that catalyzes the consumption of 1 µM NADH per minute under experimental conditions. Protein concentration was determined at room temperature using the Bradford assay, with bovine serum albumin as the standard^53–55^.

### Statistics and reproducibility

All experiments were performed with at least three independent biological replicates unless otherwise indicated. Samples were assigned to experimental groups based on strain genotype or fermentation conditions. Biological replicates represent independent cultures initiated from separate colonies. Data are presented as mean ± SD (*n* = 3 biological replicates) unless otherwise stated. Statistical analyses were performed using GraphPad Prism or Python unless otherwise stated. Differences between groups were evaluated using two-sided Welch’s *t*-tests unless otherwise stated. No formal tests for normality were performed.

No statistical method was used to predetermine sample size. No data were excluded from the analyses. The experiments were not randomized because groups corresponded to predefined experimental conditions. The investigators were not blinded to allocation during experiments and outcome assessment.

### Reporting summary

Further information on research design is available in the Nature Portfolio Reporting Summary linked to this article.

## Supplementary information

Supplementary Information

Description of Additional Supplementary Files

Supplementary Data 1

Supplementary Data 2

Supplementary Data 3

Supplementary Data 4

Supplementary Data 5

Supplementary Data 6

Supplementary Data 7

Supplementary Data 8

Supplementary Data 9

Supplementary Data 10

Supplementary Data 11

Supplementary Data 12

Supplementary Data 13

Supplementary Data 14

Supplementary Data 15

Reporting Summary

Transparent Peer Review file

## Source data

Source Data

## Data Availability

Summary data for strains, media, plasmids, and oligonucleotides used in this study can be found in Supplementary Data 1, 13, 14, and 15. The annotated DNA plasmid sequences and linear DNA sequences listed in Supplementary Data 14 are available at 10.5281/zenodo.19598342. Raw data for genome and transcriptome sequencing have been deposited in the NCBI database under BioProject accession PRJNA1190152. The anaerobic adaptive laboratory evolution data are provided in Supplementary Data 2. Supplementary Data 3 contains the calculations of glucose consumptions and L-valine yields for strain E1.0 in the 320 m³ fermenter. Supplementary Data 4 provides the redox and carbon balance reconciliation of strain E1.0 in 10 L fermenters. Transcriptome sequencing analyses are provided in Supplementary Data 5, 6, 10, and 11. Genome sequencing analyses are provided in Supplementary Data 7–9. Supplementary Data 12 shows the comparison between strains E1.0 and S2.5 *alaE* (p.A149D). Source data are provided with this paper.
