# Supplementary material for: Anaerobic metabolic evolution for homotypic L-valine fermentation
Source: Nat Commun. 2026 May 29;17:6996. doi: 10.1038/s41467-026-73619-7 (PMC13392391; doi:10.1038/s41467-026-73619-7)
Supplement: Supplementary file 1 — Supplementary Information [file 41467_2026_73619_MOESM1_ESM.pdf]

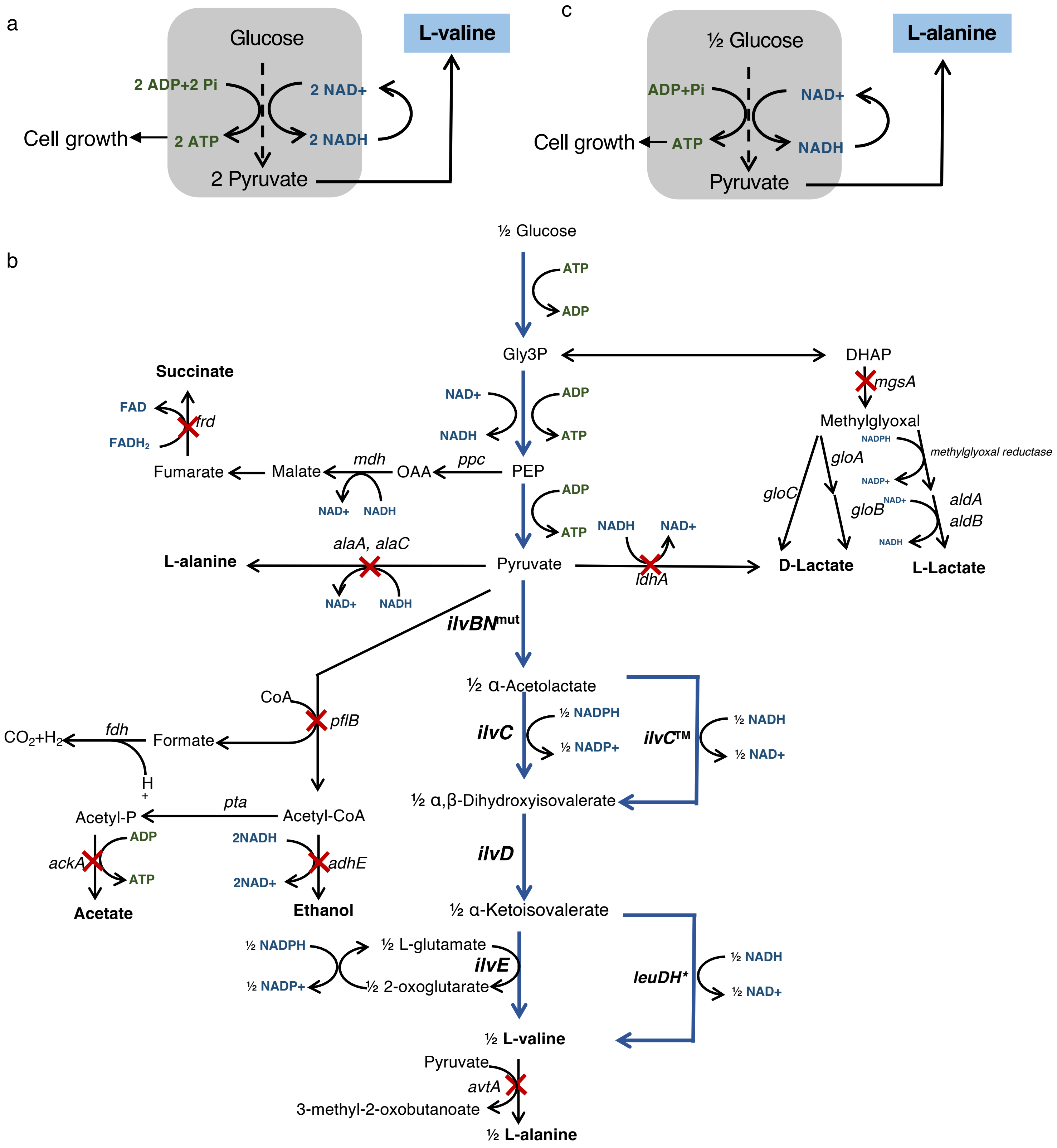

Supplementary Figure 1

Schematic diagrams of anaerobic L-valine and L-alanine production.

a. Schematic diagram of anaerobic L-valine fermentation in *E. coli*. A total of two NADH and two ATP molecules are generated, and two NADH molecules are consumed during the biosynthesis of one molecule of L-valine from glucose under anaerobic conditions.

b. Schematic overview of the systems metabolic engineering strategy and its key components for anaerobic L-valine production in *E. coli*. Blue arrows indicate L-valine pathway, while red Xs denote removed inhibitory or repressive elements (*avtA, ldhA, mgsA, frd, pflB, adhE,* and *ackA*). Asterisks indicate mutant variants.

c. Schematic diagram of anaerobic L-alanine fermentation in *E. coli*.

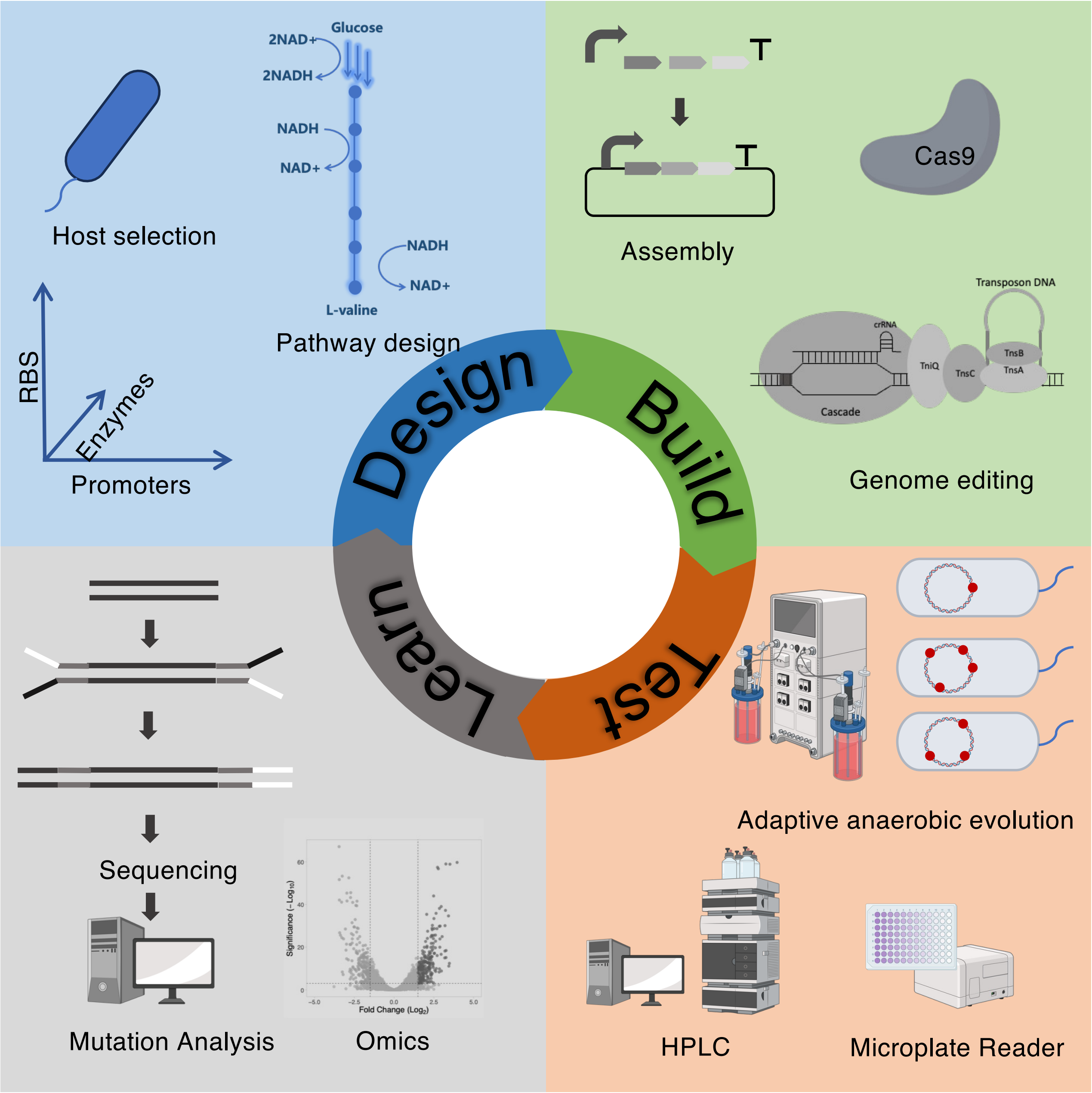

Supplementary Figure 2

Schematic of strategies and insights for rational design of an anaerobic L-valine-producing cell factory.

Design: The commonly used anaerobic *E. coli* strain ATCC 8739 was selected and engineered for anaerobic L-valine production with a focus on redox balance. Hheterologous enzymes, including LeuDH, $IlvC_{Cg}$, were introduced to construct the strain S1.0.

Build: Strain was primarily constructed using CRISPR-Cas9 and CRISPR-associated transposase.

Test: Adaptive evolution was performed in an anaerobic chemostat, and L-valine production was quantified by HPLC. The activities of LeuDH and its variants were measured using a microplate reader.

Learn: Omics analyses, particularly whole-genome sequencing, were performed to identify key mutations and guide further strain optimization.

Created with BioRender. Yang, S. (2026) https://BioRender.com/kf7n9n7.

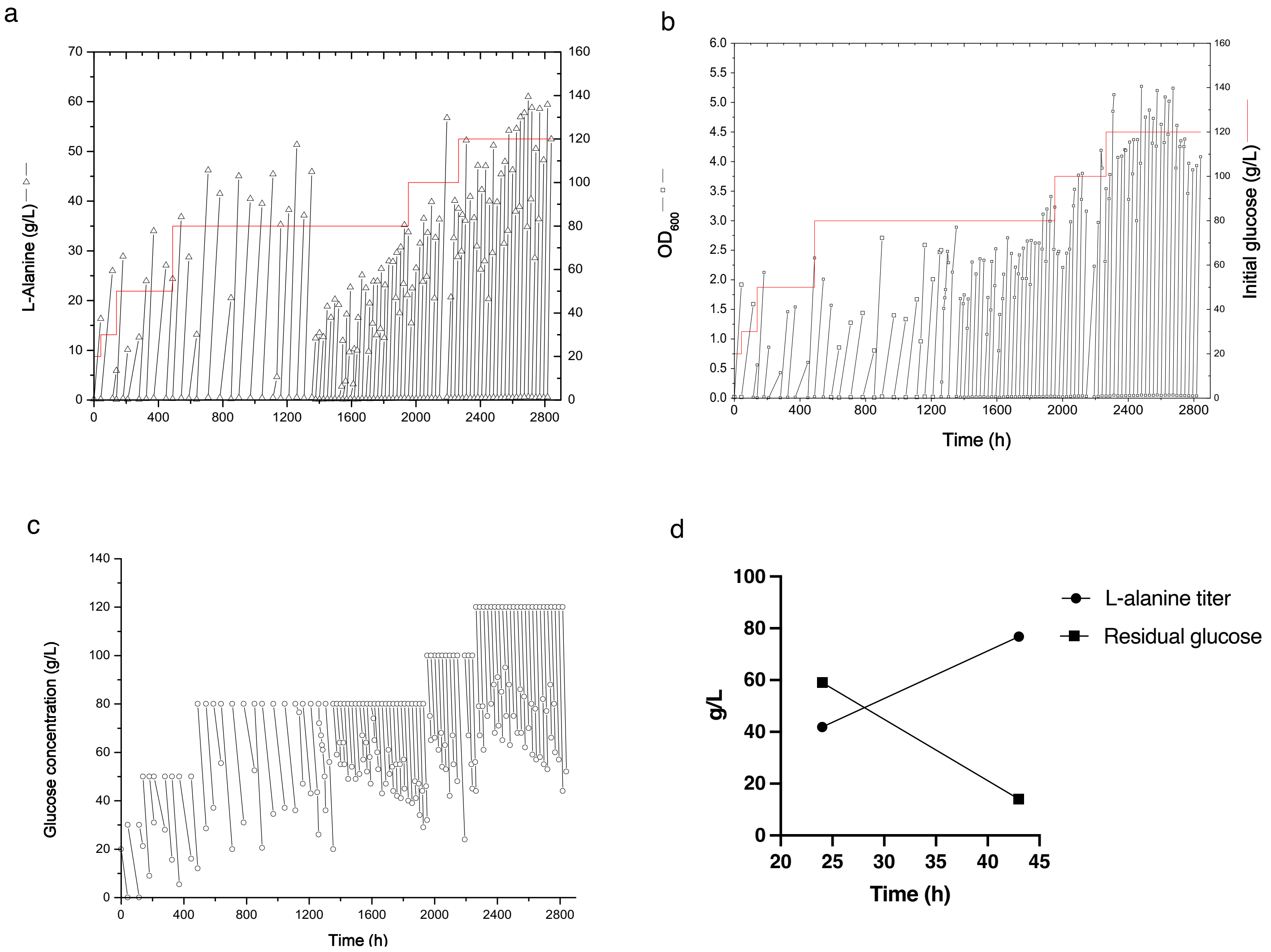

Supplementary Figure 3

A highly productive L-alanine-producing strain derived from anaerobic adaptive evolution.

a. Schematic illustrating the construction and evolutionary trajectory of the anaerobic L-alanine hyperproducing strain.

b. Growth curve of strain S9015 during adaptive evolution under increasing glucose concentrations.

c. Glucose consumption profile of strain S9015 throughout the evolutionary process.

d. Anaerobic fermentation performance of strain E8482 in a fleaker.

Source data for this figure is available in the Source Data file.

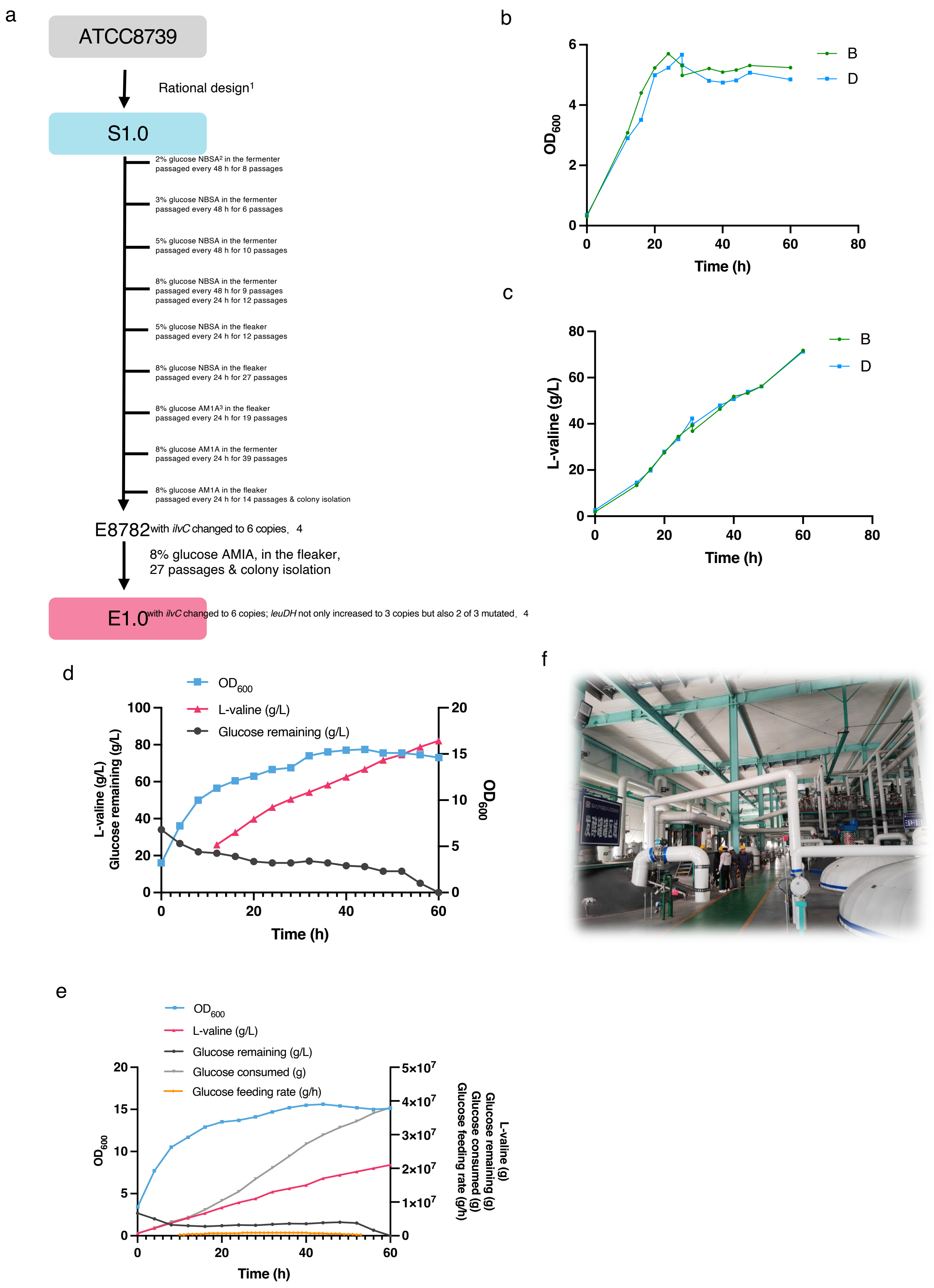
a
ATCC8739
Rational design1
S1.0
2% glucose NBSA2 in the fermenter passaged every 48 h for 8 passages
3% glucose NBSA in the fermenter passaged every 48 h for 6 passages
5% glucose NBSA in the fermenter passaged every 48 h for 10 passages
8% glucose NBSA in the fermenter passaged every 48 h for 9 passages passaged every 24 h for 12 passages
5% glucose NBSA in the fleaker passaged every 24 h for 12 passages
8% glucose NBSA in the fleaker passaged every 24 h for 27 passages
8% glucose AM1A3 in the fleaker passaged every 24 h for 19 passages
8% glucose AM1A in the fermenter passaged every 24 h for 39 passages
8% glucose AM1A in the fleaker passaged every 24 h for 14 passages & colony isolation
E8782 with ilvC changed to 6 copies, 4
8% glucose AMIA, in the fleaker, 27 passages & colony isolation
E1.0 with ilvC changed to 6 copies; leuDH not only increased to 3 copies but also 2 of 3 mutated, 4
b
OD600
Time (h)
B
D
c
L-valine (g/L)
Time (h)
B
D
d
OD600
L-valine (g/L)
Glucose remaining (g/L)
L-valine (g/L) Glucose remaining (g/L)
OD600
Time (h)
e
OD600
L-valine (g/L)
Glucose remaining (g/L)
Glucose consumed (g)
Glucose feeding rate (g/h)
OD600
L-valine (g) Glucose remaining (g) Glucose consumed (g) Glucose feeding rate (g/h)
Time (h)
f

g

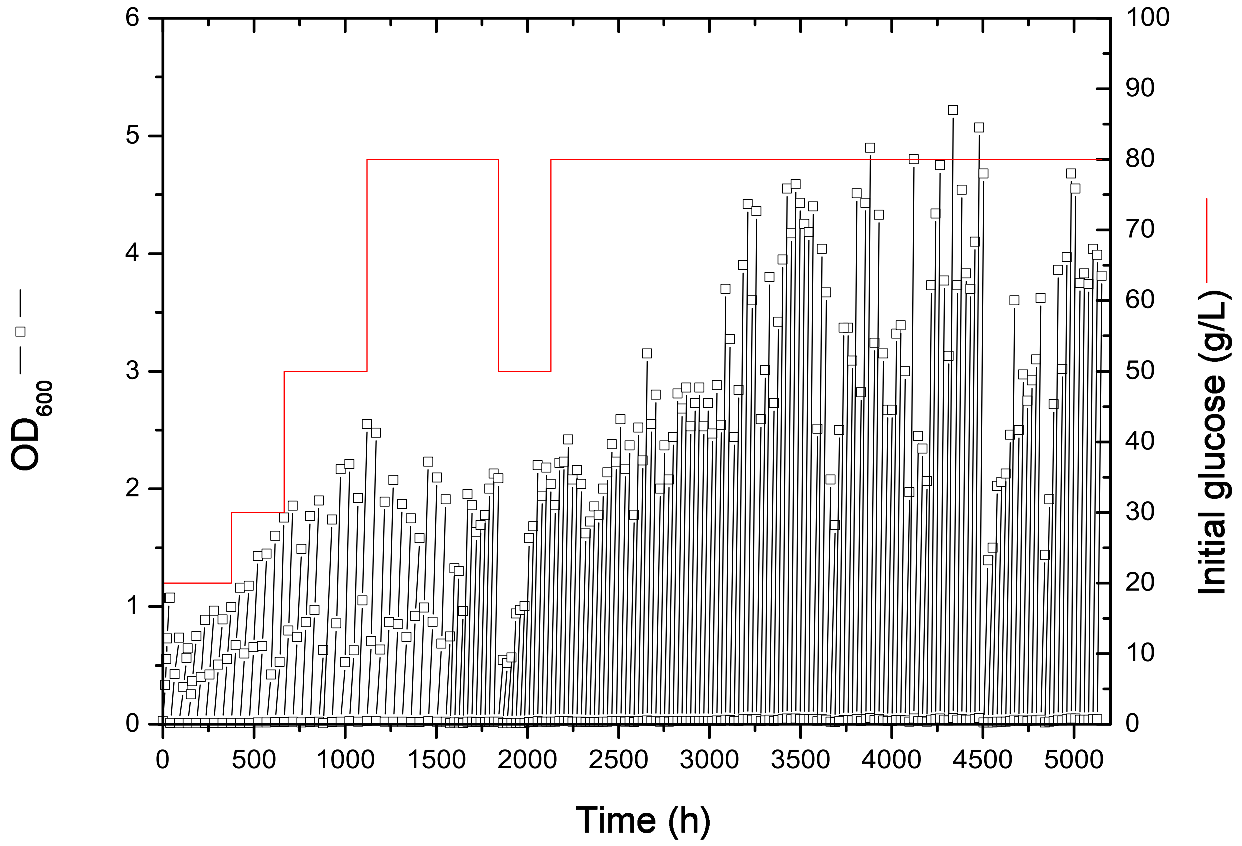
OD600
Initial glucose (g/L)
Time (h)

h

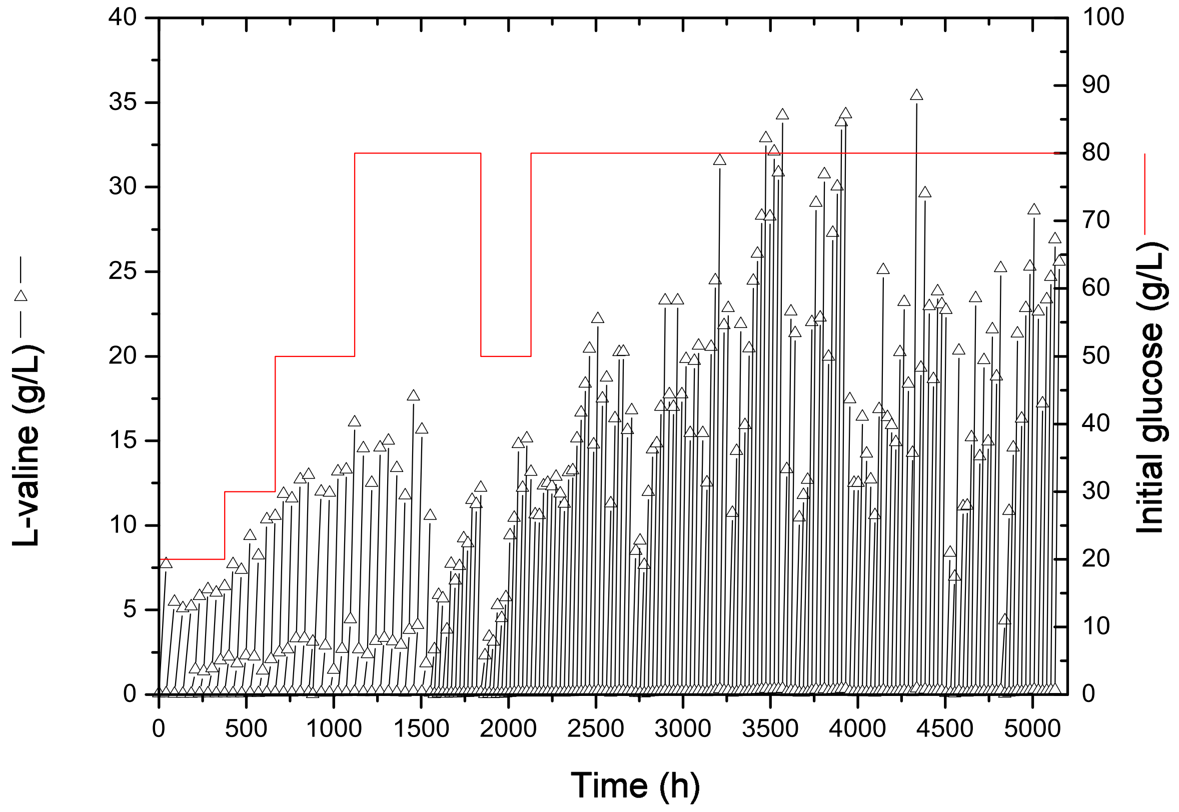
L-valine (g/L)
Initial glucose (g/L)
Time (h)

Supplementary Figure 4
Evolution of S1.0 and Fermentation of E1.0.
a. Detailed flowchart for the construction and evolution of the anaerobic L-valine hyperproducer strain, related to Figure 1A.
1. The following mutations were introduced: △*avtA*::Ptac-*leuDH*, △*ldhA*::Ptac-*ilvC*^{TM}, △*mgsA*::Ptac-*lrp*, △*frd*::Ptac-*ygaZH*, △*pflB*::Ptac-*leuDH*, △*adhE*::Ptac-*ilvBN*^{mut}△*ackA*::Ptac-*ilvED*, △*alaA*, △*alaC*, △*lacZ*::PldhA- *ilvC*^{TM}, △*lacI*::PldhA-*leuDH*, △*ydik*::PldhA-*ilvD*, △*yaiT*::PldhA-*nadK*, △*yihF*::PldhA-*ilvBN*^{mut}, △*yjcS*::Ptac-*ilvC*, △*ybaP*::Ptac-*pntAB*.
2. NBSA refers to the NBS mineral salts medium, supplemented with 100 mM $(NH_4)_2SO_4$, 1 mM betaine HCl, and 0.1 g/L L-alanine.
3. AM1A is a low-salt version of AM1 medium, supplemented with 1 mM betaine HCl and 0.1 g/L L-alanine.
4. All transfers were performed using a 1% (v/v) inoculum unless otherwise stated in Supplementary Data 2.
5. The detailed genotypes are provided in Supplementary Data 1.
(b) and **(c)** Two parallel experiments were performed under anaerobic conditions using AMIAG80 as the initial medium and a 15%(v/v) inoculum. After 28 h, 100 mL of 900 g/L glucose was fed.
b. Optical density (OD) profiles of E1.0 during fermentation in 3 L bioreactors.
c. Time course of L-valine production by E1.0 in 3 L bioreactors.
d. Anaerobic fermentation performance of E1.0 in a 320 m^3 industrial fermenter (batch 1). When the residual glucose concentration reached ~15 g/L (9–10 h), a 60–62% (w/v) glucose solution was fed at 0.5–1.5 m^3/h to maintain 10–15 g/L residual glucose. Feeding was terminated at 52–54 h, and residual glucose was fully consumed before the end of fermentation (60 h). For this batch, the feeding glucose concentration was 600 g/L, with a total feeding volume of 49.5 m^3. The total glucose consumption was 37.05 t, the total valine production was 20.48 t, corresponding to an overall yield of 0.5528 g/g.
e. Anaerobic fermentation performance of E1.0 in a 320 m^3 industrial fermenter (batch 3) under similar feeding conditions with batch 1. The detailed feeding profile is shown in Table 3.
f. Anaerobic fermentation in a large-scale industrial bioreactor.
g. Magnified view of Figure 1c.
h. Magnified view of Figure 1d.
Source data for this figure is available in the Source Data file.

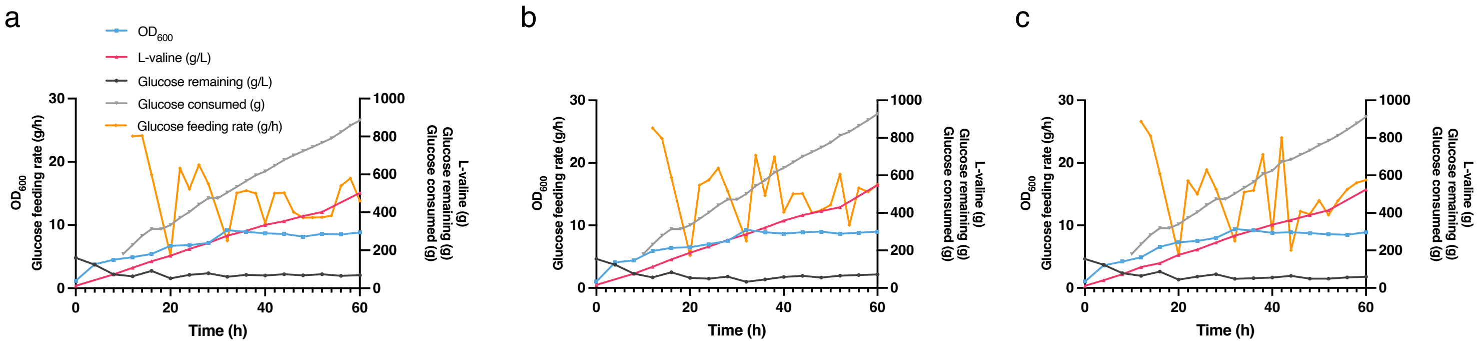

Supplementary Figure 5

Fed-batch fermentation of E1.0 in 10-L fermenters.

a Fed-batch fermentation performance of E1.0 in a 10-L fermenter (batch 1). **b** Fed-batch fermentation performance of E1.0 in a 10-L fermenter (batch 2). **c** Fed-batch fermentation performance of E1.0 in a 10-L fermenter (batch 3).

When the residual glucose concentration reached ~10 g/L, a 60–62% (w/v) glucose solution was fed at 0-50 g/h to maintain a 10±2 g/L residual glucose level.

When the residual glucose concentration reached ~15 g/L (9–10 h), a 60–62% (w/v) glucose solution was fed at 0.5–1.5 m^3/h to maintain 10–15 g/L residual glucose. Feeding was terminated at 60 h.

Source data for this figure is available in the Source Data file.

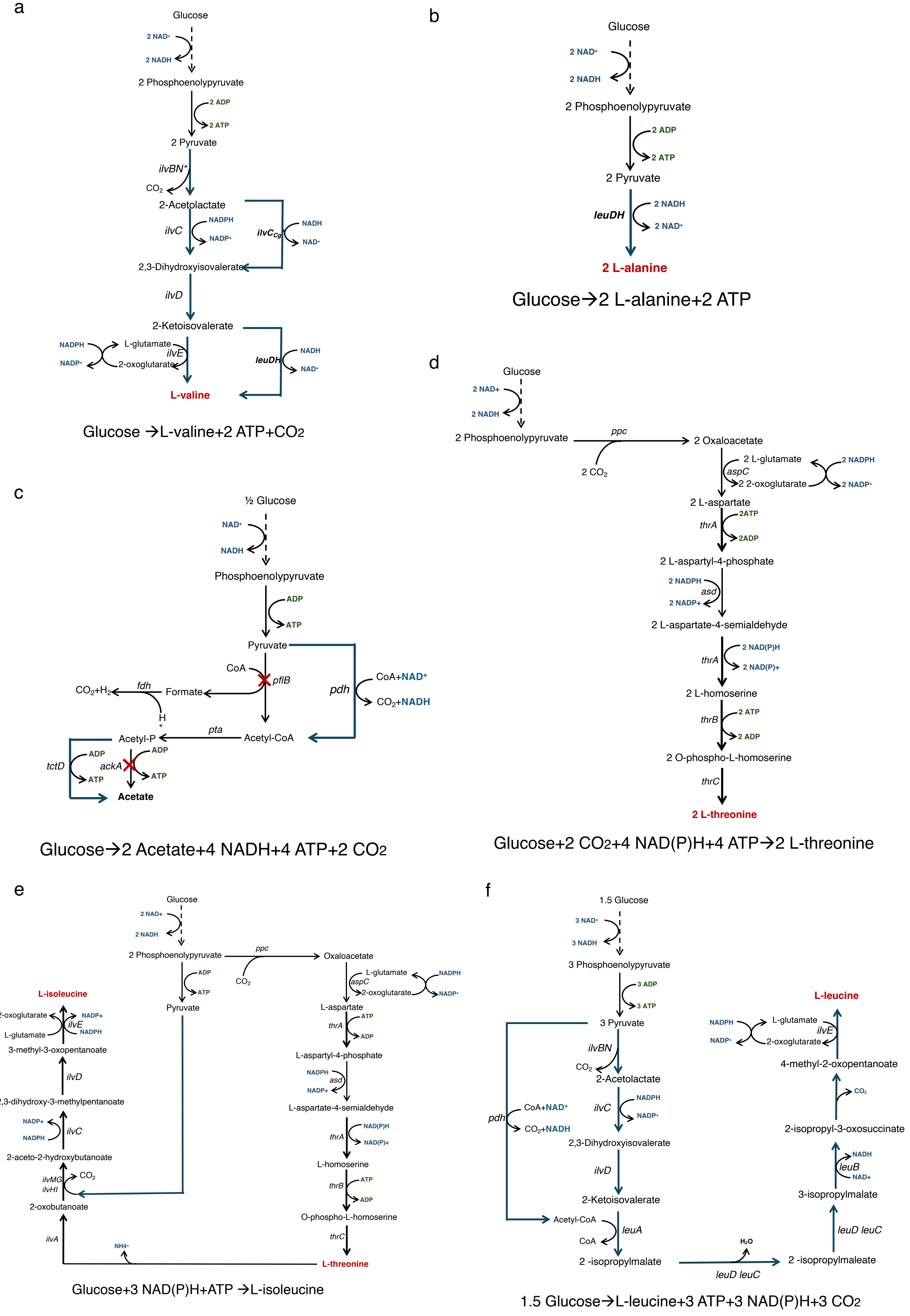
a
Glucose
2 NAD+
2 NADH
2 Phosphoenolypyruvate
2 ADP
2 ATP
2 Pyruvate
ilvBN*
CO2
2-Acetolactate
ilvC
NADPH
NADP+
ilvCCg
NADH
NAD+
2,3-Dihydroxyisovalerate
ilvD
2-Ketoisovalerate
NADPH
NADP+
L-glutamate
2-oxoglutarate
ilvE
leuDH
NADH
NAD+
L-valine
Glucose →L-valine+2 ATP+CO2
b
Glucose
2 NAD+
2 NADH
2 Phosphoenolypyruvate
2 ADP
2 ATP
2 Pyruvate
leuDH
2 NADH
2 NAD+
2 L-alanine
Glucose→2 L-alanine+2 ATP
c
½ Glucose
NAD+
NADH
Phosphoenolypyruvate
ADP
ATP
Pyruvate
CoA
pflB
pdh
CoA+NAD+
CO2+NADH
CO2+H2
fdh
Formate
H+
Acetyl-P
pta
Acetyl-CoA
tctD
ADP
ATP
ackA
ADP
ATP
Acetate
Glucose→2 Acetate+4 NADH+4 ATP+2 CO2
d
Glucose
2 NAD+
2 NADH
2 Phosphoenolypyruvate
ppc
2 CO2
2 Oxaloacetate
2 L-glutamate
aspC
2 2-oxoglutarate
2 NADPH
2 NADP+
2 L-aspartate
thrA
2ATP
2ADP
2 L-aspartyl-4-phosphate
2 NADPH
asd
2 NADP+
2 L-aspartate-4-semialdehyde
thrA
2 NAD(P)H
2 NAD(P)+
2 L-homoserine
thrB
2 ATP
2 ADP
2 O-phospho-L-homoserine
thrC
2 L-threonine
Glucose+2 CO2+4 NAD(P)H+4 ATP→2 L-threonine
e
Glucose
2 NAD+
2 NADH
2 Phosphoenolypyruvate
ADP
ATP
Pyruvate
ppc
CO2
Oxaloacetate
L-glutamate
aspC
2-oxoglutarate
NADPH
NADP+
L-aspartate
thrA
ATP
ADP
L-aspartyl-4-phosphate
NADPH
asd
NADP+
L-aspartate-4-semialdehyde
thrA
NAD(P)H
NAD(P)+
L-homoserine
thrB
ATP
ADP
O-phospho-L-homoserine
thrC
L-threonine
L-isoleucine
2-oxoglutarate
L-glutamate
ilvE
NADP+
NADPH
3-methyl-3-oxopentanoate
ilvD
2,3-dihydroxy-3-methylpentanoate
NADP+
NADPH
ilvC
2-aceto-2-hydroxybutanoate
ilvMG
ilvHI
CO2
2-oxobutanoate
ilvA
NH4+
Glucose+3 NAD(P)H+ATP →L-isoleucine
f
1.5 Glucose
3 NAD+
3 NADH
3 Phosphoenolypyruvate
3 ADP
3 ATP
3 Pyruvate
ilvBN
CO2
2-Acetolactate
ilvC
NADPH
NADP+
2,3-Dihydroxyisovalerate
ilvD
2-Ketoisovalerate
pdh
CoA+NAD+
CO2+NADH
Acetyl-CoA
CoA
leuA
2 -isopropylmalate
H2O
leuD leuC
2 -isopropylmaleate
leuD leuC
3-isopropylmalate
leuB
NADH
NAD+
2-isopropyl-3-oxosuccinate
CO2
4-methyl-2-oxopentanoate
NADPH
NADP+
L-glutamate
2-oxoglutarate
ilvE
L-leucine
1.5 Glucose→L-leucine+3 ATP+3 NAD(P)H+3 CO2

Supplementary Figure 6

Stoichiometric frameworks for redox and carbon balance of major fermentation products.

a. Stoichiometric route for L-valine biosynthesis.
b. Stoichiometric route for L-alanine formation.
c. Stoichiometric route for acetate formation.
d. Stoichiometric route for L-threonine biosynthesis.
e. Stoichiometric route for L-isoleucine biosynthesis.
f. Stoichiometric route for L-leucine biosynthesis.

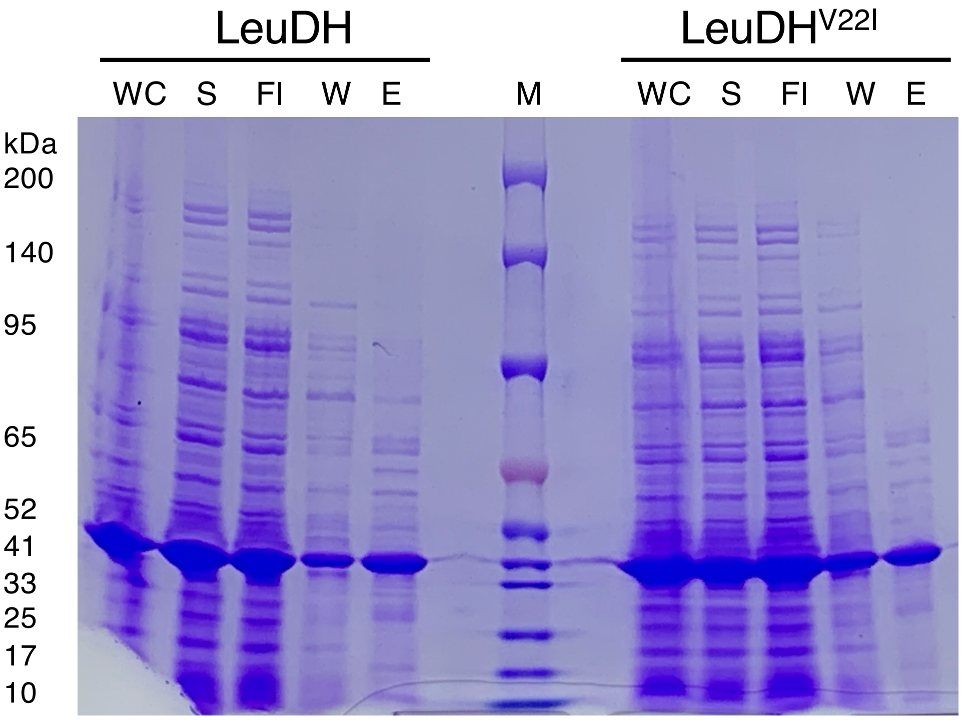

Supplementary Figure 7
SDS-PAGE analysis of LeuDH and LeuDH^{V22I}.
WC: whole cell; S: supernatant; FL: Flow-through; WB: wash buffer; EB: elution buffer. Size of LeuDH and LeuDH^{V22I}: 38.9 kDa.
Source data for this figure is available in the Source Data file.

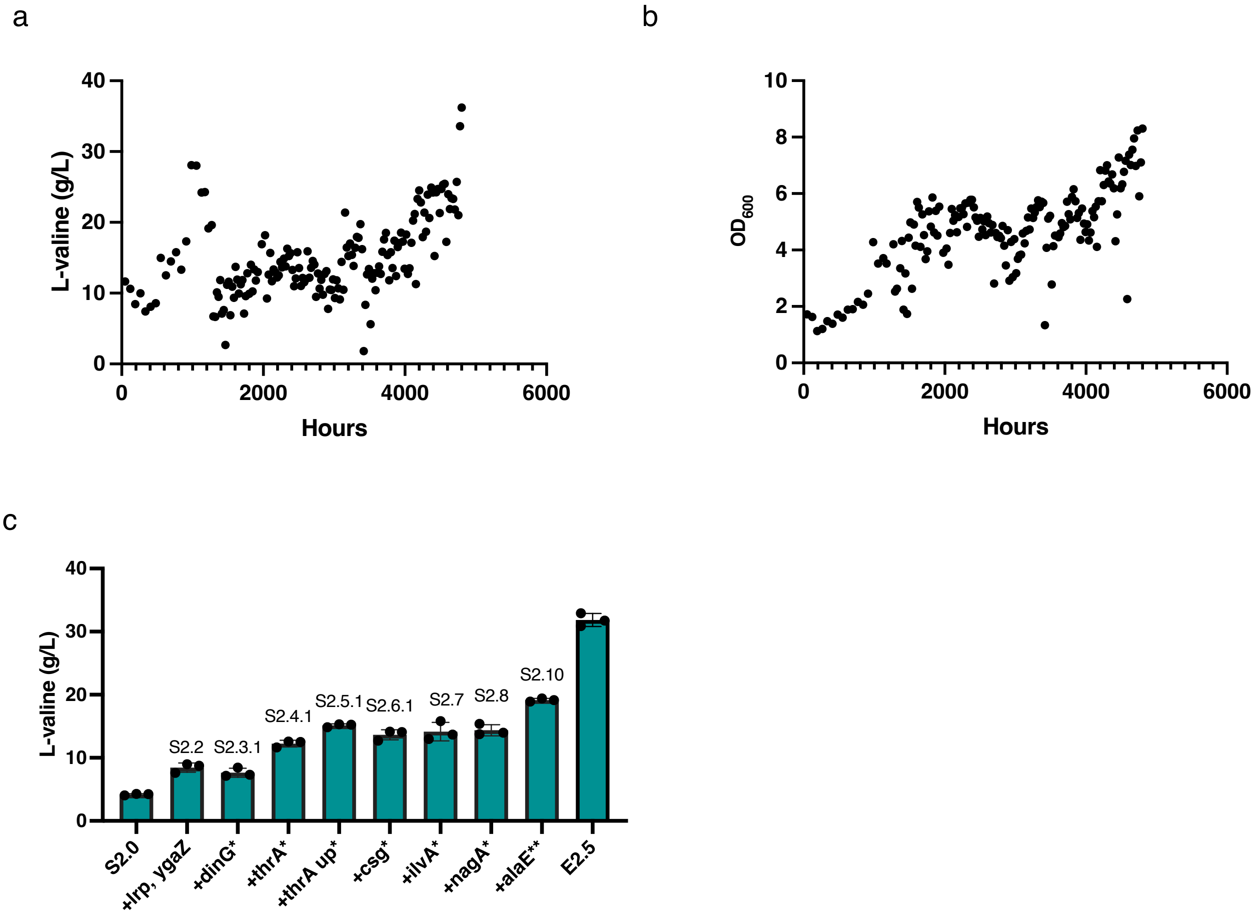

Supplementary Figure 8
Evolution and Optimization of L-valine-Producing Strains.

a. Evolutionary trajectory of S2.0 during serial passaging.

b. Growth curve of strain S2.0 throughout evolution with increasing glucose concentrations.

c. L-valine production of combining 10 mutations. Data are shown as mean ± SD (n=3 biological replicates), where each replicate represents an independently grown culture. Statistical significance was assessed using a two-sided Welch's t-test. Source data, more results of pairwise comparisons, including P values, t statistics, degrees of freedom (df), and 95% confidence intervals, are provided in the Source Data file.
