# Supplementary material for: Anaerobic metabolic evolution for homotypic L-valine fermentation
Source: Nat Commun. 2026 May 29;17:6996. doi: 10.1038/s41467-026-73619-7 (PMC13392391; doi:10.1038/s41467-026-73619-7)
Supplement: Supplementary file 2 — Description of Additional Supplementary Files [file 41467_2026_73619_MOESM2_ESM.pdf]

Description of Additional Supplementary Files

Supplementary Data 1. Description of the strains used in this study.

Excel spreadsheet listing strain names, genotypes, and corresponding evolution conditions.

Supplementary Data 2. Adaptive Evolution Progress Table for strain E1.0.

Excel spreadsheet documenting the adaptive laboratory evolution process of strain E1.0, including passage number, cultivation time, and corresponding measurements.

Supplementary Data 3. Calculations of sugar consumption and L-valine yield for strain E1.0 in the 320 m^3 fermenter.

Excel spreadsheet containing the detailed calculations of sugar consumption, residual sugar, L-valine production, and yield values for strain E1.0 during fermentation in the 320 m^3 fermenter across three batches.

Supplementary Data 4. Redox and carbon balance reconciliation of strain E1.0 in 10 L bioreactors.

Excel spreadsheet containing the detailed calculations of redox balance and carbon reconciliation for strain E1.0 during fermentation in 10 L bioreactors.

Supplementary Data 5. Transcriptome analysis of strains E1.0 and S1.0.

Excel spreadsheet containing the RNA-seq differential expression analysis results for strains E1.0 and S1.0, including gene IDs, $\log_2$ fold changes, and adjusted P values. Differential expression analysis was performed using DESeq2, which models count data using a negative binomial distribution. Statistical significance was assessed using the two-sided Wald test, and P values were adjusted.

Supplementary Data 6. Transcriptome analysis of key redox-related and L-valine biosynthetic genes.

Excel spreadsheet containing RNA-seq expression data for key genes involved in redox metabolism and L-valine biosynthesis. Statistical significance was assessed using the two-sided Fisher test, and P values were adjusted using the Benjamini–Hochberg method.

Supplementary Data 7. Single nucleotide variations in evolved strains identified by whole-genome sequencing.

Excel spreadsheet listing single nucleotide variations identified by whole-genome sequencing, including genomic positions and affected genes.

Supplementary Data 8. Copy number variations in evolved strains identified by whole-genome sequencing.

Excel spreadsheet listing copy number variations identified by whole-genome sequencing, including genomic positions and copy numbers.

Supplementary Data 9. Single nucleotide variations in E2.5 compared with S2.0 excluding synonymous mutations.

Excel spreadsheet listing the single nucleotide variations between E2.5 and S2.0 identified by whole-genome sequencing.

Supplementary Data 10. Transcriptome analysis of strains S2.1 and S2.0.

Excel spreadsheet containing the RNA-seq differential expression analysis results for strains S2.1 and S2.0, including gene IDs, $\log_2$ fold changes, and adjusted P values. Differential expression analysis was performed using DESeq2, which models count data using a negative binomial distribution. Statistical significance was assessed using the two-sided Wald test, and P values were adjusted.

Supplementary Data 11. Transcriptome analysis of strains E2.5 and S2.0.

Excel spreadsheet containing the RNA-seq differential expression analysis results for strains E2.5 and S2.0, including gene IDs, $\log_2$ fold changes, and adjusted P values. Differential expression analysis was performed using DESeq2, which models count data using a negative binomial distribution. Statistical significance was assessed using the two-sided Wald test, and P values were adjusted.

Supplementary Data 12. Comparison between strains E1.0 and S2.5 *alaE* (p. A149D).

Excel spreadsheet listing OD_{600}, titer, rate and yield of strains E1.0 and S2.5 *alaE* (p. A149D).

Supplementary Data 13 Compositions of fermentation media.

Excel spreadsheet listing the components and concentrations of the fermentation media used in the experiments.

Supplementary Data 14. Plasmids used in this study.

Excel spreadsheet describing plasmid names, DNA sequences, and deposition information

Supplementary Data 15. Primers used for strain construction and verification.

Excel spreadsheet containing primer sequences and their applications.
