# Supplementary material for: Anaerobic metabolic evolution for homotypic L-valine fermentation
Source: Nat Commun. 2026 May 29;17:6996. doi: 10.1038/s41467-026-73619-7 (PMC13392391; doi:10.1038/s41467-026-73619-7)
Supplement: Supplementary file 18 — Reporting Summary [file 41467_2026_73619_MOESM18_ESM.pdf]

Corresponding author(s): Yu Jiang and Sheng Yang

Last updated by author(s): Apr 14, 2026

Reporting Summary

Nature Portfolio wishes to improve the reproducibility of the work that we publish. This form provides structure for consistency and transparency in reporting. For further information on Nature Portfolio policies, see our Editorial Policies and the Editorial Policy Checklist.

Statistics

For all statistical analyses, confirm that the following items are present in the figure legend, table legend, main text, or Methods section.

n/a	Confirmed	
☐	☒	The exact sample size (n) for each experimental group/condition, given as a discrete number and unit of measurement
☐	☒	A statement on whether measurements were taken from distinct samples or whether the same sample was measured repeatedly
☐	☒	The statistical test(s) used AND whether they are one- or two-sided<br>*Only common tests should be described solely by name; describe more complex techniques in the Methods section.*
☒	☐	A description of all covariates tested
☐	☒	A description of any assumptions or corrections, such as tests of normality and adjustment for multiple comparisons
☐	☒	A full description of the statistical parameters including central tendency (e.g. means) or other basic estimates (e.g. regression coefficient) AND variation (e.g. standard deviation) or associated estimates of uncertainty (e.g. confidence intervals)
☐	☒	For null hypothesis testing, the test statistic (e.g. F, t, r) with confidence intervals, effect sizes, degrees of freedom and P value noted<br>*Give P values as exact values whenever suitable.*
☒	☐	For Bayesian analysis, information on the choice of priors and Markov chain Monte Carlo settings
☒	☐	For hierarchical and complex designs, identification of the appropriate level for tests and full reporting of outcomes
☒	☐	Estimates of effect sizes (e.g. Cohen's d, Pearson's r), indicating how they were calculated

Our web collection on statistics for biologists contains articles on many of the points above.

Software and code

Policy information about availability of computer code

Data collection	HPLC data were collected using Agilent ChemStation (Version B.04.02) software.
Data analysis	HPLC data were analyzed using Agilent ChemStation (Version B.04.02) software. Microsoft Excel was used to analyze concentration data. Graphpad Prism and https://www4.shbiochip.com/SBCToolBox/ was used to generate plots and calculate standard deviations. Using the genome sequence of E. coli ATCC 8739 (CP000946) as the reference, comparative analysis was performed using Snippy v3.1 (https://github.com/tseemann/snippy). The mean sequencing depth of each coding sequence (CDS) was calculated using samtools coverage. Gene copy number was inferred by dividing the mean depth of each gene by the median of the mean depths across all genes in the genome. Transcriptomic analysis was performed using E. coli ATCC 8739 genome sequence (CP000946) as the reference genome. Reads were aligned to the reference genome using HISAT2 (v2.1.0), gene-level counts were obtained using FeatureCounts (v1.6.0), and differential expression analysis was performed using DESeq2 (v1.42.0), all with default parameters.

For manuscripts utilizing custom algorithms or software that are central to the research but not yet described in published literature, software must be made available to editors and reviewers. We strongly encourage code deposition in a community repository (e.g. GitHub). See the Nature Portfolio guidelines for submitting code & software for further information.

Data

Policy information about availability of data

All manuscripts must include a data availability statement. This statement should provide the following information, where applicable:

- Accession codes, unique identifiers, or web links for publicly available datasets
- A description of any restrictions on data availability
- For clinical datasets or third party data, please ensure that the statement adheres to our policy

Summary data for strains, media, plasmids, and oligonucleotides used in this study can be found in Supplementary Tables 1, 13, 14 and 15. The annotated DNA plasmid sequences and linear DNA sequences listed in Supplementary Table 15 are available at https://doi.org/10.5281/zenodo.19598342. Raw data for genome and transcriptome sequencing have been deposited in the NCBI database under Bioproject accession PRJNA1190152 (https://www.ncbi.nlm.nih.gov/bioproject/PRJNA1190152/). The anaerobic adaptive laboratory evolution data are provided in Supplementary Table 2. Supplementary Table 3 contains the calculations of glucose consumptions and L-valine yields for strain E1.0 in the 320 m^3 fermenter. Supplementary Table 4 provides the redox and carbon balance reconciliation of strain E1.0 in 10 L fermenters. Transcriptome sequencing analyses are provided in Supplementary Tables 5,6, 10 and 11. Genome sequencing analyses are provided in Supplementary Tables 7-9. Supplementary Table 12 shows the comparison between strains E1.0 and S2.5 alaE (p. A149D). Source data are provided with this paper.

Research involving human participants, their data, or biological material

Policy information about studies with human participants or human data. See also policy information about sex, gender (identity/presentation), and sexual orientation and race, ethnicity and racism.

Reporting on sex and gender	N/A
Reporting on race, ethnicity, or other socially relevant groupings	N/A
Population characteristics	N/A
Recruitment	N/A
Ethics oversight	N/A

Note that full information on the approval of the study protocol must also be provided in the manuscript.

Field-specific reporting

Please select the one below that is the best fit for your research. If you are not sure, read the appropriate sections before making your selection.

☒ Life sciences ☐ Behavioural & social sciences ☐ Ecological, evolutionary & environmental sciences

For a reference copy of the document with all sections, see nature.com/documents/nr-reporting-summary-flat.pdf

Life sciences study design

All studies must disclose on these points even when the disclosure is negative.

Sample size	Sample sizes were selected based on acceptable convention in the field where 3 replicates is standard practice for HPLC analysis of metabolites produced via fermentation. No statistical methods were used to predetermine sample size.
Data exclusions	No data were excluded from the analyses.
Replication	Fermentations for key strains in the fleakers were performed in at least three independent runs on separate days, with consistent production trends and growth profiles observed across all experiments. Large-scale fermentations were repeated three times. All replicates are reported as plots in figures or shown in different figures.
Randomization	E.coli colonies from transformations were selected randomly. For fermentations, randomization was not applicable as no grouping of subjects or samples was required. All strains were processed and analyzed in parallel under identical conditions.
Blinding	No blinding was performed. Blinding was not required as the data were collected using automated and objective measurements (e.g., OD600, metabolite concentrations), and all experimental conditions were processed in parallel.

Reporting for specific materials, systems and methods

We require information from authors about some types of materials, experimental systems and methods used in many studies. Here, indicate whether each material, system or method listed is relevant to your study. If you are not sure if a list item applies to your research, read the appropriate section before selecting a response.

Materials & experimental systems

n/a	Involved in the study
☒	☐ Antibodies
☒	☐ Eukaryotic cell lines
☒	☐ Palaeontology and archaeology
☒	☐ Animals and other organisms
☒	☐ Clinical data
☒	☐ Dual use research of concern
☒	☐ Plants

Methods

n/a	Involved in the study
☒	☐ ChIP-seq
☒	☐ Flow cytometry
☒	☐ MRI-based neuroimaging

Plants

Seed stocks	*Report on the source of all seed stocks or other plant material used. If applicable, state the seed stock centre and catalogue number. If plant specimens were collected from the field, describe the collection location, date and sampling procedures.*
Novel plant genotypes	*Describe the methods by which all novel plant genotypes were produced. This includes those generated by transgenic approaches, gene editing, chemical/radiation-based mutagenesis and hybridization. For transgenic lines, describe the transformation method, the number of independent lines analyzed and the generation upon which experiments were performed. For gene-edited lines, describe the editor used, the endogenous sequence targeted for editing, the targeting guide RNA sequence (if applicable) and how the editor was applied.*
Authentication	*Describe any authentication procedures for each seed stock used or novel genotype generated. Describe any experiments used to assess the effect of a mutation and, where applicable, how potential secondary effects (e.g. second site T-DNA insertions, mosiacism, off-target gene editing) were examined.*
