# Supplementary material for: Anaerobic metabolic evolution for homotypic L-valine fermentation
Source: Nat Commun. 2026 May 29;17:6996. doi: 10.1038/s41467-026-73619-7 (PMC13392391; doi:10.1038/s41467-026-73619-7)
Supplement: Supplementary file 19 — Transparent Peer Review file [file 41467_2026_73619_MOESM19_ESM.pdf]

nature portfolio

Peer Review File

Anaerobic metabolic evolution for homotypic L-valine fermentation

Corresponding Author: Professor Sheng Yang

This file contains all reviewer reports in order by version, followed by all author rebuttals in order by version.

Version 0:

Reviewer comments:

Reviewer #1

(Remarks to the Author)
The authors achieved the coupling of L-valine synthesis with cell growth by knocking out pathways such as formate synthesis and introducing the genes ilvCTM and leuDH. Through serial passaging experiments, they successfully transformed the aerobic synthesis of L-valine into anaerobic production. They also analyzed the high-yield mechanisms in the selected high-producing anaerobic strains and speculated on the pathways for redox balance and ATP generation. The article provides an in-depth exploration of anaerobic strain selection methods, with a systematic overall design that is worth promoting in the construction of anaerobic strains. However, the descriptions of the selection methods and their effects in the text are not clear enough, and related issues need to be further improved.
1. In the section “Anaerobic fermentations in flasks,” is the LB culture grown under aerobic or anaerobic conditions?
2. On the metabolic map, indicate the catalytic positions of the genes adhE, ackA, ldhA, mgsA, frd, and pflB, and draw the metabolic pathway from L-valine to L-alanine.
3. The genes expressed are for L-valine production. Why does the evolved strain accumulate a large amount of L-alanine after 12 passages, with an L-valine titer of only 0.6 g/L while L-alanine accumulates to 1 g/L? Was the alanine synthesis pathway not completely knocked out? Is alanine more easily produced under anaerobic fermentation than valine?
4. Figure 1c and Figure 1d, these two passage result figures are not clear enough. Please draw them more clearly to facilitate the observation of the growth and production changes of the strains during the passage process.
5. Overexpression of ilvCTM and leuDH is intended to switch from using NADPH to using NADH. Since NADH is a precursor for L-valine production, why is there overexpression of pntAB and nadK to convert NADH to NADPH? Additionally, after knocking out formate metabolic pathways and overexpressing ilvCTM and leuDH, why is L-valine production coupled with cell growth?
6. What is the ATP generation and consumption situation in strain S1.0? Is there any calculation? Is it balanced? What about the ATP generation and consumption in the selected strain E1.0? Similarly, are the generation and consumption of NADH balanced in both strains?
7. “Subsequently, the initial glucose concentration was gradually increased to 80 g/L, and the passage interval was shortened to 24 hours.” In the passage process, how is the duration of each generation determined before transferring the culture, and what is the basis for this determination? What is the basis for changing the initial glucose concentration?
8. In Figure 1e, the L-valine production of the strain shows an increasing trend at 60 hours. Why did the fermentation suddenly stop? If the fermentation time is extended, will the yield increase further?
9. Was ilvCTM and leuDH introduced into E8782? If so, were the native ilvC and ilvE genes knocked out?
10. ilvC and ilvE utilize NADPH, while the introduced ilvCTM and leuDH utilize NADH, with the aim of coupling with growth. However, sequencing revealed an increase in the copy number of the native ilvC and ilvE genes. Does this affect the coupling with growth? Is it contrary to the purpose of the modification?
11. “Supplementary Figure 4”should be Supplementary Figure 3.
12. In the section “Rapid reconstruction of the genotype of hyperproducer cells,” the first experiment seems to have little significance. The focus of this article is on how to change aerobic production strains into anaerobic L-valine production strains, rather than further increasing the yield of D-lactate in a strain that can already produce anaerobically.
13. Why is the passage time shortened when glucose is below 10 g/L? Why is it sometimes increasing glucose concentration and sometimes shortening the culture time? What is the basis for these decisions?
14. How do the yield and productivity compare between S1.0 and E1.0?
15. “The total evolution time of 4192 hours (Supplementary Figure 5a and b) represented a 20% improvement over S1.0, which required 5153 hours.” With only one strain tested, how can it be proven whether this is a coincidence or a necessity?

16. During the passage process, the bacterial culture in the same container is generally mixed. After determining that the mixed culture is high-yielding, how can the highest-yielding single strain be isolated from the selected high-yielding mixed culture?
17. Was E2.5 isolated from a high-yielding mixed culture after passaging from S2.0? Please explain this clearly and supplement the text with the isolation process of E2.5.
18. After sequencing E1.0, the mutations were introduced into S1.0 to construct strain S2.0, and the mutations from E2.5 were introduced into S2.0 to construct strain S2.2. Was this done to construct a strain with a clear genetic background? Why not directly use the high-yielding strains obtained through passaging?
19. “Then we decide to combine the ygaZH (p.W152fs), alaE (p.S142_Q145delinsK), lrp (p.P139L), nagA (p.G141fs), thrA (upstream C-T), thrA (p.S354Y) to verify if we can reconstruct a genetically defined strain with a phenotype like that of E1.0.” Figure 2c shows that the L-valine yield significantly decreased after the thrL mutation was reverted. Why was thrL not introduced here?
20. “This discrepancy may be attributed to an epistatic effect arising from differences in the variant order during evolution versus reconstruction.” Does the order of genetic locus modification affect the final yield of the strain? Is there any evidence for this? Theoretically, modifications made earlier and later should not conflict.
21. The figure legends in the entire article are too simplistic and not well-annotated, which severely affects the understanding of the article. They need to be supplemented and improved. Additionally, the DNA sequences of ygaZ, ygaH, nagA, and alaE should be respectively supplemented in Figure 4b, Figure 4c, and Figure 4e. The figures are not labeled, making them difficult to understand.
22. “We hypothesize that accumulated GlcNAc-6-P is exported as GlcNAc, which is then imported back through the phosphotransferase system.” Can it be proven?
23. Is the excess NADH and NADPH consumed through the threonine synthesis pathway to achieve redox balance?
24. If LeuDHv22I catalyzes the conversion of pyruvate to L-alanine, it would compete with the metabolic flux for L-valine production, thereby reducing the yield of L-valine. How can this situation be avoided through genetic modification?

Reviewer #2

(Remarks to the Author)
I co-reviewed this manuscript with one of the reviewers who provided the listed reports. This is part of the Nature Communications initiative to facilitate training in peer review and to provide appropriate recognition for Early Career Researchers who co-review manuscripts.

Reviewer #3

(Remarks to the Author)
This manuscript describes the development of an E. coli strain for the anaerobic production of L-valine using a combination of rational metabolic engineering and adaptive laboratory evolution (ALE). Sequencing has been used to genetically characterize the strains obtained via ALE.

While the authors have carried out quite a number of elaborate experiments and strain characterizations and succeeded in constructing strains that produce L-valine anaerobically, I see only a relatively small performance improvement compared to previously developed strains. Also, referencing of previous work is partially missing and for some major conclusions drawn by the authors evidence is not sufficient.

Major points:

(1) My major criticism concerns the novelty and significance of the work. First of all, the introduction is far from being complete regarding previous works on engineering E. coli for L-valine production. Just a single paper on an E. coli strain producing L-valine has been cited in the introduction although there are a couple of highly relevant papers on constructing E. coli L-valine producer strains (later in the manuscript, some of them have been cited e.g. [18,23,24], but in other contexts). To let the reader fairly assess the novelty and significance, a much clearer description of relevant previous work should be given (e.g. in a table) listing previously introduced metabolic interventions as well as achieved performance measures (titer, yield, productivity). This is very important, also because many of the genetic interventions introduced by the authors in their initial strain (before introducing further manipulations) have indeed been proposed and originally used in other works (e.g., enzyme variants that use NADH instead of NADPH, enzymes without feedback inhibition, deletion of some fermentative pathways). Second, the best L-valine production strain presented by the authors synthesized, in a large-scale anaerobic fermentation (320 m3 fermenter), L-valine from glucose with a titer of 82.1 g/L, yield of 0.55 g/g glucose (85% of the maximum yield), and with a volumetric productivity of 1.37 g/L/h. These key performance indicators are indeed rather high compared to bioprocesses for other chemicals. However, one published aerobic L-valine producer strain of E. coli (84g/L, 0.41g/g glucose, 2.33 g/L; see cited reference [7]) reaches even a higher titer and productivity, only the yield is slightly lower. Moreover, the initial strain S1.0 (and the evolved strain E1.0) is alanine auxotrophic and supplementation of the medium with this amino acid is thus needed, which is a disadvantage for industrial application. Hence, the best strain developed achieves only partial improvements, if at all, which is not clearly stated (again, having a table with the most relevant previously developed strains and their engineering targets and performances would be very helpful at this point).

(2) I’m missing a clear explanation why the constructed strain, with uses L-valine as the only fermentation product, can grow anaerobically. While the pathway from glucose to valine is indeed redox-balanced, it has previously been shown that biomass synthesis in E. coli leads to a small but significant excess of reduction equivalents (see e.g.

https://doi.org/10.1186/s13068-023-02395-z), which must be balanced by the usual fermentation pathways functioning as redox sinks (e.g. with ethanol or succinate as products). However, the L-valine pathway cannot be used for this purpose because it is redox-neutral with glucose as substrate. In fact, this might be the reason why some of the evolved strains seem to produce threonine which could indeed function as redox sink. Also, if the cells take up alanine, this could reduce the formation of NADH, since 2 molecules of NADH are produced in the net stoichiometry if 2 molecules of alanine are synthesized from one molecule of glucose. The whole discussion on redox balancing in the used strains remains thus suspicious.

(3) Another conclusion drawn by the authors, where evidence is not sufficient, is the suggested futile cycle between GlcNac and GlcNac-6-P induced by the mutation in nagA during ALE (also shown in Figure 5). First of all, it remains unclear in Figure 5 how Glc-Nac-6-P gets dephosphorylated at all – is there an enzyme with this functionality? Likewise, how is the GlcNac (-6-P) exported? Also, if the hypothesis would be true, some GlcNac should be in the medium. Has this been measured? Finally, this futile cycle might increase glucose uptake but it will reduce growth yield (and rate). Why should ALE lead to such a phenotype? I find this entire explanation around the GlcNac futile cycle not convincing.

(4) A rather large part of the manuscript deals with the strains S2.0 and E2.5, especially with the genomic analysis of the evolved strain E2.5 and individual gene reversions in the wild-type strain. However, as far as I can see, none of these investigations led to a new superior production strain (compared to E1.0) and they are therefore only of limited interest.

Minor points:

(5) First paragraph of Results section: there is a strange text body: “(Fed-Batch Culture of Escherichia coli for L-Valine Production Based on In Silico Flux Response Analysis)”. Is this a reference?

(6) There are quite a number of typos (e.g., crutial, benefitial) and grammar errors. The manuscript therefore needs some language editing.

(7) The manuscript has neither page nor line numbers, which makes referencing sentences and text difficult for the reviewer. Also, the manuscript would be much easier to read if the figures would be embedded in the main text which is allowed for the first submission.

Reviewer #4

(Remarks to the Author)
The manuscript describes the evolution of an L-valine high-producing Escherichia coli strain through adaptive laboratory evolution (ALE). While the authors report achieving high titers and yields in a 320 m^3 fermenter, significant concerns exist regarding the experimental design, data interpretation, and scientific rigor. The major limitations in methodology and study design fundamentally undermine the novelty and impact of the study, making it unsuitable for publication in Nature Communications in its current form.

Major Concerns

Lack of Quantitative Justification for Anaerobic Fermentation ClaimsThe authors claim anaerobic fermentation reduces sugar consumption and simplifies the process, but they fail to provide quantitative evidence. A comparison with existing studies on L-valine production under aerobic, semi-anaerobic, and anaerobic conditions—including yield (g/g glucose), productivity (g/L/h), and titer (g/L)—is necessary to substantiate these claims. Without such a comparative analysis, the purported benefits of anaerobic fermentation remain speculative.

Flawed Experimental Design in Adaptive Laboratory Evolution (ALE)The study aims to develop an anaerobic L-valine high-producer, but the ALE design does not ensure that L-valine production is the primary driver of fitness improvements. Ideally, ALE should be structured so that L-valine synthesis is the only redox-balancing mechanism, ensuring that evolved strains exhibit increased L-valine production as a direct consequence of selection pressure. However, in the present study, enzymes such as DapB (which can use both NADH and NADPH) and other amino acid synthesis pathways may have contributed to redox balancing, confounding the evolutionary trajectory. The absence of amino acid supplementation to suppress alternative metabolic routes further weakens the experimental design, allowing unintended evolutionary outcomes.

Limited Applicability of the Evolutionary StrategyThe issues in ALE design extend to previous alanine evolution experiments, raising questions about why only the alanine pathway was selectively deleted. If alternative amino acid pathways remained intact, they could have contributed to redox balancing and altered metabolic flux, affecting evolutionary outcomes. Additionally, the rationale for using alanine ALE to validate anaerobic adaptive evolution remains unclear. A clear definition and justification for anaerobic adaptive evolution are missing from the manuscript, making it difficult to evaluate the broader applicability of this approach.

Suboptimal Gene Expression StrategiesGiven the goal of maximizing L-valine production, the reliance on genomic integration rather than high-copy-number plasmids is questionable. High-copy plasmids could have been used to enhance the expression of key pathway genes. The authors should have conducted experiments comparing the final evolved strain with strains carrying high-copy-number plasmids to determine the most effective gene expression strategy.

Figure Annotations and Data Presentation Issues

Figure 1A lacks essential information on gene deletions, making it difficult to follow the strain design strategy.

In Figure 2D, missing titer values for S2 and S2.2 reduce the clarity of comparisons.

Mutation profiles should be clearly labeled beneath the x-axis in Figure 2D.

Inappropriate or Missing ReferencesThe claim that lrp upregulates ilvBN and ygaZH (Page 7) lacks appropriate citation. Reference 19 does not support this assertion. A more suitable reference should be provided.

Unjustified Deletion of ilvBNmutThe rationale for deleting ilvBNmut, a key step in the L-valine pathway, is not explained. Given its central role in anaerobic L-valine production, its deletion requires justification. Additionally, the selection of the ldhA promoter for alaD expression is not rationalized. Given the availability of synthetic promoters for fine-tuned expression, the choice of ldhA should be explicitly justified.

Contradictions Regarding Alanine SupplementationDespite deleting alaA and alaC, the strain still accumulated alanine. If alanine accumulated, why was supplementation still required? This contradiction raises concerns about the accuracy of the metabolic engineering approach. Additionally, the claim that C-terminal mutations in AlaE enhance alanine efflux lacks supporting experimental evidence. A transporter assay quantifying alanine export is needed to validate this claim.

Potential Membrane Stress Due to pntAB OverexpressionOverexpression of pntAB, a membrane protein, could induce membrane stress. Was experimental validation conducted to assess this? A membrane stress assay or fluorescence-based integrity staining would be necessary to address this concern.

Incomplete Valine Production Data

Figure 1E lacks valine concentration data before 12 hours, which is crucial for demonstrating whether valine production is growth-coupled.

The text refers to a 50% increase in biomass and 20% increase in valine production in Figure 1B, but the figure annotation is unclear. Proper verification and correction are needed.

Statistical Validation Deficiency

Statistical significance should be reported for all figures. Confidence intervals, standard deviations, or p-values are missing, reducing the credibility of the data.

Misleading Terminology

The term "homotypic valine fermentation" is not well-defined in metabolic engineering. The claim that valine is the "sole product" needs experimental verification demonstrating the absence of significant byproducts.

Poorly Structured Introduction

The Introduction lacks clarity and depth, failing to effectively frame the study’s objectives and significance.

Severe Manuscript Formatting and Language Issues

The manuscript contains numerous grammatical errors, inconsistent formatting, missing line numbers, and overall poor organization. Such deficiencies do not meet the basic standards of a peer-reviewed submission.

Conclusion

This manuscript presents a conceptually interesting approach but suffers from fundamental flaws in experimental design, data interpretation, and manuscript preparation. The limited applicability of the ALE strategy, poorly controlled evolutionary trajectories, lack of statistical validation, and failure to compare results with existing studies undermine the novelty and significance of the work. Furthermore, the manuscript does not meet the editorial and linguistic standards required for publication in Nature Communications. Given these critical shortcomings, I strongly recommend rejecting this manuscript in its current form.

Version 1:

Reviewer comments:

Reviewer #1

(Remarks to the Author)
1. “Since the endogenous IlvC (for α-acetolactate reduction) and IlvE (for α-ketoisovalerate transamination) are NADPH-dependent, whereas IlvCTM and LeuDH are NADH-dependent, we introduced two cofactor-balancing enzymes encoded by pntAB and nadK. pntAB encodes pyridine nucleotide transhydrogenase, which can catalyze the reversible transfer between NADH and NADP+ (Figure 1a and Figure 5). nadK encodes NAD+ kinase, which catalyzes the phosphorylation of NAD+ to NADP+ using ATP (Figure 1a and Figure 5). This design lets the strain choose the optimal ratio between NAD(H) and NADP(H)." and “To explain how L-valine production was coupled with cell growth: we deleted all native NADH consuming pathways, so in the absence of an NAD^+ regeneration route, cell growth would be severely impaired or even halted. By overexpressing ilvCTM and leuDH, we enabled the production of L-valine, which provided an NADH sink to relieve the intracellular redox imbalance, thereby linking L-valine biosynthesis to cell growth.” There seems to be a contradiction between these two paragraphs. If introducing ilvCTM and leuDH to consume NADH is key for coupling L - valine production with growth under anaerobic conditions, then introducing pntB would also consume NADH. In that case, would introducing pntB to consume NADH affect the coupling of strain growth and production ? Additionally, is NADPH the same as NADH, and would it affect the strain's growth?
2. Please list the total amounts of ATP and NADH generated and consumed under anaerobic conditions.

Reviewer #2

(Remarks to the Author)
I co-reviewed this manuscript with one of the reviewers who provided the listed reports. This is part of the Nature Communications initiative to facilitate training in peer review and to provide appropriate recognition for Early Career Researchers who co-review manuscripts.

Reviewer #3

(Remarks to the Author)
In the revised version of their manuscript the authors addressed several issues raised in my previous report. However, there are still several points that have not yet been solved in a satisfactory manner.

1) I still find the subsections that go beyond strain E1.0 (text between line 435- 664, which is >50% of the Results section) rather verbose and that they contain no or only little advancements. Indeed, the best strain of this manuscript shown in Table 1 refers to strain E1.0. I would either completely remove this part or heavily condense it (e.g. to one page) to convey only the most relevant and evident findings. For example, the explanation regarding a potential futile cycle between GlcNAc-6-P and GlcNAc remains very vague with only very little evidence supporting this hypothesis. This part should be removed.

2) Please add information on the used media (minimal media with carbon source + (carbon) additives such as alanine or yeast extract) in Table 1.
Also regarding Table 1, the authors wrote “We report this highest yield reported to date (Table 1), which has reduced the production cost of L-valine at Meihua Group significantly, as glucose constitutes the largest portion of the production cost components of L-valine.” Does this mean that the process/strain developed by the authors is now used by the Meihua group (“has reduced”!)? Otherwise please rephrase!

3) The authors responded satisfactorily to my question why threonine must be produced by the cells (redox balancing due to excess of reduction equivalents synthesized during growth) – this explanation (maybe in a slightly condensed version) should also be added to the manuscript.

4) One thing that I did not mention in my first review: I think it is mandatory and standard to provide (for the best strain E1.0) the specific growth rate, specific valine production rate and specific glucose uptake rate (at least for the exponential growth phase) to be able to assess the specific productivity of the evolved strain. I also agree with reviewer 4 that valine production should be shown for the entire time period (not only for >12h) in Fig. 1e.

6) Further language improvements are required.

Version 2:

Reviewer comments:

Reviewer #1

(Remarks to the Author)
All my doubts have been resolved.

Reviewer #2

(Remarks to the Author)
I co-reviewed this manuscript with one of the reviewers who provided the listed reports. This is part of the Nature Communications initiative to facilitate training in peer review and to provide appropriate recognition for Early Career

Researchers who co-review manuscripts.

Reviewer #3

(Remarks to the Author)
The authors addressed further points raised in my review of the second round, though not all of them . In particular, the discussion on the very hypothetical GlcNAc / GlcNAc-6P cycle is still part of the manuscript (now moved to Discussion section).

A new major issue emerged in connection with the changes introduced by the authors: As requested, the authors added the full time course of the valine concentration in Figure 1e. With that, I discovered a serious inconsistency: after about 10 hours, the glucose concentration is approximately 18 g/L and the valine concentration ca. 28 g/L. For the following 35 h (until 45 h) almost no glucose is consumed while the valine concentration increases steadily with high rates up to about 65 g/L. How can this be, what is the carbon source for valine production in that period? Also, when dividing the final valine titer (83.g/L) by the initial glucose concentration (ca. 38 g/L) the valine yield is above 2 g/g and thus far above the maximum yield of 0.65 g/g. Again, how can this happen? Similar inconsistencies can be seen in the time course in Supplementary Figure 4d.

Reviewer #4

(Remarks to the Author)
The revised manuscript claims to demonstrate anaerobic “homotypic L-valine fermentation” in E. coli, achieving titers above 80 g/L with near-theoretical yields. While this would represent a significant advance, the data presented remain internally inconsistent and scientifically implausible.
In particular, Figure 1e shows ~80 g/L valine accumulation while only ~30 g/L glucose is consumed. Moreover, between 10–50 h, ~50 g/L valine is reported to be produced despite negligible glucose consumption. These results are incompatible with basic stoichiometric and redox balances and raise serious concerns about data reliability. The authors’ rebuttal did not provide satisfactory clarification or corrected datasets.
Additional contradictions regarding redox balancing (IlvCTM/LeuDH vs. pntAB/nadK roles) and the overextended discussion of strains beyond E1.0 further weaken the manuscript. Given these unresolved issues and the lack of verifiable evidence supporting such extraordinary fermentation performance, I cannot recommend this work for publication in Nature Communications.

Version 3:

Reviewer comments:

Reviewer #3

(Remarks to the Author)
With the extended explanations in the text and legend of Figure 1 (together with the numbers of the fed-bacth process provided in the Supplementary Table 12) I can now confirm consistency of the data and discussed results of the large-scale fermentation.

I do not have any further major point.

Reviewer #4

(Remarks to the Author)
Overall Assessment
The revised manuscript entitled “Anaerobic metabolic evolution for homotypic L-valine fermentation” describes metabolic engineering and laboratory evolution strategies in E. coli for achieving high-yield anaerobic L-valine production. The study addresses an important challenge in amino acid biotechnology, and if valid, the reported yields (>80 g/L, ~85% of theoretical maximum) would represent a major breakthrough.
However, despite extensive revisions and a detailed rebuttal, the manuscript continues to present data inconsistencies, stoichiometric implausibilities, and insufficient methodological transparency, which undermine the reliability of the reported claims.

Major Concerns
1. Stoichiometric Inconsistencies in Fed-Batch Data
• Although the rebuttal explains that this is due to fed-batch glucose feeding, the manuscript presentation (residual glucose only) remains misleading. Yield calculations must be based on total glucose consumed, not initial charge or residual concentrations.
• The authors now provide supplementary calculations showing 20.98 t valine from 38.17 t glucose (yield ~0.55 g/g). However, these data were not transparently integrated into the main figures, and earlier inconsistencies (e.g., Supplementary Figure 4d) were simply removed rather than corrected.
Recommendation: The manuscript must include (i) complete time-course profiles of glucose consumed (not just residual), (ii) corresponding feeding rates, and (iii) reconciled carbon and redox balances. Without this, the data remain unreliable.

2. Data Credibility and Removal of Figures
• The removal of Supplementary Figure 4d (where inconsistencies were most evident) raises concern that the authors are omitting problematic datasets rather than correcting them.
• Extraordinary claims (83.6 g/L valine at ~85% theoretical yield in a 320 m^3 reactor) require extraordinary evidence. At present, the documentation is insufficient.

3. Redox Balancing Contradictions
• The role of IlvCTM/LeuDH (NADH-dependent) vs. pntAB/nadK (NADPH balancing) remains contradictory. The rebuttal argues that evolution selected the optimal redox pathway, but this explanation is speculative without experimental validation.
• Transcriptomic or flux data are needed to support claims that NADH oxidation was effectively coupled to valine synthesis.

4. Overextension Beyond E1.0
• The manuscript devotes significant space to later strains (E2.5, S2.0, etc.), which distracts from the central claim of demonstrating homotypic valine fermentation.
• Given the unresolved issues in E1.0 datasets, extensive discussion of derivative strains weakens the narrative rather than strengthening it.

Minor Points
1. Clarity of Methods – The fed-batch operation must be fully described in the Methods with feeding volumes, timings, and glucose concentrations.
2. Terminology – The term “homotypic fermentation” is not standard and should be more rigorously defined.
3. Discussion – The speculative mechanistic explanations (e.g., gain-of-function mutations in AlaE) are interesting but would benefit from stronger experimental validation.

Recommendation
Despite substantial revisions, the manuscript still suffers from fundamental inconsistencies in mass balance and data presentation. Until complete feeding/consumption profiles and reconciled stoichiometric analyses are transparently reported, the extraordinary fermentation performance claims cannot be substantiated.

Version 4:

Reviewer comments:

Reviewer #4

(Remarks to the Author)
The revised manuscript makes significant claims of >80 g/L L-valine production at ~85% theoretical yield, but key concerns remain. In particular, OD_{600} values continue rising between 9–50 h despite large-volume glucose feeding (~50 m^3), where a dilution effect would be expected; the authors must explain how such biomass growth is possible. Yield and carbon balance reconciliation is incomplete, with insufficient integration of cumulative glucose consumption, redox balance, and biomass measurements. The redox coupling rationale (NADH vs. NADPH) remains speculative without flux or transcriptomic validation. Given the extraordinary nature of the claims, extraordinary evidence is required, and at present the manuscript does not provide sufficient transparency or experimental validation to ensure reproducibility.

Version 6:

Reviewer comments:

Reviewer #1

(Remarks to the Author)
Through comprehensive carbon recovery analysis and stoichiometric redox balancing, this study demonstrates the feasibility of the anaerobic fermentation system with respect to mass and energy conservation. Addressing the dynamics of cellular biomass, this study implemented a two-stage strategy comprising initial aerobic fermentation followed by anaerobic maintenance, which elevated the cellular biomass density while conforming to both physiological principles and process engineering fundamentals. Collectively, the viability of anaerobic L-valine production rests upon stoichiometric constraints (carbon balance and redox homeostasis).

The authors have undergone multiple rounds of revision, supplementing the experiment with additional technical experiments and corresponding mechanistic analyses. As a result, the content and data are now more comprehensive than before.
Therefore, I recommend acceptance of this manuscript.

Open Access This Peer Review File is licensed under a Creative Commons Attribution 4.0 International License, which permits use, sharing, adaptation, distribution and reproduction in any medium or format, as long as you give appropriate credit to the original author(s) and the source, provide a link to the Creative Commons license, and indicate if changes were made.
In cases where reviewers are anonymous, credit should be given to 'Anonymous Referee' and the source.
The images or other third party material in this Peer Review File are included in the article’s Creative Commons license, unless indicated otherwise in a credit line to the material. If material is not included in the article’s Creative Commons license and your intended use is not permitted by statutory regulation or exceeds the permitted use, you will need to obtain permission directly from the copyright holder.
To view a copy of this license, visit https://creativecommons.org/licenses/by/4.0/

Dear Reviewers,

We sincerely appreciate the constructive feedback provided by the reviewers. Your comments and the resulting revisions have substantially improved the quality and clarity of our manuscript.

Point-by-point responses to all reviewer comments (highlighted in blue) are provided, with corresponding revisions clearly marked in red in the manuscript. In response to multiple suggestions, a TRY (Titer, Rate, Yield) benchmarking table (Table 1) has been added, the mechanistic rationale behind the anaerobic strain design has been clarified, and further explanation regarding the cost and purpose of L-alanine supplementation is now included in the response. The manuscript has been thoroughly revised, with additional experiments incorporated where appropriate.

Sincerely,

Sheng Yang

Reviewers' comments:

Reviewer #1 (Remarks to the Author):

The authors achieved the coupling of L-valine synthesis with cell growth by knocking out pathways such as formate synthesis and introducing the genes ilvCTM and leuDH. Through serial passaging experiments, they successfully transformed the aerobic synthesis of L-valine into anaerobic production. They also analyzed the high-yield mechanisms in the selected high-producing anaerobic strains and speculated on the pathways for redox balance and ATP generation. The article provides an in-depth exploration of anaerobic strain selection methods, with a systematic overall design that is worth promoting in the construction of anaerobic strains. However, the descriptions of the selection methods and their effects in the text are not clear enough, and related issues need to be further improved.

1. In the section “Anaerobic fermentations in flasks,” is the LB culture grown under aerobic or anaerobic conditions?

We acknowledge the reviewer's point. Accordingly, we have clarified the method in Line 142-143 as follows: "For L-valine, isolated single colonies of the evolved strain were streaked on plates and then inoculated into 4 mL of LB liquid medium, cultured at 37°C and 240 rpm aerobically for 24 hours."

2. On the metabolic map, indicate the catalytic positions of the genes adhE, ackA, ldhA, mgsA, frd, and pflB, and draw the metabolic pathway from L-valine to L-alanine.

We agree and have added a detailed map in the supplementary Figure 1b and cited in Figure 1a.

3. The genes expressed are for L-valine production. Why does the evolved strain accumulate a large amount of L-alanine after 12 passages, with an L-valine titer of only 0.6 g/L while L-alanine accumulates to 1 g/L? Was the alanine synthesis pathway not completely knocked out? Is alanine more easily produced under anaerobic fermentation than valine?

Thanks for the insightful comment. The accumulation of L-alanine was because its biosynthetic pathway was not completely disrupted at the initial stage (Strain S8975). We have addressed this in the revised manuscript (Line 291-294): "Two possible factors may explain the weak coupling between cell growth and L-valine fermentation. One is that our anaerobic adaptive evolution approach may be suboptimal. The other is the remaining L-alanine synthesis pathway via *alaA* and *alaC*, which may have diverted pyruvate (Supplementary Figure 1b) and make L-alanine the major product."

To eliminate L-alanine by-production and ensure L-valine is the sole product, we then deleted the two L-alanine aminotransferase genes, *alaA* and *alaC*, in the evolved strain S8975. As discussed in Line 310-314: "To make L-valine the sole product and eliminate the by-production of L-alanine in the evolved S8975, we further deleted two L-alanine aminotransferases, *alaA* and *alaC*[29], which are involved in L-alanine synthesis (Supplementary Figure 1c). As expected, the resulting strain S8975 (△*alaA*, △*alaC*) (Supplementary Table 2) did not produce L-alanine, indicating the accumulation of L-alanine was eliminated.".

Although indirectly, *alaA* and *alaC* also consume NADH. Therefore, when other NADH-consuming pathways are blocked, the production of L-alanine under anaerobic conditions becomes favorable.

4. Figure 1c and Figure 1d, these two passage result figures are not clear enough. Please draw them more clearly to facilitate the observation of the growth and production changes of the strains during the passage process.

Thanks for the suggestion! For the typographical reason, we haven't found a method to make them bigger in Figure 1. We tried our best to make them bigger as an alternative in Supplementary Figure

4e and f. At the same time, we added the detailed data in Supplementary Table 5.

5. Overexpression of ilvCTM and leuDH is intended to switch from using NADPH to using NADH. Since NADH is a precursor for L-valine production, why is there overexpression of pntAB and nadK to convert NADH to NADPH? Additionally, after knocking out formate metabolic pathways and overexpressing ilvCTM and leuDH, why is L-valine production coupled with cell growth?

Thanks for the constructive comment. The reason we did not predetermine a specific redox recycling pathway is that it was unclear which route the strain would preferentially utilize under anaerobic conditions. Instead, we allowed the evolutionary process to select the optimal pathway. We explained this in the revised manuscript in Line 319-325: "Since the endogenous IlvC (for α-acetolactate reduction) and IlvE (for α-ketoisovalerate transamination) are NADPH-dependent, whereas IlvCTM and LeuDH are NADH-dependent, we introduced two cofactor-balancing enzymes encoded by *pntAB*[9,30] and *nadK*[31,32]. *pntAB* encodes pyridine nucleotide transhydrogenase, which can catalyze the reversible transfer between NADH and $NADP^+$ (Figure 1a and Figure 5). *nadK* encodes NAD^+ kinase, which catalyzes the phosphorylation of NAD^+ to $NADP^+$ using ATP (Figure 1a and Figure 5). This design lets the strain choose the optimal ratio between NAD(H) and NADP(H). "

To explain how L-valine production was coupled with cell growth: we deleted all native NADH-consuming pathways, so in the absence of an NAD^+ regeneration route, cell growth would be severely impaired or even halted. By overexpressing *ilvC*^{TM} and *leuDH*, we enabled the production of L-valine, which provided an NADH sink to relieve the intracellular redox imbalance, thereby linking L-valine biosynthesis to cell growth. Regarding the strain design principle, we have added relevant content to the Introduction (Line 55-65) to clarify our approach: " Anaerobic growth rescue is a strategy that leverages microorganisms to restore or enhance growth under anaerobic conditions by maintaining intracellular redox balance[13]. This is generally achieved by coupling the desired reaction(s) to NAD^+ regeneration, thereby alleviating the redox imbalance caused by the inactivation of NADH-consuming pathways[13]. This strategy has enabled the transformation of *E. coli* from mixed-acid fermentation[14] to the production of n-butanol[13], D-lactate[15], succinate[16], ethanol[17,18], and L-alanine[19]…… "

And also in results, Line 269-282：

"To leverage anaerobic growth rescue for homotypic L-valine fermentation, the reduction of α-acetolactate and reductive amination/transamination of α-ketoisovalerate represent the two key steps that can consume the NADH generated from glycolysis. To couple L-valine production with NADH oxidation, we……"

Hope these can address the reviewer's concerns.

6. What is the ATP generation and consumption situation in strain S1.0? Is there any calculation? Is it

balanced? What about the ATP generation and consumption in the selected strain E1.0? Similarly, are the generation and consumption of NADH balanced in both strains?

Thanks for your comment. The cofactor generation and consumption must have been balanced. Otherwise, we would not have observed the growth in either strain. If your question pertains to the rate of cofactor recycling, we can say in confidence that the rate of E1.0 is much higher than that of S1.0 as evidence of faster accumulation of L-valine.

7. "Subsequently, the initial glucose concentration was gradually increased to 80 g/L, and the passage interval was shortened to 24 hours." In the passage process, how is the duration of each generation determined before transferring the culture, and what is the basis for this determination? What is the basis for changing the initial glucose concentration?

Thank you for pointing out that this was unclear. The initial glucose concentration is 20 g/L and the duration is 48 hours. When more than half of the glucose is consumed within the duration, we choose to increase the glucose concentration. When the glucose concentration increased to 80 g/L and more than half of the glucose was consumed within the duration at the same time, we chose to shorten the duration time. The detailed data of anaerobic metabolic evolution is shown in Supplementary Table 5 now.

We have added more detailed explanations in Line 353-357 "Regarding the anaerobic metabolic evolution strategy, we gradually increased glucose concentration to simulate industrial conditions and shortened subculture intervals to promote mutation by maintaining high cell division activity. In general, when over half of the glucose was consumed, we raised the initial concentration. Once it reached 80 g/L, we further reduced the passage duration."

8. In Figure 1e, the L-valine production of the strain shows an increasing trend at 60 hours. Why did the fermentation suddenly stop? If the fermentation time is extended, will the yield increase further?

We sincerely appreciate the reviewer's insightful comment. Fermentation was stopped at this point because prolonged fermentation would cause L-valine to exceed its solubility (88.5 g/L in water at 25°C) and crystallize, leading to inconvenience in subsequent processing. Additionally, further extending the fermentation time is unlikely to significantly improve the yield.

9. Was ilvCTM and leuDH introduced into E8782? If so, were the native ilvC and ilvE genes knocked out?

Thanks for the comment. Yes, we introduced *ilvC*^{TM} and *leuDH* into E8782 and didn't knock out the native *ilvC* and *ilvE*.

10. ilvC and ilvE utilize NADPH, while the introduced ilvCTM and leuDH utilize NADH, with the aim of coupling with growth. However, sequencing revealed an increase in the copy number of the native ilvC and ilvE genes. Does this affect the coupling with growth? Is it contrary to the purpose of the modification?

We appreciate the reviewer's comment. The sequencing revealed an increased copy number of the native *ilvC, ilvD, ilvE*, and the exogenous *leuDH*. The increased copy number of native *ilvC* and *ilvE* does not affect the coupling with growth, nor does it contradict the intended modification. This is because the system is capable of regenerating both NAD^+ and $NADP^+$ by enhancing transhydrogenase encoded by *pntAB* and also the NAD kinase, ensuring cofactor availability for both pathways (also see response 5).

11. "Supplementary Figure 4"should be Supplementary Figure 3.

Thanks for the careful review. We have corrected this mistake.

12. In the section "Rapid reconstruction of the genotype of hyperproducer cells," the first experiment seems to have little significance. The focus of this article is on how to change aerobic production strains into anaerobic L-valine production strains, rather than further increasing the yield of D-lactate in a strain that can already produce anaerobically.

We agree and have deleted this misleading part.

13. Why is the passage time shortened when glucose is below 10 g/L? Why is it sometimes increasing glucose concentration and sometimes shortening the culture time? What is the basis for these decisions?

Thank you for pointing out that this was unclear. We have addressed this problem in comment 7.

14. How do the yield and productivity compare between S1.0 and E1.0?

Thanks for the suggestion. We included the productivities of S1.0 (Line 328) and E1.0 (Line 371) in the manuscript, which are 0.16 and 1.38/1.84 respectively. For the yields, E1.0 is 0.55/0.56 in 320 m^3 bioreactor and S1.0 is 0.55.

15. "The total evolution time of 4192 hours (Supplementary Figure 5a and b) represented a 20% improvement over S1.0, which required 5153 hours." With only one strain tested, how can it be proven whether this is a coincidence or a necessity?

We completely understand the reviewer’s concern. To address this, we conducted parallel evolution experiments with five S2.0 strains, and the selected strain exhibited the best phenotype. Other evolved strains also demonstrated varying degrees of L-valine production improvement, with evolution speeds either exceeding or being comparable to S1.0. This suggests that the observed improvements are unlikely to be purely coincidental. We have added the following clarification in the revised manuscript in Line 457-461: “The total evolution time of 4192 hours (Supplementary Figure 6a and b) represented a 20% improvement over S1.0, which required 5153 hours. The other four independent and parallel evolved strains showed varying degrees of improvement in L-valine production, and their evolution speeds either exceeded or were comparable to that of S1.0. This suggests that the newly added targets in S2.0 compared to S1.0 accelerated the evolution.”

16. During the passage process, the bacterial culture in the same container is generally mixed. After determining that the mixed culture is high-yielding, how can the highest-yielding single strain be isolated from the selected high-yielding mixed culture?

We understand the reviewer’s concern. To isolate the highest-yielding single strain from the selected high-yielding mixed culture, we picked and compared three individual clones from streaked plates. We have added the following clarification in the revised manuscript (Line 363-364): “with a glucose consumption rate of approximately 2 g/L/h and a yield around 0.50 g/g, resulting in the isolation of the single-colony strain E8782 (E refers to "evolved") by streaking on the plate (Supplementary Table 2 and 5).”

17. Was E2.5 isolated from a high-yielding mixed culture after passaging from S2.0? Please explain this clearly and supplement the text with the isolation process of E2.5.

We acknowledge the reviewer’s point. As described in Line 141-148, 469 and response to comment 16, we did single-colony isolation to isolate E2.5 from the mixture.

18. After sequencing E1.0, the mutations were introduced into S1.0 to construct strain S2.0, and the mutations from E2.5 were introduced into S2.0 to construct strain S2.2. Was this done to construct a strain with a clear genetic background? Why not directly use the high-yielding strains obtained through passaging?

We understand the reviewer’s concern. Our goal is to construct a strain that has a clear genetic background. In this way, we can have the opportunity to know the mechanisms of hyperproduction. With this knowledge, we can iteratively optimize the strain for better performance.

19. “Then we decide to combine the ygaZH (p.W152fs), alaE (p.S142_Q145delinsK), lrp (p.P139L), nagA (p.G141fs), thrA (upstream C-T), thrA (p.S354Y) to verify if we can reconstruct a genetically defined strain with a phenotype like that of E1.0.” Figure 2c shows that the L-valine yield significantly decreased after the thrL mutation was reverted. Why was thrL not introduced here?

Thanks for the careful review. *thrA* (upstream C-T) is close to *thrL* but not in the *thrL*. We should say *thrA* (upstream C-T) instead of *thrL*. We have corrected this mistake in Figure 2.

20. “This discrepancy may be attributed to an epistatic effect arising from differences in the variant order during evolution versus reconstruction.” Does the order of genetic locus modification affect the final yield of the strain? Is there any evidence for this? Theoretically, modifications made earlier and later should not conflict.

We understand the reviewer’s concern. Our hypothesis is the order of modifications could potentially lead to unknown mutations that influence strain performance.

21. The figure legends in the entire article are too simplistic and not well-annotated, which severely affects the understanding of the article. They need to be supplemented and improved. Additionally, the DNA sequences of ygaZ, ygaH, nagA, and alaE should be respectively supplemented in Figure 4b, Figure 4c, and Figure 4e. The figures are not labeled, making them difficult to understand.

We sincerely appreciate the reviewer’s insightful comment. We have added the length of ORF and the site of mutation in Figure 4b, c, and e. We also included the sequences before and after mutation in Supplementary Table 8.

22. “We hypothesize that accumulated GlcNAc-6-P is exported as GlcNAc, which is then imported back through the phosphotransferase system.” Can it be proven?

Thanks for the insightful comment. We added a discussion in line 572-574 “However, the enzymes responsible for GlcNAc-6-P dephosphorylation and exportation remain unclear. While alternative explanations may exist, we are unable to identify a more plausible mechanism at this stage.”.

23. Is the excess NADH and NADPH consumed through the threonine synthesis pathway to achieve redox balance?

Yes, we agree.

24. If LeuDHv22I catalyzes the conversion of pyruvate to L-alanine, it would compete with the

metabolic flux for L-valine production, thereby reducing the yield of L-valine. How can this situation be avoided through genetic modification?

We understand the reviewer's concern. The conversion of pyruvate to L-alanine by LeuDH^{V22I} occurs at a very low level. The evidence is that the strain cannot grow without supplementation of L-alanine. This suggests that the amount of L-alanine produced by itself is at a low level that cannot even support its growth at the early stage. To totally avoid the side activity of LeuDH on pyruvate, we may screen a variety of LeuDH encoding genes from more sources.

Reviewer #2 (Remarks to the Author):

I co-reviewed this manuscript with one of the reviewers who provided the listed reports. This is part of the Nature Communications initiative to facilitate training in peer review and to provide appropriate recognition for Early Career Researchers who co-review manuscripts.

Thanks for your efforts and detailed review.

Reviewer #3 (Remarks to the Author):

This manuscript describes the development of an E. coli strain for the anaerobic production of L-valine using a combination of rational metabolic engineering and adaptive laboratory evolution (ALE). Sequencing has been used to genetically characterize the strains obtained via ALE.

While the authors have carried out quite a number of elaborate experiments and strain characterizations and succeeded in constructing strains that produce L-valine anaerobically, I see only a relatively small performance improvement compared to previously developed strains. Also, referencing of previous work is partially missing and for some major conclusions drawn by the authors evidence is not sufficient.

Major points:

(1) My major criticism concerns the novelty and significance of the work. First of all, the introduction is far from being complete regarding previous works on engineering E. coli for L-valine production. Just a single paper on an E. coli strain producing L-valine has been cited in the introduction although there are a couple of highly relevant papers on constructing E. coli L-valine producer strains (later in the manuscript, some of them have been cited e.g. [18,23,24], but in other contexts).

We sincerely appreciate the reviewer's insightful comment. While our strain construction builds upon known aerobic engineering principles, the successful translation to a strictly anaerobic, growth-coupled, industrial-scale fermentation represents a fundamental conceptual and technical leap. We added the important relevant papers in the introduction in Line 39-40: "L-valine is produced through microbial fermentation from sugars, most commonly using engineered *Corynebacterium glutamicum* or *Escherichia coli*[3-6] under aerobic conditions." and Line 66-68: " We deleted the NADH-consuming reactions such as mixed-acid fermentation reactions in E. coli to impair its anaerobic growth, then enhanced the L-valine biosynthesis pathway[4-6] to create a driving force for reactions that consume NADH."

To let the reader fairly assess the novelty and significance, a much clearer description of relevant previous work should be given (e.g. in a table) listing previously introduced metabolic interventions as well as achieved performance measures (titer, yield, productivity). This is very important, also because

many of the genetic interventions introduced by the authors in their initial strain (before introducing further manipulations) have indeed been proposed and originally used in other works (e.g., enzyme variants that use NADH instead of NADPH, enzymes without feedback inhibition, deletion of some fermentative pathways).

Thanks for the insightful suggestion. We respectfully note that a ~20% cost reduction for a commodity product such as L-valine represents a major industrial impact that far exceeds incremental academic gains in many strain development studies. We have added the comparison to Table 1 and incorporated the corresponding explanation in the discussion (Line 711–722)： "We would like to emphasize the improved yield from 37% (industrial-scale aerobic process at Meihua Holdings Group) to 56% (our anaerobic process at commercial scale). We report this highest yield reported to date (Table 1), which has reduced the production cost of L-valine at Meihua Group significantly, as glucose constitutes the largest portion of the production cost components of L-valine……"

Table 1 Comparison of L-valine-producing strains.

**Host**	**Culture condition in bioreactor***	**Performance** Titer g/L	Yield g/g glucose	Rate g/L/h	**Reference**
***E.coli* W**	Fed-batch; 6.6-L bioreactor	60.7	0.22	2.06	Ref 6
***E.coli* W3110**	Two-stage aerobic-oxygen limiting fed-batch fermentation; 5-L bioreactor	84	0.41	2.33	Ref 11
***E.coli* ATCC 8739**	Anaerobic fermentation; 320-m^3 bioreactor	82.1	0.55	1.37	This study
***E.coli* ATCC 8739**	Anaerobic fed-batch fermentation with limited oxygen bubbling during 0-4 hours; 320-m^3 bioreactor	82.6	0.56	1.84	This study
C. glutamicum	Aerobic fed-batch fermentation; 320-m^3 bioreactor	85	0.37	1.25	A commercial aerobic process (data provided by Meihua Holdings Group)

* We considered only those strains with available data from fermentation experiments in bioreactors.

Second, the best L-valine production strain presented by the authors synthesized, in a large-scale anaerobic fermentation (320 m3 fermenter), L-valine from glucose with a titer of 82.1 g/L, yield of 0.55 g/g glucose (85% of the maximum yield), and with a volumetric productivity of 1.37 g/L/h. These key performance indicators are indeed rather high compared to bioprocesses for other chemicals. However, one published aerobic L-valine producer strain of E. coli (84g/L, 0.41g/g glucose, 2.33 g/L; see cited reference [7]) reaches even higher titer and productivity, only the yield is slightly lower. Moreover, the initial strain S1.0 (and the evolved strain E1.0) is alanine auxotrophic, and supplementation of the medium with this amino acid is thus needed, which is a disadvantage for industrial application. Hence, the best strain developed achieves only partial improvements, if at all, which is not clearly stated (again, having a table with the most relevant previously developed strains and their engineering targets and performances would be very helpful at this point).

This is a great suggestion. As noted in our previous response, we have added the benchmarking Table 1 for your review. We believe that a 20% increase in yield represents a significant impact. While the final titer is constrained by the solubility limit of L-valine in water, the rate can still be increased. For instance, a brief oxygen supply phase at the beginning accelerates cell growth, reducing the total fermentation time from 60 to 45 hours without compromising yield or titer. Although it is theoretically possible to further increase the yield to 2.33 g/L/h by slightly sacrificing the conversion yield, this would not be cost-effective at the industrial scale.

Although L-alanine was supplemented to enhance E1.0 growth kinetics, it's important to note that the strain is not alanine auxotrophic but rather exhibits slower growth in defined medium without this additive. The supplementation levels were maintained at 0.1 g/L in laboratory-scale cultures and 0.3 g/L in industrial production. Notably, the current market price of at 18,000 yuan/ton represents less than 1% of total feedstock costs for L-valine production. This minimal additive expenditure has been fully accounted for in our cost reduction analysis.

To be clearer, we added relative content to the discussion in Line 711-722: "We would like to emphasize the improved yield from 37% (industrial-scale aerobic process at Meihua Holdings Group) to 56% (our anaerobic process at commercial scale). We report this highest yield reported to date (Table 1), which has reduced the production cost of L-valine at Meihua Group significantly, as glucose constitutes the largest portion of the production cost components of L-valine. The titer of L-valine is limited by its solubility in liquid (88.5 g/L in water at 25°C), and both aerobic and anaerobic fermentation processes have reached this ceiling. Productivity, however, can be improved considerably through process modifications. Our anaerobic process can achieve higher productivity

(by introducing oxygen in the early stage to accelerate biomass accumulation), the fermentation cycle can be reduced from 60 hours to 45 hours, while L-valine production and conversion rates remain essentially unchanged, increasing productivity from 1.37 g/L/h to 1.84 g/L/h based on 320 m^3 demonstration scale data). We are exploring further productivity enhancements through cell recycling or continuous fermentation."

(2) I'm missing a clear explanation why the constructed strain, with uses L-valine as the only fermentation product, can grow anaerobically. While the pathway from glucose to valine is indeed redox-balanced, it has previously been shown that biomass synthesis in E. coli leads to a small but significant excess of reduction equivalents (see e.g. https://doi.org/10.1186/s13068-023-02395-z), which must be balanced by the usual fermentation pathways functioning as redox sinks (e.g. with ethanol or succinate as products). However, the L-valine pathway cannot be used for this purpose because it is redox-neutral with glucose as substrate. In fact, this might be the reason why some of the evolved strains seem to produce threonine which could indeed function as redox sink. Also, if the cells take up alanine, this could reduce the formation of NADH, since 2 molecules of NADH are produced in the net stoichiometry if 2 molecules of alanine are synthesized from one molecule of glucose. The whole discussion on redox balancing in the used strains remains thus suspicious.

Thank you for your valuable and detailed comments. We agree that biomass synthesis in *E. coli* typically generates a slight excess of reducing equivalents, even when the main fermentation pathway is redox-balanced. The reference you cited illustrates how excess NADH can be consumed through the reduction of acetate to ethanol as a redox sink.

The pathways from glucose to L-valine, isobutanol (the ref you cited), ethanol (Kim et al., 2007 , Yomano et al., 2008), succinate (Zhang et al., 2009), lactate (Zhou et al., 2006), n-butanol (Shen et al., 2011), and L-alanine (Zhang et al., 2007), are all redox-balanced, they indeed cannot serve as a redox sink. In our L-valine case, there should have been little excess NADH, and the observed mutations in *thrA* (including a promoter-region C→T mutation and a coding-region S354Y substitution), as you pointed out, serve as the evidence. These mutations relieved L-threonine feedback inhibition and led to the production of small amounts of L-threonine to consume excess NADH. Importantly, the glucose-to-threonine biosynthetic pathway is redox-negative (i.e., consumes 1 mol NADH than it generates), and thus can serve as a native redox sink in the absence of canonical fermentation pathways. This represents a unique and elegant evolutionary solution to a redox challenge in a system engineered for homotypic L-valine production.

In our opinion, L-alanine must have been used for cell growth as a building block for protein synthesis, instead of being dissimilated to reduce NAD^+. Furthermore, the L-alanine amount that supplemented is very little (⩽0.3g/L) compared to D-glucose (⩾140 g/L). We also found the extra L-threonine as an extra NADH sink, which is also a small portion of the products.

Therefore, we do not think they would make significant contribution to redox balance. We sincerely hope that these additions adequately address your queries.

(3) Another conclusion drawn by the authors, where evidence is not sufficient, is the suggested futile cycle between GlcNac and GlcNac-6-P induced by the mutation in nagA during ALE (also shown in Figure 5). First of all, it remains unclear in Figure 5 how Glc-Nac-6-P gets dephosphorylated at all – is there an enzyme with this functionality? Likewise, how is the GlcNac (-6-P) exported? Also, if the hypothesis would be true, some GlcNac should be in the medium. Has this been measured? Finally, this futile cycle might increase glucose uptake but it will reduce growth yield (and rate). Why should ALE lead to such a phenotype? I find this entire explanation around the GlcNac futile cycle not convincing.

We acknowledge that further evidence is needed to fully elucidate the mechanisms of the newly identified targets. However, the reviewer's hypothesis that such cycles necessarily reduce growth yield is unsupported by our observed phenotype. We added a discussion in line 572-574 "However, the enzymes responsible for GlcNAc-6-P dephosphorylation and exportation remain unclear. While alternative explanations may exist, we are unable to identify a more plausible mechanism at this stage.".

(4) A rather large part of the manuscript deals with the strains S2.0 and E2.5, especially with the genomic analysis of the evolved strain E2.5 and individual gene reversions in the wild-type strain. However, as far as I can see, none of these investigations led to a new superior production strain (compared to E1.0) and they are therefore only of limited interest.

We fully understand the reviewer's expectations, and we are also eager to design better-performing strains based on the learned mechanisms. We have found that "Additionally, by introducing *alaE* (p. A149D) based into S2.5, we achieved the L-valine production comparable to E2.5 (Figure 4f), which showed a higher yield and cell density than E1.0 (Supplementary Table 11)." shown in Line 648-650. And we will continue to improve phenotype based on the rationally designed strain.

Minor points:

(5) First paragraph of Results section: there is a strange text body: "(Fed-Batch Culture of Escherichia coli for L-Valine Production Based on In Silico Flux Response Analysis)". Is this a reference?

Thanks for the careful review. We have corrected it in Line 260 as reference 4.

(6) There are quite a number of typos (e.g., crutial, benefitial) and grammar errors. The manuscript therefore needs some language editing.

Thanks for the careful review. We have corrected these typos and checked for grammar errors. Hope this time can achieve the standard.

(7) The manuscript has neither page nor line numbers, which makes referencing sentences and text difficult for the reviewer. Also, the manuscript would be much easier to read if the figures would be embedded in the main text which is allowed for the first submission.

Thank you for the helpful suggestions. We have now included both page and line numbers to facilitate review, and have embedded all figures within the main text.

Reviewer #4 (Remarks to the Author):

The manuscript describes the evolution of an L-valine high-producing Escherichia coli strain through adaptive laboratory evolution (ALE). While the authors report achieving high titers and yields in a 320 m^3 fermenter, significant concerns exist regarding the experimental design, data interpretation, and scientific rigor. The major limitations in methodology and study design fundamentally undermine the novelty and impact of the study, making it unsuitable for publication in Nature Communications in its current form.

Major Concerns

Lack of Quantitative Justification for Anaerobic Fermentation Claims

The authors claim anaerobic fermentation reduces sugar consumption and simplifies the process, but they fail to provide quantitative evidence. A comparison with existing studies on L-valine production under aerobic, semi-anaerobic, and anaerobic conditions—including yield (g/g glucose), productivity (g/L/h), and titer (g/L)—is necessary to substantiate these claims. Without such a comparative analysis, the purported benefits of anaerobic fermentation remain speculative.

Thanks for the insightful suggestion. We have added the comparison to Table 1 and incorporated the corresponding explanation in the discussion (Line 711-722): "We would like to emphasize the improved yield from 37% (industrial-scale aerobic process at Meihua Holdings Group) to 56% (our anaerobic process at commercial scale). We report this highest yield reported to date (Table 1), which has reduced the production cost of L-valine at Meihua Group significantly, as glucose constitutes the largest portion of the production cost components of L-valine.......”

Table 1 Comparison of L-valine-producing strains.

Host	Culture condition in bioreactor*	Performance			Reference
		Titer g/L	Yield g/g glucose	Rate g/L/h	
***E.coli* W**	Fed-batch; 6.6-L bioreactor	60.7	0.22	2.06	Ref 6
***E.coli* W3110**	Two-stage aerobic-oxygen limiting fed-batch fermentation; 5-L bioreactor	84	0.41	2.33	Ref 11
***E.coli* ATCC 8739**	Anaerobic fermentation; 320-m^3 bioreactor	82.1	0.55	1.37	This study
***E.coli* ATCC 8739**	Anaerobic fed-batch fermentation with limited oxygen bubbling during 0-4 hours; 320-m^3 bioreactor	82.6	0.56	1.84	This study
C. glutamicum	Aerobic fed-batch fermentation; 320-m^3 bioreactor	85	0.37	1.25	A commercial aerobic process (data provided by Meihua Holdings Group)

* We considered only those strains with available data from fermentation experiments in bioreactors.

Flawed Experimental Design in Adaptive Laboratory Evolution (ALE)

The study aims to develop an anaerobic L-valine high-producer, but the ALE design does not ensure that L-valine production is the primary driver of fitness improvements. Ideally, ALE should be structured so that L-valine synthesis is the only redox-balancing mechanism, ensuring that evolved strains exhibit increased L-valine production as a direct consequence of selection pressure. However, in the present study, enzymes such as DapB (which can use both NADH and NADPH) and other amino acid synthesis pathways may have contributed to redox balancing, confounding the evolutionary trajectory. The absence of amino acid supplementation to suppress alternative metabolic routes further weakens the experimental design, allowing unintended evolutionary outcomes.

We agree with the statement that “Ideally, ALE should be structured so that L-valine synthesis is the only redox-balancing mechanism, ensuring that evolved strains exhibit increased L-valine production as a direct consequence of selection pressure.” A previous study on anaerobic evolution in L-alanine production (doi: 10.1007/s00253-007-1170-y) has not reported such effects. Moreover, most amino

acid biosynthesis pathways—aside from glycine, L-alanine, L-aspartate, and L-glutamate—are strongly feedback-inhibited by their end products (doi: 10.1016/j.celrep.2017.08.066), which generally prevents their accumulation. That is why we blocked L-alanine production and derepressed L-valine biosynthesis before anaerobic metabolic evolution. Consistent with this rationale, we did not detect the accumulation of glycine, L-aspartate, and L-glutamate. While pathways such as L-threonine are partially derepressed, the resulting flux is minimal, contributing less than ~2% of the carbon compared to the main product, which is likely only sufficient to accommodate excess reducing equivalents during growth.

While the reviewer suggests that DapB may contribute to redox balancing during ALE, we note that DapB is an essential gene required for L-lysine biosynthesis. Importantly, L-lysine accumulation inhibits aspartate kinase (LysC).

We believe the extensive deletions we performed, effectively eliminate all canonical redox sink pathways under anaerobic conditions. The observed outcome, growth strictly dependent on L-valine biosynthesis, confirms the validity of our ALE design. The disrupted competing redox-balancing pathways under anaerobic conditions, such as formate, lactate are described in Line 269-275 "To leverage anaerobic growth rescue for homotypic L-valine fermentation, the reduction of α-acetolactate and reductive amination/transamination of α-ketoisovalerate represent the two key steps that can consume the NADH generated from glycolysis. To couple L-valine production with NADH oxidation, we deleted the competitive NADH-consuming pathways involved in mixed-acid fermentation (ethanol, acetate, lactate, succinate, and formate) by knocking out *adhE, ackA, ldhA, mgsA, frd,* and *pflB*[11,19] in *E. coli*, thereby making L-valine biosynthesis the primary route for intracellular redox balancing.".

In conclusion, we only removed the terminal product inhibition of L-valine synthesis. Therefore, the synthesis of other amino acids would be subject to feedback inhibition and is unlikely to accumulate. DapB is an essential gene which is involved in the L-lysine synthesis. We didn't detect L-lysine accumulation indeed.

Limited Applicability of the Evolutionary Strategy

The issues in ALE design extend to previous alanine evolution experiments, raising questions about why only the alanine pathway was selectively deleted. If alternative amino acid pathways remained intact, they could have contributed to redox balancing and altered metabolic flux, affecting

evolutionary outcomes. Additionally, the rationale for using alanine ALE to validate anaerobic adaptive evolution remains unclear. A clear definition and justification for anaerobic adaptive evolution are missing from the manuscript, making it difficult to evaluate the broader applicability of this approach.

Thanks for the comment! We not only disrupted the L-alanine pathway but also the other mixed-acid fermentation pathways as in Line 269-275 in the last response. During the evolution process, we only detected the accumulation of L-alanine, while other amino acids did not accumulate. Most amino acid biosynthesis pathways—aside from glycine, L-alanine, L-aspartate, and L-glutamate—are strongly feedback-inhibited by their end products (doi: 10.1016/j.celrep.2017.08.066), which generally prevents their accumulation. We did not detect the accumulation of glycine, L-aspartate, and L-glutamate. To eliminate L-alanine by-production and ensure L-valine is the sole product, we then deleted the two L-alanine aminotransferase genes, *alaA* and *alaC*, in the evolved strain S8975. As discussed in Line 310-314: "To make L-valine the sole product and eliminate the by-production of L-alanine in the evolved S8975, we further deleted two L-alanine aminotransferases, *alaA* and *alaC*[29], which are involved in L-alanine synthesis (Supplementary Figure 1c). As expected, the resulting strain S8975 (△*alaA*, △*alaC*) (Supplementary Table 2) did not produce L-alanine, indicating the accumulation of L-alanine was eliminated.".

We also have addressed this in the revised manuscript (Line 291-294): "Two possible factors may explain the weak coupling between cell growth and L-valine fermentation. One is that our anaerobic adaptive evolution approach may be suboptimal. The other is the remaining L-alanine synthesis pathway via *alaA* and *alaC*, which may have diverted pyruvate (Supplementary Figure 1b) and make L-alanine the major product."

As for why we chose L-alanine evolution, we want to confirm our anaerobic metabolic evolution setup was optimal. Therefore, we choose the successful case of L-alanine. We added the reasons in Line 295-299 as" Based on the clues provided by L-alanine accumulation, we aim to first validate the effectiveness of the anaerobic adaptive evolution method using the successful case of L-alanine[19], which replaced the native D-lactate dehydrogenase of *E. coli* with the NADH-dependent alanine dehydrogenase *alaD* from *Geobacillus stearothermophilus* to link cell growth with L-alanine production (Supplementary Figure 1c)."

For the definition and justification for anaerobic adaptive evolution, we added in the introduction in Line Line 55-65: "Anaerobic growth rescue is a strategy that leverages microorganisms to restore or enhance growth under anaerobic conditions by maintaining intracellular redox balance[13]. This is generally achieved by coupling the desired reaction(s) to NAD^+ regeneration, thereby alleviating the

redox imbalance caused by the inactivation of NADH-consuming pathways[13]. This strategy has enabled the transformation of *E. coli* from mixed-acid fermentation[14] to the production of n-butanol[13], D-lactate[15], succinate[16], ethanol[17,18], and L-alanine[19]…… ".

Suboptimal Gene Expression Strategies

Given the goal of maximizing L-valine production, the reliance on genomic integration rather than high-copy-number plasmids is questionable. High-copy plasmids could have been used to enhance the expression of key pathway genes. The authors should have conducted experiments comparing the final evolved strain with strains carrying high-copy-number plasmids to determine the most effective gene expression strategy.

We understand the reviewer's concern. Chromosomal integration is a gold-standard strategy in industrial strain development due to its superior genetic stability and regulatory advantages (https://doi.org/10.1038/nbt.1555). Plasmid-based expression is generally avoided in large-scale fermentation.

We tested whether E1.0Δ*leuDH* could be rescued by plasmid-based complementation wit (p. V22I). However, the highest titer of three colonies is 19.74 g/L in 48 hours, which is far away from the titer of E1.0 (more than 28 g/L in 24 hours). Additionally, we attempted to clone the entire set of L-valine pathway genes, along with the identified mutations, into a high-copy-number plasmid. These efforts were unsuccessful, likely due to the significant metabolic burden imposed by overexpression of the pathway.

Figure Annotations and Data Presentation Issues

Figure 1A lacks essential information on gene deletions, making it difficult to follow the strain design strategy.

Thanks for the suggestion! We agree and have included supplementary figure 1b to illustrate Figure 1A.

In Figure 2D, missing titer values for S2 and S2.2 reduce the clarity of comparisons.

Mutation profiles should be clearly labeled beneath the x-axis in Figure 2D.

Thanks for the powerful suggestion. We added the mutation profiles beneath the x-axis and included

S2.0 and S2.2 in Figure 2D.

Inappropriate or Missing References

The claim that lrp upregulates ilvBN and ygaZH (Page 7) lacks appropriate citation. Reference 19 does not support this assertion. A more suitable reference should be provided.

Thanks for the detailed review. We corrected the mis-citing in Line 458.

Unjustified Deletion of ilvBNmut

The rationale for deleting ilvBNmut, a key step in the L-valine pathway, is not explained. Given its central role in anaerobic L-valine production, its deletion requires justification. Additionally, the selection of the ldhA promoter for alaD expression is not rationalized. Given the availability of synthetic promoters for fine-tuned expression, the choice of ldhA should be explicitly justified.

Thanks for your comment and concern. We did not delete *ilvBN*^{mut} in the L-valine strain. If you are referring to our design in an L-alanine strain, where we deleted *ilvBN*^{mut} while integrating *alaD* to modify the L-valine strain into an L-alanine-producing strain, it's because we chose L-alanine as a case to validate the feasibility of our evolutionary method. The choice of the *ldhA* promoter was based on literature related to L-alanine production (doi: 10.1007/s00253-007-1170-y). We added reference 19 in the manuscript as "where *alaD* is regulated by the promoter of *ldhA*[19] to construct S9015" in Line 300.

Contradictions Regarding Alanine Supplementation

Despite deleting alaA and alaC, the strain still accumulated alanine. If alanine accumulated, why was supplementation still required? This contradiction raises concerns about the accuracy of the metabolic engineering approach. Additionally, the claim that C-terminal mutations in AlaE enhance alanine efflux lacks supporting experimental evidence. A transporter assay quantifying alanine export is needed to validate this claim.

We thank the reviewer for the sharp observation, this point may indeed appear confusing at first glance. To clarify, the strain can grow without exogenous L-alanine, although the growth rate is significantly reduced (to approximately one-fourth of the normal rate) (doi: 10.1128/JB.00738-10). To

facilitate cell growth, we added a small amount of L-alanine at the beginning. After the cells have grown, the L-alanine synthesized via LeuDH probably exceeds cellular requirements and needs to be pumped out. Given the low supplementation level, which can potentially be reduced further, this strategy remains cost-effective overall. Regarding the L-alanine transport assay, we would be happy to include it in the next revision if the reviewer deems it necessary.

Potential Membrane Stress Due to pntAB Overexpression

Overexpression of pntAB, a membrane protein, could induce membrane stress. Was experimental validation conducted to assess this? A membrane stress assay or fluorescence-based integrity staining would be necessary to address this concern.

We thank you for the reviewer's suggestion. Overexpression of *pntAB* in *E. coli* is relatively common, but we did not find direct evidence proving its impact on membrane stress. Inversely, two studies have suggested that overexpression of *pntAB* could enhance membrane stability. In one study (doi: 10.1007/s10295-018-2117-2), overexpression of *pntAB* to enhance glycolic acid biosynthesis in *E. coli* improved cell growth and product concentration. This paper speculated that *pntAB* influences membrane stability indirectly by regulating the NADPH/NADH balance. Another study used a SoxR-based NADPH biosensor to show that the deletion of *pntAB* caused fluctuations in NADPH levels, while overexpression of *pntAB* might indirectly affect membrane structure by maintaining NADPH homeostasis (doi: 10.1002/mbo3.785). The second paper suggested a potential link between the metabolic function of *pntAB* and membrane integrity. We used an L-valine strain with overexpressed *pntAB* in a 320 m^3-scale fermentation, which has been running continuously for over a year, and we did not observe any negative impact on strain growth.

Incomplete Valine Production Data

Figure 1E lacks valine concentration data before 12 hours, which is crucial for demonstrating whether valine production is growth-coupled.

We understand the reviewer’s concern. It is standard industrial practice not to measure L-valine accumulation during the first 12 hours of fermentation. In our study, we have demonstrated that L-valine production is growth-coupled at the 1-L scale (Supplementary Figure 4b and c). The corresponding description is shown in Line 370-372: “In a 1 L anaerobic fermenter, E1.0 reached an L-

valine titer of 72 g/L in 60 hours, with a yield of 0.55 g/g (Supplementary Figures 4b and c)."

The text refers to a 50% increase in biomass and 20% increase in valine production in Figure 1B, but the figure annotation is unclear. Proper verification and correction are needed.

Thanks for the suggestion! We tried to make it bigger and gave the detailed data in Supplementary Table 5.

Statistical Validation Deficiency

Statistical significance should be reported for all figures. Confidence intervals, standard deviations, or p-values are missing, reducing the credibility of the data.

We sincerely appreciate the reviewer's feedback on the statistical analysis of our data. We added statistical significance or p-values in Figure 2a, 2b, 2c, 2d, Figure 4a, 4b, 4d, 4e, 4f, and Supplementary Figure 6c. We hope these modifications can address the reviewer's concerns and demonstrate our commitment to data integrity.

Misleading Terminology

The term "homotypic valine fermentation" is not well-defined in metabolic engineering. The claim that valine is the "sole product" needs experimental verification demonstrating the absence of significant byproducts.

Thanks for the suggestion. In homolactic fermentation, lactic acid is the primary product, and the theoretical yield is 1 mol/mol of glucose. In contrast, in hetero-lactic fermentation, theoretically, only half of the glucose is converted to lactic acid, while the other half is converted into by-products like ethanol, so the yield does not exceed 0.5 mol/mol glucose. Therefore, a lactic acid fermentation process with a glucose conversion rate exceeding 0.5 mol/mol would be considered homolactic.

Our L-valine conversion rate reached 0.55 g/g glucose, which is equivalent to 0.85 mol/mol of glucose, close to the theoretical conversion rate of 1 mol/mol of glucose. Furthermore, we did not detect any significant metabolite that *E.coli* typically produces such as acetate, formate, ethanol, lactate, succinate, and L-alanine. Therefore, we consider our fermentation process to be homotypic.

We detected the L-alanine, L-isoleucine, L-leucine, and L-threonine. L-leucine accounts for only 0.1 g/L with more than 30 g/L of L-valine production. L-alanine was produced more than 0.1 g/L, is because of the improved LeuDH activity as described in Line 636-641 “Upon reviewing the fermentation results, we observed that L-alanine accumulated around 0.2 g/L at the end of fermentation, exceeding the initial supplement of 0.1 g/L. Therefore, there must be other L-alanine synthesis reactions in our synthesized strains. After conducting a literature search, we found that LeuDH has 3.4% activity with L-Alanine compared to L-Leucine[43]. Given that the $LeuDH^{V22I}$ has higher activity and is present in multiple copies in S2.X strains, it likely catalyzes pyruvate to L-alanine, which potentially imposes a metabolic burden on cell growth (Figure 5).”.

The reason why there is less than 1g/L L-threonine and L-isoleucine is because they can be the extra redox sinks, as we discussed in Line 606-623 in the section Excess NADH in Synthesized L-Valine Strains is Alleviated via the L-Threonine Pathway.

Poorly Structured Introduction

The Introduction lacks clarity and depth, failing to effectively frame the study’s objectives and significance.

Thank you for your suggestion. We have added a section in the Introduction (Line 55-65) explaining the principles of anaerobic rescue and highlighting successful cases to help readers better understand the context and significance of our study.

Severe Manuscript Formatting and Language Issues

The manuscript contains numerous grammatical errors, inconsistent formatting, missing line numbers, and overall poor organization. Such deficiencies do not meet the basic standards of a peer-reviewed submission.

Thanks for the careful review. We have corrected the typos and checked for syntax errors. Hope this time can achieve the standard.

Conclusion

This manuscript presents a conceptually interesting approach but suffers from fundamental flaws in experimental design, data interpretation, and manuscript preparation. The limited applicability of the ALE strategy, poorly controlled evolutionary trajectories, lack of statistical validation, and failure to compare results with existing studies undermine the novelty and significance of the work. Furthermore, the manuscript does not meet the editorial and linguistic standards required for publication in Nature Communications. Given these critical shortcomings, I strongly recommend rejecting this manuscript in its current form.

Dear reviewers,

We sincerely appreciate the constructive feedback provided by the reviewers. Your comments and the resulting revisions have substantially improved the quality and clarity of our manuscript.

Point-by-point responses to all comments (highlighted in blue) are provided, with corresponding revisions clearly marked in red in the manuscript. The manuscript has been thoroughly revised, with additional experiments incorporated where appropriate.

We hope that the revised version now meets the standards for publication.

Sincerely,

Sheng Yang

REVIEWER COMMENTS

Reviewer #1 (Remarks to the Author):

1. "Since the endogenous IlvC (for α-acetolactate reduction) and IlvE (for α-ketoisovalerate transamination) are NADPH-dependent, whereas IlvCTM and LeuDH are NADH-dependent, we introduced two cofactor-balancing enzymes encoded by pntAB and nadK. pntAB encodes pyridine nucleotide transhydrogenase, which can catalyze the reversible transfer between NADH and NADP+ (Figure 1a and Figure 5). nadK encodes NAD+ kinase, which catalyzes the phosphorylation of NAD+ to NADP+ using ATP (Figure 1a and Figure 5). This design lets the strain choose the optimal ratio between NAD(H) and NADP(H)." and "To explain how L-valine production was coupled with cell growth: we deleted all native NADH consuming pathways, so in the absence of an NAD^+ regeneration route, cell growth would be severely impaired or even halted. By overexpressing ilvCTM and leuDH, we enabled the production of L-valine, which provided an NADH sink to relieve the intracellular redox imbalance, thereby linking L-valine biosynthesis to cell growth." There seems to be a contradiction between these two paragraphs. If introducing ilvCTM and leuDH to consume NADH is key for coupling L - valine production with growth under anaerobic conditions, then introducing pntB would also consume NADH. In that case, would introducing pntB to consume NADH affect the coupling of strain growth and production？ Additionally, is NADPH the same as NADH, and would it affect the strain's growth?

We appreciate the reviewer's insightful question regarding the potential contradiction between coupling L-valine production with growth via NADH consumption (through $IlvC^{TM}$ and LeuDH or it's mutant), and the role of *pntAB*, which can also consume NADH.

Ideally, the NADH generated through glycolysis is rapidly consumed by $IlvC^{TM}$ and LeuDH (and potentially also by IlvCCg), allowing for continuous NAD^+ regeneration and maintaining glycolytic flux. However, if the activities of $IlvC^{TM}$ and LeuDH become rate-limiting, NADH may accumulate, leading to a redox imbalance that slows down glycolysis and consequently cell growth.

In this scenario, the reversible transhydrogenase PntAB can convert excess NADH and $NADP^+$ into NAD^+ and NADPH (as shown below and also illustrated in Figures 1a and 5), thereby helping restore NAD^+ levels. The resulting NADPH can then be consumed by endogenous IlvC and IlvE, which also contributes to restoring cofactor balance. Thus, rather than disrupting the coupling of growth and L-valine production, PntAB serves as an auxiliary route for NAD^+ regeneration, especially when $IlvC^{TM}$ and LeuDH alone are insufficient to handle the NADH load.

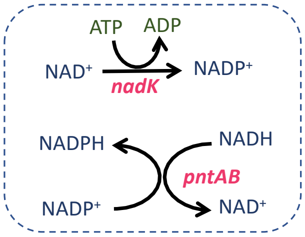

Consistent with this, we did not observe any negative impact of *pntAB* overexpression on the coupling between strain growth and L-valine production.

For the question that "is NADPH the same as NADH, and would it affect the strain's growth?". NADPH and NADH are distinct cofactors, although they are chemically similar. In our system, NADPH is required by the native IlvC and IlvE enzymes, while $IlvC^{TM}$ and LeuDH (and potentially also by IlvCCg) are NADH-dependent. The introduction of *pntAB* and *nadK* allows for flexible redistribution of reducing equivalents between the NAD(H) and NADP(H) pools, facilitating robust redox balance under anaerobic conditions and supporting both cell growth and L-valine biosynthesis. Therefore, it will not affect cell growth.

2. Please list the total amounts of ATP and NADH generated and consumed under anaerobic conditions.

Thank you for your question. We have listed the total amounts of ATP and NADH generated and consumed under anaerobic conditions in Figure 1a, Figure 5, and Supplementary Figure 1. These figures annotate the cofactor usage directly on the metabolic pathways.

To further clarify this point, we have updated the captions of Figure 1a (line 165-167) and Figure 5 (line 412-414) to include the following statement: "A total of two NADH and two ATP molecules are generated, and two NADH molecules are consumed during the biosynthesis of one L-valine molecule from glucose under anaerobic conditions."

Reviewer #2 (Remarks to the Author):

I co-reviewed this manuscript with one of the reviewers who provided the listed reports. This is part of the Nature Communications initiative to facilitate training in peer review and to provide appropriate recognition for Early Career Researchers who co-review manuscripts.

Reviewer #3 (Remarks to the Author):
In the revised version of their manuscript the authors addressed several issues raised in my previous report. However, there are still several points that have not yet been solved in a satisfactory manner.

1) I still find the subsections that go beyond strain E1.0 (text between line 435- 664, which is >50% of the Results section) rather verbose and that they contain no or only little advancements. Indeed, the best strain of this manuscript shown in Table 1 refers to strain E1.0. I would either completely remove this part or heavily condense it (e.g. to one page) to convey only the most relevant and evident findings. For example, the explanation regarding a potential futile cycle between GlcNAc-6-P and GlcNAc remains very vague with only very little evidence supporting this hypothesis. This part should be removed.
We appreciate the reviewer's feedback. In the revised manuscript, we have combined "Rapid reconstruction of the genotype of hyperproducer cells" and "Anaerobic growth rescue of S2.0" into "Rapid construction and anaerobic growth rescue of S2.0", condensed the sections beyond strain E1.0 (previously lines 435–664) by reducing their length from approximately 229 to 193 lines. In the meanwhile, we added explanations according to editor's comment, reviewer 2's comment 2 and your comment 3 in these lines (see Pages 9-15). The most relevant findings have been retained. The speculative explanation regarding a potential futile cycle involving GlcNAc-6-P and GlcNAc has been removed from the Results and relocated to the Discussion section for more appropriate context.

2) Please add information on the used media (minimal media with carbon source + (carbon) additives such as alanine or yeast extract) in Table 1.
Also regarding Table 1, the authors wrote "We report this highest yield reported to date (Table 1), which has reduced the production cost of L-valine at Meihua Group significantly, as glucose constitutes the largest portion of the production cost components of L-valine." Does this mean that the process/strain developed by the authors is now used by the Meihua group ("has reduced"!)? Otherwise please rephrase!
Thank you for your helpful suggestions. We have now added detailed information regarding the media composition (e.g., minimal medium, carbon sources, and additives such as alanine or yeast extract) in Table 1, as requested. Please see the updated Table 1 appended below for your reference.

Table 1 Comparison of L-valine-producing strains.

Host	Culture condition in bioreactor*	Performance			Reference
		Titer	Yield	Rate	
		g/L	g/g glucose	g/L/h	
***E.coli* W**	Fed-batch fermentation was carried out in a 6.6-L bioreactor supplemented with 2 g/L yeast extract and 2 mM L-isoleucine.	60.7	0.22	2.06	Ref 6
***E.coli* W3110**	Two-stage aerobic-oxygen limiting fed-batch fermentation was performed in a 5-L bioreactor, with 5 g/L yeast extract added to the medium.	84	0.41	2.33	Ref 11
***E.coli* ATCC 8739**	Anaerobic fermentation was conducted in a 320-m³ bioreactor, with 0.7 g/L yeast extract, 0.6 g/L betaine hydrochloride, and 0.36 g/L L-alanine.	83.6	0.55	1.39	This study
***E.coli* ATCC 8739**	Anaerobic fed-batch fermentation was performed in a 320-m³ bioreactor with limited oxygen bubbling during the initial 0–4 hours. The medium was supplemented with 0.7 g/L yeast extract, 0.6 g/L betaine hydrochloride, and 0.36 g/L L-alanine.	82.6	0.56	1.84	This study
C. glutamicum	Aerobic fed-batch fermentation was conducted in a 320-m³ bioreactor, supplemented with 50 g/L corn steep liquor, 2 g/L yeast extract, and 0.4 g/L soybean hydrolysate.	85	0.37	1.25	A commercial aerobic process (data provided by Meihua Holdings Group)

* We considered only these strains with available data from fermentation experiments in bioreactors.

We also revised the sentence regarding Meihua Group in line 522–524 to improve clarity as:

This strain is currently being evaluated at 320 m^3 scale by Meihua Group, where it has significantly reduced L-valine manufacturing costs, as glucose accounts for the largest share of total expenses.

3) The authors responded satisfactorily to my question why threonine must be produced by the cells (redox balancing due to excess of reduction equivalents synthesized during growth) – this explanation (maybe in a slightly condensed version) should also be added to the manuscript.
Thank you for the helpful suggestion. In response, we have incorporated a condensed version of our explanation into the manuscript at lines 402–408 as follows:

This raises the question of why *thrA* mutations evolved to consume excess NADH, despite the engineered L-valine pathway being redox-balanced. The likely explanation is that biomass synthesis in *E. coli* inherently produces a slight NADH surplus, even under redox-balanced conditions. In our case, although excess NADH was minimal, mutations in *thrA*, including a promoter G→A change and an S354Y substitution, relieved L-threonine feedback inhibition, enabling low-level L-threonine synthesis to act as a redox sink. This represents a unique and elegant evolutionary solution to a redox challenge in a system engineered for homotypic L-valine production.

We appreciate the reviewer's insight, which helped us improve the clarity of this section.

4) One thing that I did not mention in my first review: I think it is mandatory and standard to provide (for the best strain E1.0) the specific growth rate, specific valine production rate and specific glucose uptake rate (at least for the exponential growth phase) to be able to assess the specific productivity of the evolved strain. I also agree with reviewer 4 that valine production should be shown for the entire time period (not only for >12h) in Fig. 1e.

Thank you for helping us polish the manuscript. We collected OD_{600}, L-valine, and glucose concentrations hourly for the first 20 hours in another batch of the 320 m^3 fermentation. The updated Figure 1E has now been replaced accordingly, as shown below:

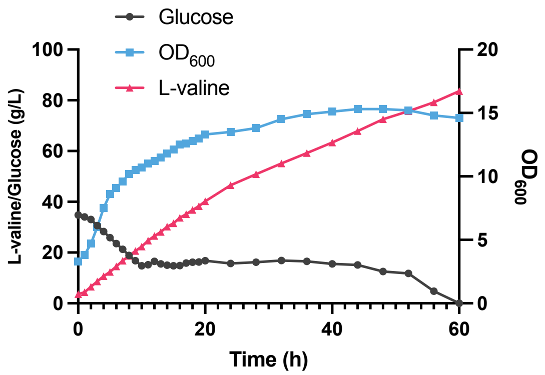

Accordingly, we calculated the the specific growth rate, specific valine production rate and specific glucose uptake rate for the exponential growth phase and added these data in Line 207-209 as: During the exponential phase (1-6 h), the specific growth rate was 0.183 h^{-1}, the specific L-valine production rate was 9.48 mmol/gDCW/h and the specific glucose uptake rate was 6.41 mmol/gDCW/h (Conversion factor, 1 OD_{600} = 0.275 gDCW/L, was used)[6].

6) Further language improvements are required.

We appreciate the reviewer's suggestion regarding language clarity and style. In response, we have carefully revised the manuscript to improve grammar, readability, and overall flow. We conducted a thorough round of editing and polishing, including improvements to sentence structure and clarity throughout the manuscript.

We hope the revised version now reads more clearly and fluently.

Point-to-point response

Reviewer #3 (Remarks to the Author):

The authors addressed further points raised in my review of the second round, though not all of them . In particular, the discussion on the very hypothetical GlcNAc / GlcNAc-6P cycle is still part of the manuscript (now moved to Discussion section).

A new major issue emerged in connection with the changes introduced by the authors: As requested, the authors added the full time course of the valine concentration in Figure 1e. With that, I discovered a serious inconsistency: after about 10 hours, the glucose concentration is approximately 18 g/L and the valine concentration ca. 28 g/L. For the following 35 h (until 45 h) almost no glucose is consumed while the valine concentration increases steadily with high rates up to about 65 g/L. How can this be, what is the carbon source for valine production in that period? Also, when dividing the final valine titer (83.g/L) by the initial glucose concentration (ca. 38 g/L) the valine yield is above 2 g/g and thus far above the maximum yield of 0.65 g/g. Again, how can this happen? Similar inconsistencies can be seen in the time course in Supplementary Figure 4d.

1. We appreciate your thoughtful suggestion. In response to the concern of "very hypothetical" discussion about GlcNAc / GlcNAc-6P cycle, we have completely removed it from the revised manuscript. The discussion part (line 506-508) now is shown as follows:

These five variants, along with the *nagA* variant and amplification of the main L-valine biosynthetic pathway genes and optimization of cofactor balance, constitute the minimal set of variants required for L-valine anaerobic production (Figure 5).

2. Thank you for focusing on this important point. We apologize for the confusion caused by the way the data was presented. The apparent inconsistency arises from the fact that the fermentation was conducted in a fed-batch mode with concentrated glucose feeding, which was not sufficiently emphasized in the figure legend and comprehensively described in the methods.

To avoid further confusion, we have revised the manuscript to clearly describe the glucose feeding strategy. Specifically, we replaced the former description (Lines 646–648: *A 60–62% (w/v) glucose solution was added to replenish glucose before depletion, ensuring it was fully consumed by the end of the 60-hour fermentation cycle.*) with a more detailed account of the fed-batch glucose feeding in the Methods section (Lines 637–640). In addition, the legend of Figure 1e (Lines 176–179) has been updated accordingly as follows:

When the residual glucose concentration reached ~15 g/L (9–10 h), a 60–62% (w/v) glucose solution was fed at 0.5–1.5 m^3/h to maintain a 10–15 g/L residual glucose level. Feeding was terminated at 52–54 h, and residual glucose was fully consumed at the end of fermentation (60 h).

Since Figure 1e represents the optimized process and differs from the earlier fed-batch procedure shown in Supplementary Figure 4d, we have now removed Supplementary Figure 4d to make the manuscript more concise and easier to follow.

3. You are correct that dividing the final valine titer (83 g/L) by the initial glucose concentration (~38 g/L) would yield a value above the theoretical maximum. However, this calculation does not account for the glucose supplied during the fed-batch phase, due to our overly simplified presentation of data. In actual fact, the total sugar input, including initial charge and feeding, was 38.17 t, leading to a total L-valine output of 20.98 t and an overall conversion yield of 0.55 g/g. The detailed calculations are provided in Supplementary Table 12 (cited in Line 205) and also as follows:

Stage	Glucose concentration (g/L)	Volume (m^3)	Glucose added (t)
Secondary seed	30	3	0.09
Tertiary seed	30	42	1.26
Fermenter base	30	200	6
Fed-batch feeding	605	50.95	30.82
Total sugar	—	—	38.17

L-valine production

Final titer of L-valine = 83.6 g/L

Broth volume = 250.95 m^3

Total L-valine= 83.6 g/L × 250,950 L ÷ 1000 = 20.98 t

Overall yield

Rate = 20.98 t (L-valine) ÷ 38.17 t (glucose)= 0.5496 g/g

We hope this revised version with detailed information provides the reviewer with much greater clarity .

Reviewer #4 (Remarks to the Author):

The revised manuscript claims to demonstrate anaerobic “homotypic L-valine fermentation” in E. coli, achieving titers above 80 g/L with near-theoretical yields. While this would represent a significant advance, the data presented remain internally inconsistent and scientifically implausible.

In particular, Figure 1e shows ~80 g/L valine accumulation while only ~30 g/L glucose is consumed. Moreover, between 10–50 h, ~50 g/L valine is reported to be produced despite negligible glucose consumption. These results are incompatible with basic stoichiometric and redox balances and raise serious concerns about data reliability. The authors’ rebuttal did not provide satisfactory clarification or corrected datasets.

Additional contradictions regarding redox balancing (IlvCTM/LeuDH vs. pntAB/nadK roles) and the overextended discussion of strains beyond E1.0 further weaken the manuscript. Given these

unresolved issues and the lack of verifiable evidence supporting such extraordinary fermentation performance, I cannot recommend this work for publication in Nature Communications.

1. We sincerely thank you for the careful examination of our work and for pointing out the concerns regarding data consistency.

The apparent inconsistency arises from the fact that the fermentation was conducted in a fed-batch mode with concentrated glucose feeding, which was not sufficiently emphasized in the figure legend and detailedly described in the methods.

To avoid further confusion, we have revised the manuscript to clearly describe the glucose feeding strategy. Specifically, we replaced the former description (Lines 646–648: *A 60–62% (w/v) glucose solution was added to replenish glucose before depletion, ensuring it was fully consumed by the end of the 60-hour fermentation cycle.*) with a more detailed account of the fed-batch glucose feeding in the *Methods* section (Lines 637–640). In addition, the legend of Figure 1e (Lines 176–179) has been updated accordingly as follows:

When the residual glucose concentration reached ~15 g/L (9–10 h), a 60–62% (w/v) glucose solution was fed at 0.5–1.5 m^3/h to maintain a 10–15 g/L residual glucose level. Feeding was terminated at 52–54 h, and residual glucose was fully consumed at the end of fermentation (60 h).

2. You are correct that dividing the final valine titer (~80 g/L) by the initial glucose concentration (~30 g/L) would yield a value above the theoretical maximum. However, this calculation does not account for the glucose supplied during the fed-batch phase,due to our overly simplified presentation of data. In actual fact, the total sugar input, including initial charge and feeding, was 38.17 t, leading to a total L-valine output of 20.98 t and an overall conversion yield of 0.55 g/g. The detailed calculations are provided in Supplementary Table 12 (cited in Line 205) and also as follows:

Stage	Glucose concentration (g/L)	Volume (m^3)	Glucose added (t)
Secondary seed	30	3	0.09
Tertiary seed	30	42	1.26
Fermenter base	30	200	6
Fed-batch feeding	605	50.95	30.82
Total sugar	—	—	38.17

L-valine production

Final titer of L-valine = 83.6 g/L

Broth volume = 250.95 m^3

Total L-valine= 83.6 g/L × 250,950 L ÷ 1000 = 20.98 t

Overall yield

Rate = 20.98 t (L-valine) ÷ 38.17 t (glucose)= 0.5496 g/g

3. We thank you for raising up the question regarding the contradictions in redox balancing (IlvCTM/LeuDH vs. pntAB/nadK roles), which was also noted by Reviewer 1 previously.

The reason we did not predetermine a specific redox recycling pathway due to the inherent unpredictability of the strain's preferred route under anaerobic conditions. Instead, we allowed the evolutionary process to select the most efficient pathway.

Therefore, we have explained this in the previous version of our manuscript in Line 150-156: “Since the endogenous IlvC (for α-acetolactate reduction) and IlvE (for α-ketoisovalerate transamination) are NADPH-dependent, whereas IlvCTM and LeuDH are NADH-dependent, we introduced two cofactor-balancing enzymes encoded by *pntAB*(9,30)and *nadK*(31,32). *pntAB* encodes pyridine nucleotide transhydrogenase, which can catalyze the reversible transfer between NADH and NADP(+) (Figure 1a and Figure 5). *nadK* encodes NAD(+) kinase, which catalyzes the phosphorylation of NAD(+) to NADP(+) using ATP (Figure 1a and Figure 5). This design lets the strain choose the optimal ratio between NAD(H) and NADP(H). "

To explain how L-valine production was coupled with cell growth, we deleted all native NADH-consuming pathways, so in the absence of an NAD^+ regeneration route, cell growth would be severely impaired or even halted. By overexpressing *ilvC(T)*(M) and *leuDH*, we enabled the production of L-valine, which provided an NADH sink to relieve the intracellular redox imbalance, thereby linking L-valine biosynthesis to cell growth. Regarding the strain design principle, we have added relevant content to the Introduction (Line 56-66) to clarify our approach: " Anaerobic growth rescue is a strategy that leverages microorganisms to restore or enhance growth under anaerobic conditions by maintaining intracellular redox balance(13). This is generally achieved by coupling the desired reaction(s) to NAD(+) regeneration, thereby alleviating the redox imbalance caused by the inactivation of NADH-consuming pathways(13). This strategy has enabled the transformation of *E. coli* from mixed-acid fermentation(14) to the production of n-butanol(13), D-lactate(15), succinate(16), ethanol(17,18), and L-alanine(19)…… "

And also in results, Line 97-110：

"To leverage anaerobic growth rescue for homotypic L-valine fermentation, the reduction of α-acetolactate and reductive amination/transamination of α-ketoisovalerate represent the two key steps that can consume the NADH generated from glycolysis. To couple L-valine production with NADH oxidation, we….."

Hope these can address your concerns.

Point-to-point response

Reviewer #4 (Remarks to the Author):

Overall Assessment
The revised manuscript entitled "Anaerobic metabolic evolution for homotypic L-valine fermentation" describes metabolic engineering and laboratory evolution strategies in E. coli for achieving high-yield anaerobic L-valine production. The study addresses an important challenge in amino acid biotechnology, and if valid, the reported yields (>80 g/L, ~85% of theoretical maximum) would represent a major breakthrough.
However, despite extensive revisions and a detailed rebuttal, the manuscript continues to present data inconsistencies, stoichiometric implausibilities, and insufficient methodological transparency, which undermine the reliability of the reported claims.

We appreciate Reviewer 4's recognition that, if validated, our results would represent a breakthrough. Regarding the concerns raised by Reviewer 4 about data inconsistency, stoichiometric imbalance, and insufficient methodological transparency, we have addressed each of these points in detail below.

Major Concerns
1. Stoichiometric Inconsistencies in Fed-Batch Data
• Although the rebuttal explains that this is due to fed-batch glucose feeding, the manuscript presentation (residual glucose only) remains misleading. Yield calculations must be based on total glucose consumed, not initial charge or residual concentrations.
• The authors now provide supplementary calculations showing 20.98 t valine from 38.17 t glucose (yield ~0.55 g/g). However, these data were not transparently integrated into the main figures, and earlier inconsistencies (e.g., Supplementary Figure 4d) were simply removed rather than corrected.
Recommendation: The manuscript must include (i) complete time-course profiles of glucose consumed (not just residual), (ii) corresponding feeding rates, and (iii) reconciled carbon and redox balances. Without this, the data remain unreliable.

Thank you for your suggestions. We removed the original Supplementary Figure 4d because this dataset did not include the 0–10 h record previously requested by Reviewer 3, as such monitoring was not required routinely at the pilot production stage. Therefore, detailed feeding/consumption records for this particular batch are also unavailable. However, the glucose feeding/consumption data for Supplementary Figure 4d are broadly consistent with those presented in Figure 1e. To clarify that our intention was not to conceal any information, we have reinstated this figure as Supplementary Figure 4d, and in the legend we have provided the total consumption data as follows:

"For this batch, the feeding glucose concentration was 600 g/L, with a total feeding volume of 49.5 m^3. The total glucose consumption was 37.05 t, the total valine production was 20.48 t, corresponding to an overall yield of 0.5528 g/g."

We also described the details for Supplementary Figure 4d in Methods in Line 649-652 as follows: "The feeding details for Supplementary Figure 4d are as follows: the feeding glucose concentration was 600 g/L, with a total feeding volume of 49.5 m^3. The total glucose consumption was 37.05 t, and the total L-valine production was 20.48 t, corresponding to an overall yield of 0.5528 g/g."

To strengthen the transparency and credibility of our results, we have included a third independent batch (now shown in Supplementary Figure 4e and cited in Line 217) for which complete feeding and consumption profiles were recorded. We fully address points (i) and (ii) raised by the reviewer for the second and third batches. The yields calculations of three batches are shown in Supplementary Table 12. We believe this additional evidence demonstrates the reproducibility and reliability of our industrial fermentation data.

The details are as follows:

(i) We have added Table 2 and 3 in Methods section in Line 655-657 and shown as follows. Based on these data, we integrated the complete time-course profiles of glucose consumed into Figure 1e and Supplementary Figure 4e(shown in ii):

Table 2 Glucose Feeding schedule for Figure 1e

Time period (h)	Duration (h)	Feeding rate (m^3/h)	Feeding rate (g/h)
9–12	3	0.5	302500
12–16	4	0.8	484000
16–24	8	1.2	726000
24–40	16	1.5	907500
40–44	4	1.2	726000
44–48	4	1.0	605000
48–52	4	0.7	423500
52–54	2	0.525	317625

Table 3 Glucose Feeding schedule for Supplementary Figure 4e

Time period (h)	Duration (h)	Feeding rate (m^3/h)	Feeding rate (g/h)
9–11	2	0.5	302500
11–15	4	0.8	484000
15-16	1	1.0	605000
16-24	8	1.2	726000
24-40	16	1.5	907500
40–44	4	1.2	726000
44–48	4	1.0	605000
48–51	3	0.7	423500
51–53	2	0.5	317625

(ii) We have also updated the feeding rate in the figures (according to column 4 of the Table 2 and 3 above). To make the data more interpretable, we use the metrics g for Glucose consumed instead of t, g/L for titer of L-valine and Glucose remaining, and g/h for Glucose feeding rate instead of m^3/h.

The revised Figure 1e is shown below:

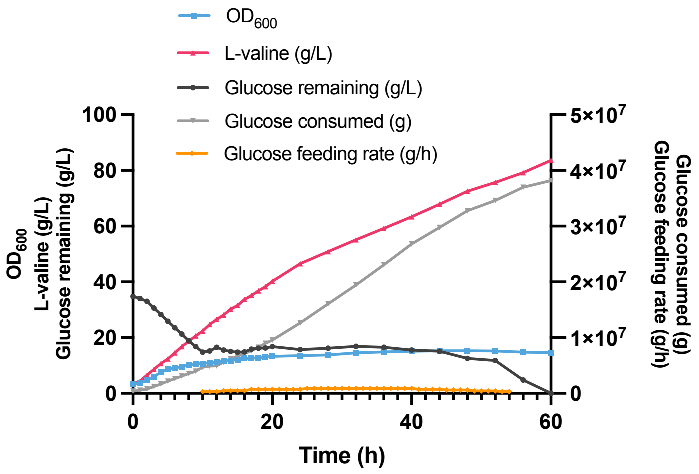

The newly added Supplementary Figure 4e is shown as:

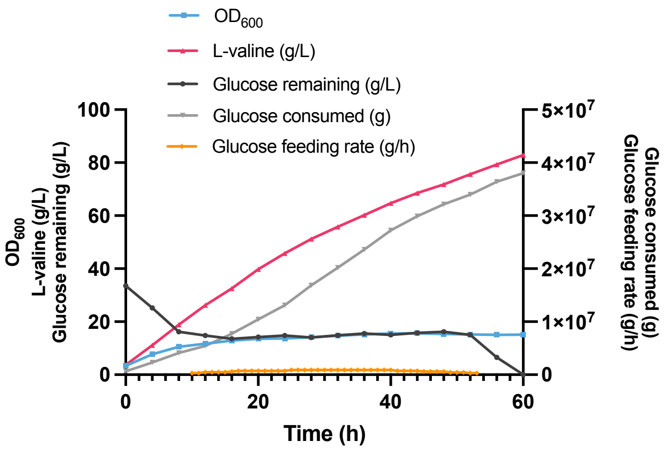

* The fermentation process for this batch was initiated prior to the subsequent request from Reviewer 3 for comprehensive hourly records (0-10 h), hence the lack of detailed data for this specific time frame.

(iii) Reconciled carbon and redox balances：

Our yield reached 85% of the theoretical value. While this appears strikingly high compared to previously reported aerobic processes, it is consistent with the characteristics of anaerobic fermentation. For example, anaerobic production of n-butanol (88% of the theoretical, Ref. 13),

D-lactate (93% of the theoretical, Ref. 15), L-alanine (95% of the theoretical, Ref. 19), and isobutanol (92% of the theoretical, Ref. 25) all exceed 85% of the theoretical yield.

References:
13. Shen CR, Lan EI, Dekishima Y, Baez A, Cho KM, Liao JC. Driving Forces Enable High-Titer Anaerobic 1-Butanol Synthesis in *Escherichia coli*. Applied and Environmental Microbiology 77,2905-2915 (2011). https://doi.org/10.1128/AEM.03034-10
15. Zhou, S., Shanmugam, K.T., Yomano, L.P. et al. Fermentation of 12% (w/v) Glucose to 1.2 m Lactate by *Escherichia coli* Strain SZ194 using Mineral Salts Medium. Biotechnol Lett 28, 663–670 (2006). https://doi.org/10.1007/s10529-006-0032-5
19. Zhang, X., Jantama, K., Moore, J.C. et al. Production of l-alanine by metabolically engineered *Escherichia coli* . Appl Microbiol Biotechnol 77, 355–366 (2007). https://doi.org/10.1007/s00253-007-1170-y
25. Shi A, Zhu X, Lu J, Zhang X, Ma Y. Activating transhydrogenase and NAD kinase in combination for improving isobutanol production. Metab Eng 16, 1-10 (2013). https://doi.org/10.1016/j.ymben.2012.11.008

NADH oxidation is efficiently coupled with L-valine biosynthesis, and we consider the outcomes of anaerobic evolutionary adaptation (Figure 1c, d) to be the strongest evidence for this coupling. We have also further addressed your related comments in major point 3 below.

---

2. Data Credibility and Removal of Figures
• The removal of Supplementary Figure 4d (where inconsistencies were most evident) raises concern that the authors are omitting problematic datasets rather than correcting them.
• Extraordinary claims (83.6 g/L valine at ~85% theoretical yield in a 320 m^3 reactor) require extraordinary evidence. At present, the documentation is insufficient.

We totally understand your concerns. We removed the original Supplementary Figure 4d because this dataset did not include the 0–10 h record previously requested by Reviewer 3, as such monitoring was not required at the pilot production stage. Therefore, detailed feeding/consumption records for this particular batch are also unavailable. However, the glucose feeding/consumption data for Supplementary Figure 4d are broadly consistent with those presented in Figure 1e. To clarify that our intention was not to conceal any information, we have reinstated this figure as Supplementary Figure 4d, and in the legend we have provided the total consumption data as follows:

"For this batch, the feeding glucose concentration was 600 g/L, with a total feeding volume of 49.5 m^3 . The total glucose consumption was 37.05 t, the total valine production was 20.48 t, corresponding to an overall yield of 0.5528 g/g."

We also described the details for Supplementary Figure 4d in Methods in Line 649-652 as follows:

"The feeding details for Supplementary Figure 4d are as follows: the feeding glucose concentration was 600 g/L, with a total feeding volume of 49.5 m^3 . The total glucose

consumption was 37.05 t, and the total L-valine production was 20.48 t, corresponding to an overall yield of 0.5528 g/g."

To strengthen the transparency and credibility of our results, we have included a third independent batch (now shown in Supplementary Figure 4e and cited in Line 217) for which complete feeding and consumption profiles were recorded (see Major 1). The yields calculations of three batches are shown in Supplementary Table 12. We believe this additional evidence demonstrates the reproducibility and reliability of our industrial fermentation data.

3. Redox Balancing Contradictions

• The role of IlvCTM/LeuDH (NADH-dependent) vs. pntAB/nadK (NADPH balancing) remains contradictory. The rebuttal argues that evolution selected the optimal redox pathway, but this explanation is speculative without experimental validation.

• Transcriptomic or flux data are needed to support claims that NADH oxidation was effectively coupled to valine synthesis.

Thanks for addressing this point again. In L-valine biosynthesis, the steps catalyzed by IlvC and IlvE consume NADPH, whereas the glycolytic pathway from glucose to pyruvate primarily generates NADH. To address this redox imbalance, we introduced an NADH-dependent LeuDH for the IlvE step and an NADH-dependent IlvCTM for the IlvC step. We understand your concern: if we already introduced the NADH-dependent IlvCTM/LeuDH, why did we also include the NADPH-balancing modules *pntAB* and *nadK*, which may appear contradictory.

However, in the evolved strain E1.0 we observed gene dosage amplification of *leuDH*, *ilvC,* and *ilvE*, but not of *ilvC*^{TM}, suggesting that:

i) When NADPH supply is secured by high-activity *pntAB*/*nadK*, the native NADPH-dependent IlvC is more advantageous than IlvC^{TM} (hence the amplification of *ilvC* but not *ilvC*^{TM});

ii) Based on this, we infer that *ilvE* may have been passively co-amplified along with *the ilvXGMEDA-ilvC* cluster, whereas the NADH-dependent LeuDH acquired activity-enhancing mutations and was amplified to two copies, thereby becoming more advantageous than IlvE.

Taken together, we conclude that the evolved valine biosynthetic pathway exhibits a cofactor preference that simultaneously exploits both NADH and NADPH. The selective amplification of these genes supports the hypothesis that evolution favored an optimal redox pathway. Moreover, the increased gene dosage (and likely elevated transcription) of L-valine biosynthetic genes, together with the results in Figure 1c, d (showing that faster anaerobic growth correlates with higher L-valine production), provide strong evidence that NADH oxidation is efficiently coupled to L-valine biosynthesis.

---

4. Overextension Beyond E1.0

• The manuscript devotes significant space to later strains (E2.5, S2.0, etc.), which distracts from the central claim of demonstrating homotypic valine fermentation.
• Given the unresolved issues in E1.0 datasets, extensive discussion of derivative strains weakens the narrative rather than strengthening it.

We fully understand the reviewer's concerns. The goal of this study was to reconstruct from scratch a minimal version of E1.0 and to investigate the mechanisms underlying the minimal set of mutations. Although we have not yet been able to fully elucidate all mechanisms, for example, how the *nagA* mutation specifically contributes to enhanced production, we believe that the efforts made, and the findings obtained remain of significant value and should not be disregarded. We hope you can appreciate our position. If possible, we would also greatly appreciate your guidance on how we might streamline and adjust the manuscript to better meet the journal's publication standards.

Minor Points
1. Clarity of Methods – The fed-batch operation must be fully described in the Methods with feeding volumes, timings, and glucose concentrations.

We thank the reviewer for this important suggestion. We have now fully described the fed-batch operation in the Methods section, including the feeding volumes, timings, and glucose concentrations in Line 649-657:

The feeding details for Supplementary Figure 4d are as follows: the feeding glucose concentration was 600 g/L, with a total feeding volume of 49.5 m^3. The total glucose consumption was 37.05 t, and the total L-valine production was 20.48 t, corresponding to an overall yield of 0.5528 g/g.

The feeding details for Figure 1e and Supplementary Figure 4e are provided in Table 2 and 3. The feeding glucose concentration were 605 g/L.

Table 2 Glucose Feeding schedule for Figure 1e

Time period (h)	Duration (h)	Feeding rate (m^3/h)	Feeding rate (g/h)
9–12	3	0.5	302500
12–16	4	0.8	484000
16–24	8	1.2	726000
24–40	16	1.5	907500
40–44	4	1.2	726000
44–48	4	1.0	605000
48–52	4	0.7	423500
52–54	2	0.525	317625

Table 3 Glucose Feeding schedule for Supplementary Figure 4e

Time period (h)	Duration (h)	Feeding rate (m^3/h)	Feeding rate (g/h)
9–11	2	0.5	302500
11–15	4	0.8	484000
15-16	1	1.0	605000
16-24	8	1.2	726000
24-40	16	1.5	907500
40–44	4	1.2	726000
44–48	4	1.0	605000
48–51	3	0.7	423500
51–53	2	0.5	317625

2. Terminology – The term "homotypic fermentation" is not standard and should be more rigorously defined.

Thank you for this suggestion. We have clarified the definition of "homotypic L-valine fermentation" at its first appearance in the Introduction (Page 3, footer, line 69-71) as follows:

In homolactic fermentation, the only product is (S)-lactate, and thus one molecule of glucose is ultimately converted to two such molecules. Inspired by this, we define 'homotypic fermentation' as a fermentation process in which the metabolic flux is redirected such that a single desired product becomes the predominant, and often exclusive, terminal product.

3. Discussion – The speculative mechanistic explanations (e.g., gain-of-function mutations in AlaE) are interesting but would benefit from stronger experimental validation.

Thank you for the suggestion. We have added the following discussion in the manuscript in Line 508-510:

From the genetic evidence, AlaE appears to exhibit the characteristics of a gain-of-function mutation, which would be more convincingly demonstrated by future biochemical validation.

Recommendation

Despite substantial revisions, the manuscript still suffers from fundamental inconsistencies in mass balance and data presentation. Until complete feeding/consumption profiles and reconciled stoichiometric analyses are transparently reported, the extraordinary fermentation performance claims cannot be substantiated.

Thank you for the recommendation. We have added the complete feeding/consumption profiles and presented an additional batch of fermentation data to ensure that no calculation errors are present.

Point to point response:

Reviewer #4 (Remarks to the Author):

The revised manuscript makes significant claims of >80 g/L L-valine production at ~85% theoretical yield, but key concerns remain. In particular, OD_{600} values continue rising between 9–50 h despite large-volume glucose feeding (~50 m^3), where a dilution effect would be expected; the authors must explain how such biomass growth is possible. Yield and carbon balance reconciliation is incomplete, with insufficient integration of cumulative glucose consumption, redox balance, and biomass measurements. The redox coupling rationale (NADH vs. NADPH) remains speculative without flux or transcriptomic validation. Given the extraordinary nature of the claims, extraordinary evidence is required, and at present the manuscript does not provide sufficient transparency or experimental validation to ensure reproducibility.

We sincerely thank Reviewer 4 for this assessment. We carefully addressed each concern as follows.

(1) OD_{600} increase between 9–50 h despite large-volume feeding

Since the process was conducted without aeration, volume loss due to evaporation was negligible; thus, the final volume can be considered as the sum of the initial feed and the cumulative glucose feeding volume. In Supplementary Table 12, we have added volumetric data according to the feeding schedule provided in Table 2 and 3 in the previous manuscript for the fermentation process. The results show that after glucose feeding, the total fermentation volume increased by approximately 25.5% (from 200 m^3 to 251 m^3) compared to the initial charge.

Glucose feeding was initiated when the residual glucose concentration decreased to 10–15 g/L (around the 9th hour). Under these conditions, biomass accumulation was expected to continue. Therefore, the observed increase in OD_{600} can be attributed to the biomass accumulation rate exceeding the dilution rate of the culture.

(2) Incomplete yield and carbon balance reconciliation with insufficient integration of cumulative glucose consumption, redox balance, and biomass measurements

In the previous version of the manuscript, we already presented glucose consumption and biomass (OD_{600}) data in Figure 1e and Supplementary Figure 4e.

Across representative anaerobic *E. coli* studies (including D-lactate, succinate, n-butanol, isobutanol, and L-alanine production; Refs. 1–9), all of these studies report only the final specific yield value of their respective strains.

In these studies, redox coupling was generally inferred from stoichiometric modeling where available, and none of the studies provided carbon reconciliation or redox balance analyses.

If the reviewer has specific expectations regarding the format or scope of data integration, we would greatly appreciate clear guidance so that we can supplement the analysis accordingly in subsequent small-scale fermentations.

(3) Redox coupling rationale (NADH vs. NADPH) remains speculative without flux or transcriptomic validation

We have carefully reviewed E. coli anaerobic fermentation studies comparable to our system (References 1–9) . Even in the few cases where metabolic maps or stoichiometric summaries were provided, none include redox coupling evidence. Moreover, none of these reports employed transcriptomic or flux analyses to verify redox coupling relationships. Although Ref. 7 reported transcriptomic data, it was used only to illustrate transcriptional upregulation of specific enzymes, not to support redox coupling.

If more direct experimental evidence is considered necessary, we are open to conducting additional transcriptomic or flux-related analyses in small-scale under your guidance to clarify redox coupling mechanisms.

Finally, Figures 1e and Supplementary Figure 4e have been revised according to the editor's suggestions for unit consistency. For Supplementary Figure 4d, detailed feeding and consumption records for this specific batch are unavailable, as such monitoring was not routinely performed during the pilot production stage. Therefore, the parameters for Supplementary Figure 4d cannot be converted into gram (g) units. All revised materials have been uploaded in the resubmission package.

References:

1. Shen CR, Lan EI, Dekishima Y, Baez A, Cho KM, Liao JC. Driving Forces Enable High-Titer Anaerobic 1-Butanol Synthesis in Escherichia coli. Applied and Environmental Microbiology 77, 2905-2915 (2011). (Ref 13 in our manuscript)
2. Zhou S, Shanmugam KT, Yomano LP, Grabar TB, Ingram LO. Fermentation of 12% (w/v) Glucose to 1.2 m Lactate by Escherichia coli Strain SZ194 using Mineral Salts Medium. Biotechnology Letters 28, 663-670 (2006). (Ref 15 in our manuscript)
3. Zhang X, Jantama K, Moore JC, Jarboe LR, Shanmugam KT, Ingram LO. Metabolic evolution of energy-conserving pathways for succinate production in Escherichia coli. Proceedings of the National Academy of Sciences of the United States of America 106, 20180-20185 (2009). (Ref 16 in our manuscript)
4. Zhang, X., Jantama, K., Moore, J.C. et al. Production of l-alanine by metabolically engineered *Escherichia coli* . Appl Microbiol Biotechnol 77, 355–366 (2007). (Ref 19 in our manuscript)
5. Shi A, Zhu X, Lu J, Zhang X, Ma Y. Activating transhydrogenase and NAD kinase in combination for improving isobutanol production. Metab Eng 16, 1-10 (2013). (Ref 25 in our manuscript)
6. Jantama K, et al. Combining metabolic engineering and metabolic evolution to develop nonrecombinant strains of Escherichia coli C that produce succinate and malate. Biotechnology and Bioengineering 99, 1140-1153 (2008). (Ref 37 in our manuscript)
7. Zhu XN, Tan ZG, Xu HT, Chen J, Tang JL, Zhang XL. Metabolic evolution of two reducing equivalent-conserving pathways for high-yield succinate production in Escherichia coli. Metab Eng 24, 87-96 (2014).

8. Zhang X, Jantama K, Shanmugam KT, Ingram LO. Reengineering Escherichia coli for Succinate Production in Mineral Salts Medium. Appl Environ Microbiol 75, 7807-7813 (2009).
9. Tan Z, Zhu X, Chen J, Li Q, Zhang X. Activating Phosphoenolpyruvate Carboxylase and Phosphoenolpyruvate Carboxykinase in Combination for Improvement of Succinate Production. Applied and Environmental Microbiology 79, 4838-4844 (2013).

Reviewer #1 (Remarks to the Author):

Through comprehensive carbon recovery analysis and stoichiometric redox balancing, this study demonstrates the feasibility of the anaerobic fermentation system with respect to mass and energy conservation. Addressing the dynamics of cellular biomass, this study implemented a two-stage strategy comprising initial aerobic fermentation followed by anaerobic maintenance, which elevated the cellular biomass density while conforming to both physiological principles and process engineering fundamentals. Collectively, the viability of anaerobic L-valine production rests upon stoichiometric constraints (carbon balance and redox homeostasis).

The authors have undergone multiple rounds of revision, supplementing the experiment with additional technical experiments and corresponding mechanistic analyses. As a result, the content and data are now more comprehensive than before.
Therefore, I recommend acceptance of this manuscript.

Reply:

We sincerely thank the reviewer for the positive evaluation of our work and for recognizing the improvements made during the revision process.

We are pleased that the reviewer finds the revised manuscript to be more comprehensive and supports its acceptance.

Thank you again for your constructive feedback.
